# The Spectrum of Doubly Ionized Tungsten (W III)

**DOI:** 10.6028/jres.094.023

**Published:** 1989

**Authors:** L. Iglesias, M. I. Cabeza, F. R. Rico, O. Garcia-Riquelme, V. Kaufman

**Affiliations:** Instituto de Optica, Serrano 121. 28006—Madrid ( Spain); National Institute of Standards and Technology, Gaithersburg, MD 20899

**Keywords:** energy levels, parameters, spectra, tungsten, wavelengths

## Abstract

The spectrum of doubly ionized tungsten (W III) was produced in a sliding-spark discharge and recorded photographically on the NIST 10.7-m normal-incidence vacuum spectrograph in the 600–2680 Å spectral region. The analysis has led to the establishment of 71 levels of the interacting 5*d*^4^, 5*d*^3^ 6*s* and 5*d*^2^ 6*s*^2^ even configurations and 164 levels of the interacting 5*d*^3^ 6*p* and 5*d*^2^ 6*s* 6*p* odd ones. A total of 2636 lines have been classified as transitions between the 235 experimentally determined levels. Comparison between the observed levels and those calculated from matrix diagonalizations with least-squares fitted parameters shows an rms deviation of ±87 cm^−1^ for the even configurations and ±450 cm^−1^ for the odd ones.

## 1. Introduction

There are almost no experimental data on spectra or energy level analyses of ions that are isoionic or isoelectronic with W III. Of all the third spectra of the 5*d*-group, only Lu III [1] with one 5*d* electron and Au III [2] with nine 5*d* electrons have been studied. The isoelectronic Ta II has been analyzed [3] and a study of the (5*d +* 6*s*)*^k^* configurations in the second spectra [4] was done. However, no systematic studies of electronic configurations for the third spectra of this group of elements have been reported.

This is the first analysis of the spectrum of doubly-ionized tungsten (W III) to be published. It is a four-electron spectrum and belongs to the third transition group of elements with 5*d*, 6*s*, and 6*p* electrons in the lowest configurations. The even configurations discussed below are 5*d*^4^, 5*d*^3^ 6*s* and 5*d*^2^ 6*s*^2^, and the odd configurations are 5*d*^3^ 6*p* and 5*d*^2^ 6*s*6*p.* The ground term is the ^5^D of the 5*d*^4^ configuration. It is the only term of this configuration that does not overlap with the next even configuration, 5*d*^3^ 6*s.* The overlap of the configurations causes very strong configuration interaction (CI), especially between the odd ones. The level eigenvectors include different terms and configurations and produce a large number of transitions. Thus, W III is quite a complex spectrum.

## 2. Observations

**Figure 1:**
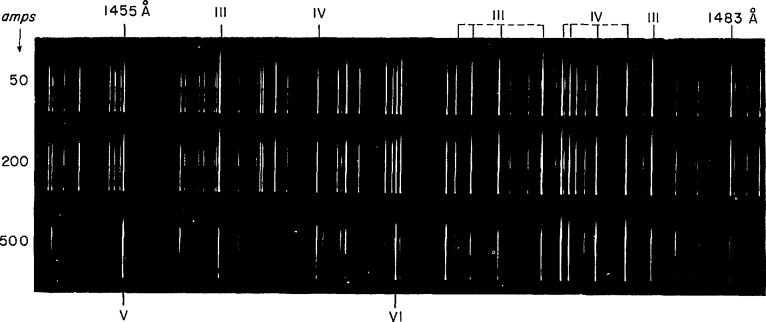
Tungsten spectra.

The spectra were photographed in the region 600–2680 Å with the National Institute of Standards and Technology (NIST) 10.7-m normal-incidence vacuum spectrograph equipped with a 1200-grooves/mm grating and having a plate factor of 0.77 Å/mm. A sliding-spark light source with a quartz spacer was used. Peak currents of approximately 50, 200, and 500 A gave excellent separation of W II, W III, and W IV lines. In order to maintain the discharge, it was necessary to introduce helium at approximately 20 Torr. A water-cooled copper hollow-cathode containing small pieces of germanium and silicon was operated at 500 mA with helium at 2 Torr to produce spectral lines of Cu, Ge, and Si at different ionization stages [5] which were used as reference lines. Part of one of the plates is presented in figure 1. Some of the spectrograms were measured at NIST and the remainder at the Instituto de Optica. The estimated uncertainty in the measurements is ±0.005 Å.

Approximately 3700 lines were identified as belonging to W III, over 1000 lines to W IV [they were the basis of the report entitled “Analysis of the Fourth Spectrum of Tungsten (W IV)”] [6], and about 3500 lines to W II [7]. The analysis of W III has allowed us to classify 2636 lines (73% of the observed lines) as transitions between 71 even levels and 164 odd levels. The unclassified lines in the shorter wavelength region probably correspond to transitions of the 5*d*^3^ 7*p* and 5*d*^3^ 5*f* electron configurations, and the longer wavelength unclassified lines are probably 5*d*^3^ 7*p* and 5*d*^3^ 6*d* transitions.

Table 1 includes all of the W III classified spectral lines, giving for each of them: wavelength (expressed in air above 2000 Å), intensity, wave-number, difference between the observed wavelength and the wavelength obtained from the final level values, and classification. The classification includes the integer portion of the energy level value and the *J* value for each of the two levels. Table 2 contains the wavelength, intensity and wavenumber for each of 953 unclassified lines identified as W III. We have omitted lines with intensities estimated at “1”.

## 3. Analysis

In order to give some idea of the complexity of the electronic structure and the number and the type of levels in the *LS* -coupling scheme, the predicted quintets, triplets, and singlets for the above mentioned configurations are presented in table 3. One should be aware that the coupling is far from pure *LS.* Most of the experimentally determined levels are characterized by a large number of observed transitions. This indicated a strong mixing between different terms and configurations which was later confirmed by the theoretical calculations.

The first goal of the analysis was the determination of the levels corresponding to the ^5^D ground term of the 5*d*^4^ lowest configuration. Then the energy levels of the other configurations could be established relative to the ground state (^5^D_0_=0.00 cm^−1^). The 5*d*^4 5^D ground term is the only one that does not overlap the next even configuration, and it is one of the few terms whose designation in *LS*-coupling is possible. The observed transitions to the even levels with predominantly quintet characteristics, 5*d*^4 5^D and 5*d*^3^(^4^*F*)6*s* ^5^F, assisted in the determination of the 5*d*^3^(^4^*F*)6*p* ^5^G term and the ^5^G_6_ (97039.60 cm^−1^) and ^5^G_5_ (89630.99 cm^−1^) of the 5*d*^2^ 6*s*6*p* configuration. The rest of the levels have no good LS-coupling names.

**Figure 2:**
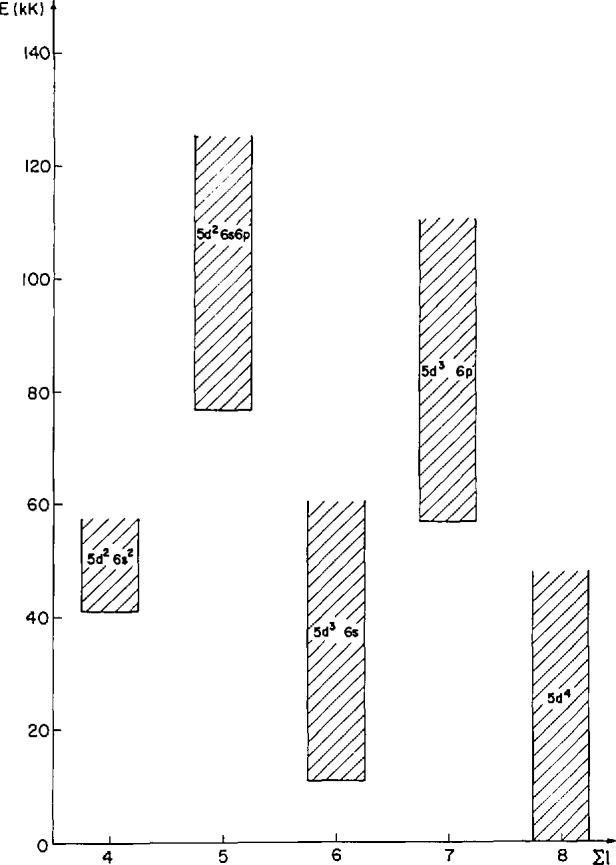
Main configurations of W III.

The energy range of these configurations is shown in figure 2. Tables 4 and 5 give the relevant information about the even and odd levels, respectively. The uncertainties of the optimized energy-level values are generally less than ±0.10 cm^−1^ and no greater than ±0.20 cm^−1^. Included for each level in table 4 are the configuration and term (whenever possible), *J* value, level value, uncertainty, number of observed transitions to or from the level, difference between the observed and calculated energy level, and leading eigenvector percentages in *LS* coupling. Percentages less than 5% have been omitted. Table 5 differs from table 4 in that the first two columns (configuration and term) have been omitted. Only the three first leading percentages, when they are larger than 5%, have been included.

The present situation in the energy-level analysis of W III is shown in table 6. It gives a résumé of the total number of observed and predicted levels, by *J* value, for the even and odd groups of configurations.

## 4. Theoretical Calculations

The low excitation stages of W III correspond to configurations with 5*d*, 6*s*, and 6*p* electrons, giving rise to large numbers of levels. Calculations for several even and odd configurations were carried out using Cowan’s Hartree-Fock program that includes relativistic corrections (HFR) [8]. When the analysis had provided a reasonable number of experimentally derived levels (more than 60%) we were able to try the parametric calculations (least-squares fitting). Several coupling schemes were considered for the even and odd level systems. The average purities obtained in the most representative schemes are:
*LS**JJ*Even53%51%Odd36%35%indicating that no coupling scheme is appropriate to name the levels. Nevertheless, parametric calculations were performed with the use of the *LS* -coupling scheme for both even and odd configurations. As expected, CI plays an important role for the structure of this spectrum.

### 4.1 Even Configurations

The 5*d*^4^ and 5*d*^3^ 6*s* configurations overlap over a wide energy range and, as a consequence, their levels interact very strongly. We first set up one Hamiltonian matrix for these two configurations, including the corresponding CI parameter. The fitting was not at all satisfactory, the mean deviation being greater than ±300 cm^−1^. Because of the unsatisfactory results and with the knowledge that the lowest levels of the next even configuration, 5*d*^2^ 6*s*^2^, appear at about 40000 cm^−1^ overlapping the highest levels of 5*d*^3^ 6*s*, a Hamiltonian matrix including all three even configurations was used. A least-squares fit (LSF), including all of the known 71 even levels resulted in a mean deviation of ±87 cm^−1^ between the observed and calculated values. The resulting LSF parameter values are presented in table 7, in which we also include the HFR values and the LSF/HFR ratios. The *β* parameter of 5*d*^2^ 6*s*^2^ was fixed at zero because the levels that help to determine its value are not known.

The resulting *LS* -percentage compositions of the levels are listed in table 4. More than half of these even levels have been designated in the *LS*-coupling scheme. The remainder have compositions which do not allow us to make any assignments of term or configuration.

### 4.2 Odd Configurations

For the odd configurations, the situation is considerably more complicated. Most of the levels of 5*d*^3^ 6*p* and 5*d*^2^ 6*s*6*p* are so mixed that there is no way of naming the levels. This is especially true for the intermediate and low *J*-valued levels. The matrix for the two configurations included, of course, the corresponding CI parameters. Many attempts were made in order to get a reasonable fit of the parameter values which could provide moderate differences between the observed and calculated energy levels. Although we have used the 164 experimentally determined levels (more than 80% of those predicted for the two odd configurations), the fitting has been almost impossible. This was especially true for the *G*^3^(*dp*) parameters. They were, therefore, fixed at their respective HFR values for the final LSF. Table 8 contains the resulting LSF parameter values, those obtained from the HFR calculations, and the LSF/HFR ratios.

The *LS* -percentage compositions of the levels have been included in table 5. As can be observed for most of the levels, the leading percentages are less than 30, and there are no meaningful configuration assignments. For this reason, we have designated the levels by the energy value expressed in units of cm^−1^.

A mean deviation of ±450 cm^−1^ between the observed and calculated levels was obtained. Some calculated levels differ from the observed ones by about 1000 cm^−1^. We included them in the LSF because they are real energy levels; the large number of observed transitions and the small level uncertainties confirm this.

The question arises whether there are any other odd configurations, e.g., 5*d*6*s*^2^ 6*p*, 5*d*^3^ 7*p* and/or 5*d*^3^ 5f, interacting with the two that we have identified. A study and discussion of the structure of the spectra of the third transition group, especially of configurations involving 5*d*, 6*s*, and 6*p* electrons, by theoreticians would be very helpful for future studies of spectra of the Pt group.

## Figures and Tables

**Table 1:** Classified lines of W III

Wavelength (Å)	Int^a^	Wavenumber (cm^−1^)	O-C (Å)	Classification^b^				Wavelength (Å)	Int^a^	Wavenumber (cm^−1^)	O-C (Å)	Classification^b^			
				Level	*J*	Level	*J*					Level	*J*	Level	*J*
880.205	100	113609.90	.000	7686	4–	121296	4	1185.728	1	84336.37	.004	20696	2–	105033	3
929.408	50	107595.37	.002	13700	4–	121296	4	1187.467	10	84212.87	.001	36467	4–	120680	5
1042.282	1	95943.32	.008	19851	3–	115795	3	1189.502	1	84068.80	.002	13992	2–	98061	2
1048.678	1	95358.16	.003	30507	4–	125865	3	1191.777	1	83908.32	.003	29800	2–	113709	3
1055.883	1	94707.46	.006	26588	4–	121296	4	1192.265	1	83873.97	.006	9904	0–	93778	1
1070.461	1	93417.70	−.001	23317	5–	116735	4	1193.046	2	83819.06	.005	6277	3–	90097	3
1107.468	1	90296.06	−.004	13992	2–	104287	2	1197.048	3b	83538.84	.002	13992	2–	97531	3
1108.812	1	90186.61	−.004	19632	5–	109818	4	1197.221	40	83526.77	.000	37769	4–	121296	4
1109.051	1	90167.18	−.004	20696	2–	110863	2				−.002	13700	4–	97227	5
1114.046	1	89762.90	.004	23080	1–	112844	2	1197.900	2	83479.42	.004	16723	5–	100202	4
1116.809	1	89540.83	.001	7686	4–	97227	5	1198.209	1	83457.89	.001	22212	3–	105670	2
1118.585	2	89398.66	.001	22212	3–	111610	3	1198.423	10	83442.99	.003	13700	4–	97144	4
			−.003	36467	4–	125865	3	1199.594	2b	83361.54	.004	26588	4–	109950	3
1120.067	1	89280.37	−.001	13992	2–	103272	1	1200.828	1	83275.87	.003	19851	3–	103127	3
1120.565	10	89240.70	−.005	24490	2–	113730	1	1201.494	1b	83229.71	.002	26588	4–	109818	4
1121.367	1	89176.87	.003	12881	1–	102058	1	1201.612	1b	83221.54	.007	36226	3–	119448	2
1121.777	1	89144.28	.003	20696	2–	109841	2	1201.638	10	83219.74	.000	28391	3–	111610	3
			−.001	6277	3–	95422	3	1202.004	40	83194.40	.001	19632	5–	102826	4
1124.950	1	88892.84	−.001	18376	4–	107269	3	1202.753	20	83142.59	.001	33631	4–	116774	3
1131.934	1	88344.37	−.001	28391	3–	116735	4	1204.065	2	83051.99	.002	6277	3–	89329	3
1133.406	1	88229.64	−.005	28391	3–	116620	2								
								1204.762	1b	83003.95	.000	13700	4–	96704	4
1134.979	1	88107.36	.009	22955	2–	111063	3	1204.819	2b	83000.02	.001	28062	2–	111063	3
1137.938	1	87878.25	.001	23317	5–	111196	4	1205.751	2	82935.86	.003	12427	2–	95363	1
1140.862	1	87653.02	−.001	17380	4–	105033	3	1207.722	10	82800.51	.000	28062	2–	110863	2
1141.175	2	87628.98	.001	22212	3–	109841	2	1208.041	1	82778.65	−.001	24490	2–	107269	3
1141.201	5	87626.98	−.004	31821	3–	119448	2	1208.974	200	82714.76	.003	22955	2–	105670	2
1141.695	10	87589.07	.005	6277	3–	93867	4	1209.082	20	82707.38	.002	12427	2–	95134	2
1142.358	2	87538.23	−.001	15038	3–	102576	2	1209.262	2	82695.07	.006	20432	4–	103127	3
1145.343	3	87310.09	.000	15038	3–	102348	3	1209.601	40	82671.89	.001	28391	3–	111063	3
1146.102	2	87252.27	.001	18376	4–	105628	4	1209.953	2	82647.84	.002	33972	2–	116620	2
1147.845	1	87119.78	.006	24490	2–	111610	3								
								1210.816	1	82588.93	.004	23080	1–	105670	2
1148.780	5	87048.87	−.001	33631	4–	120680	5				−.005	27252	3–	109841	2
1153.975	1	86656.99	−.001	18376	4–	105033	3	1211.156	1	82565.75	.001	27252	3–	109818	4
1155.103	2	86572.37	.002	20696	2–	107269	3	1211.606	1	82535.08	.003	6277	3–	88813	3
			.002	24490	2–	111063	3	1212.305	5	82487.49	.000	43378	4–	125865	3
1155.979	1	86506.76	.002	16621	2–	103127	3	1212.528	5	82472.32	.001	28391	3–	110863	2
1156.065	1	86500.33	.003	23317	5–	109818	4	1213.539	2	82403.61	.004	4461	2–	86865	1
1159.191	3b	86267.06	−.004	30507	4–	116774	3	1213.674	1	82394.45	−.007	20432	4–	102826	4
1159.716	1	86228.01	.002	30507	4–	116735	4	1214.094	3b	82365.95	−.006	29245	3–	111610	3
1160.188	2	86192.93	.002	19851	3–	106044	3	1214.907	2	82310.83	.001	23317	5–	105628	4
1162.838	10	85996.50	.002	19632	5–	105628	4								
								1216.267	1	82218.79	−.001	28977	4–	111196	4
1162.857	2b	85995.10	.000	29800	2–	115795	3	1216.408	5	82209.26	.002	24490	2–	106699	1
1165.197	3	85822.40	.002	13992	2–	99814	2	1217.071	5	82164.48	.001	33631	4–	115795	3
1165.257	1	85817.98	.002	19851	3–	105670	2	1217.253	1	82152.19	−.001	6277	3–	88429	3
1167.298	20	85667.93	.000	28062	2–	113730	1	1217.540	1	82132.83	.003	2256	1–	84389	1
1167.736	1	85635.79	.003	4461	2–	90097	3	1217.940	1	82105.85	.004	15038	3–	97144	4
1168.057	1	85612.26	.002	20432	4–	106044	3	1218.102	1	82094.93	.007	0	0–	82095	1
1169.267	10	85523.67	.000	31211	5–	116735	4				.006	19851	3–	101947	2
1169.919	1	85476.00	.002	33972	2–	119448	2	1218.350	10	82078.22	.001	22955	2–	105033	3
1170.149	1b	85459.20	.008	24490	2–	109950	3	1218.862	20	82043.74	.001	17380	4–	99424	3
			−.005	9904	0–	95363	1								
								1219.129	1	82025.77	.002	4461	2–	86487	2
1170.331	10	85445.91	.003	17380	4–	102826	4	1219.261	10	82016.89	.001	36226	3–	118243	3
1173.003	30	85251.27	.000	35429	6–	120680	5	1219.884	2	81975.01	.005	39321	3–	121296	4
1173.550	1	85211.54	.009	19851	3–	105064	4	1220.797	20	81913.70	−.003	10968	1–	92882	1
1174.197	1b	85164.58	.005	15038	3–	100202	4	1221.120	3b	81892.03	.004	31838	1–	113730	1
1176.921	2	84967.47	.004	17380	4–	102348	3	1221.149	lb	81890.09	.000	36353	2–	118243	3
1177.133	2	84952.17	.002	31821	3–	116774	3	1222.099	5	81826.43	.002	18376	4–	100202	4
1177.666	5b	84913.72	−.001	31821	3–	116735	4	1222.824	2	81777.92	.004	28062	2–	109841	2
1178.839	30	84829.23	.001	36467	4–	121296	4	1223.299	10	81746.16	.001	23317	5–	105064	4
1179.267	10	84798.44	.002	31821	3–	116620	2	1224.271	1	81681.26	−.001	28062	2–	109744	1
1179.511	2	84780.90	.003	28062	2–	112844	2				−.005	23080	1–	104761	2
1180.195	5	84731.76	.003	28977	4–	113709	3	1224.779	2	81647.38	.004	33972	2–	115619	1
1181.597	2	84631.22	.004	20432	4–	105064	4	1224.845	3	81642.98	.004	7686	4–	89329	3
1181.868	10	84611.82	.002	33631	4–	118243	3	1224.902	2	81639.18	.005	37808	1–	119448	2
			−.007	2256	1–	86867	2	1225.217	10b	81618.19	.000	29245	3–	110863	2
1183.092	10	8452428	.002	14899	3–	99424	3	1227.751	10	81449.74	.005	28391	3–	109841	2
1183.790	10	84474.44	.001	26588	4–	111063	3	1228.655	1	81389.81	.005	10968	1–	92358	2
1184.093	20	84452.83	.002	28391	3–	112844	2	1230.737	3	81252.13	.002	26588	4–	107840	4
1184.401	1	84430.86	.005	7686	4–	92117	4	1232.992	1	81103.53	.001	30507	4–	111610	3
1184.944	20	84392.17	.001	36904	5–	121296	4	1234.230	5	81022.18	.002	31821	3–	112844	2
1184.989	1	84388.97	.004	0	0–	84389	1	1234.489	1	81005.18	.004	31838	1–	112844	2
											−.002	10968	1–	91973	2
1234.985	2	80972.64	.005	28977	4–	109950	3	1256.150	50	79608.33	.005	14899	3–	94508	3
1235.314	lb	80951.08	−.005	20432	4–	101383	3	1256.179	20	79606.49	.001	46259	2–	125865	3
1235.855	10	80915.64	.004	22212	3–	103127	3	1256.720	50	79572.22	.002	19851	3–	99424	3
1235.949	1b	80909.49	.008	16621	2–	97531	3	1256.767	100	79569.24	.004	36226	3–	115795	3
1236.131	10	80897.57	.001	12881	1–	93778	1	1257.096	100	79548.42	.002	28062	2–	107611	2
1236.496	40	80873.69	.000	23317	5–	104191	5	1257.489	100	79523.56	.004	10968	1–	90492	1
1237.508	1b	80807.56	−.001	13700	4–	94508	3	1257.727	50	79508.51	.003	23317	5–	102826	4
1238.498	5	80742.96	.005	7686	4–	88429	3	1257.939	50	79495.11	.004	23080	1–	102576	2
1239.082	1	80704.91	.003	29245	3–	109950	3	1258.221	50b	79477.29	.006	12881	1–	92358	2
1239.333	3	80688.56	.005	30507	4–	111196	4	1258.334	100	79470.16	.004	15038	3–	94508	3
1239.567	5	80673.33	.005	13700	4–	94374	4	1258.660	5	79449.57	.002	28391	3–	107840	4
1240.478	20	80614.09	.005	22212	3–	102826	4	1258.766	100	79442.88	.004	30507	4–	109950	3
1240.527	3	80610.90	.005	20696	2–	101307	1				−.004	36353	2–	115795	3
1240.761	10	80595.70	.002	29245	3–	109841	2	1259.558	5	79392.93	.003	22955	2–	102348	3
1240.874	lb	80588.36	−.001	27252	3–	107840	4	1259.783	1	79378.75	.006	9904	0–	89283	1
			−.005	9904	0–	90492	1	1259.857	10	79374.09	.005	31821	3–	111196	4
1241.116	2b	80572.65	.007	29245	3–	109818	4	1259.995	5	79365.39	.009	13992	2–	93358	3
1241.147	100	80570.63	.004	19632	5–	100202	4	1260.119	20	79357.58	.002	41322	5–	120680	5
1241.382	100	80555.38	.006	30507	4–	111063	3	1260.464	1b	79335.86	.005	12427	2–	91763	3
1241.501	200b	80547.66	−.001	36226	3–	116774	3	1260.580	5	79328.56	.002	36467	4–	115795	3
1241.534	50b	80545.52	.002	2256	1–	82801	2	1260.649	20	79324.22	.005	17380	4–	96704	4
1241.580	20	80542.53	.003	24490	2–	105033	3	1260.861	10	79310.88	.003	30507	4–	109818	4
1241.903	5	80521.59	.009	14899	3–	95422	3	1261.484	1	79271.71	.004	28977	4–	108249	3
1242.100	20	80508.82	.002	36226	3–	116735	4	1261.566	50	79266.56	.002	36353	2–	115619	1
1242.174	20	80504.02	.005	16723	5–	97227	5	1261.849	50	79248.78	.007	6277	3–	85527	2
1242.590	50	80477.07	.001	45388	2–	125865	3	1261.969	20	79241.25	.001	31821	3–	111063	3
1242.643	50	80473.64	.000	37769	4–	118243	3	1262.090	20	79233.65	.004	13992	2–	93226	2
1242.934	3b	80454.79	.003	12427	2–	92882	1	1262.303	50	79220.28	.002	28391	3–	107611	2
1243.464	20	80420.50	.006	16723	5–	97144	4	1262.494	10	79208.30	.006	12881	1–	92089	0
			.004	36353	2–	116774	3	1262.531	20	79205.98	.004	28062	2–	107269	3
1243.880	5	80393.61	.004	36226	3–	116620	2	1263.092	50	79170.80	.006	22212	3–	101383	3
			.003	10968	1–	91362	2	1263.351	10b	79154.57	.002	18376	4–	97531	3
1244.038	100	80383.40	.009	15038	3–	95422	3	1263.420	10	79150.24	.004	4461	2–	83611	3
1244.540	5	80350.97	.002	19851	3–	100202	4	1263.936	20	79117.93	.002	20696	2–	99814	2
1244.640	1	80344.52	.007	12881	1–	93226	2	1264.176	5b	79102.91	.003	22955	2–	102058	1
1245.079	5	80316.19	.003	16723	5–	97039	6	1264.344	1	79092.40	.003	12881	1–	91973	2
1245.227	100	80306.64	.002	36467	4–	116774	3	1265.184	50	79039.89	.005	26588	4–	105628	4
1245.326	20	80300.26	.005	6277	3–	86578	3	1265.233	20	79036.83	.006	2256	1–	81293	2
1245.785	3	80270.67	.008	24490	2–	104761	2	1265.428	10	79024.65	.004	31838	1–	110863	2
1245.832	50	80267.64	.007	36467	4–	116735	4	1265.758	20	79004.04	.004	37769	4–	116774	3
											.001	29245	3–	108249	3
1246.345	100	80234.61	.003	14899	3–	95134	2								
1246.387	10	80231.90	.003	29512	0–	109744	1	1266.248	5	78973.47	.004	27726	0–	106699	1
1247.099	5b	80186.10	.006	28062	2–	108249	3	1266.375	10b	78965.55	.001	37769	4–	116735	4
1247.411	50	80166.04	.004	13700	4–	93867	4	1266.873	200	78934.51	−.001	10968	1–	89903	1
1247.655	50	80150.36	.005	17380	4–	97531	3	1267.071	50	78922.18	−.001	39321	3–	118243	3
1248.018	50	80127.05	.002	39321	3–	119448	2	1267.455	1	78898.26	.003	39344	2–	118243	3
1248.388	50b	80103.30	.003	39344	2–	119448	2	1267.561	200	78891.67	.001	7686	4–	86578	3
1248.497	100b	80096.31	.004	15038	3–	95134	2	1267.779	1	78878.10	.000	28391	3–	107269	3
1248.787	5	80077.71	.004	33631	4–	113709	3	1267.882	100	78871.69	.000	33972	2–	112844	2
			.000	13700	4–	93778	5	1267.969	1	78866.28	.001	23080	1–	101947	2
								1268.017	1	78863.30	.001	28977	4–	107840	4
1249.910	3	80005.76	.003	6277	3–	86283	3								
1249.987	20	80000.83	.003	12881	1–	92882	1	1268.290	100	78846.32	.006	18380	6–	97227	5
1250.091	10	79994.18	−.007	6277	3–	86271	4	1268.568	3	78829.04	.003	15038	3–	93867	4
1250.248	100	79984.13	.004	31211	5–	111196	4	1269.033	20	78800.16	.005	6277	3–	85078	4
1250.289	100	79981.51	.003	16723	5–	96704	4	1269.070	50	78797.86	.004	2256	1–	81054	1
1250.408	100	79973.90	.002	41322	5–	121296	4	1269.557	50b	78767.63	.002	18376	4–	97144	4
1250.888	100b	79943.21	.001	29800	2–	109744	1	1269.580	50b	78766.21	.003	6277	3–	85044	2
1250.941	10	79939.82	.003	16621	2–	96561	2	1270.203	50	78727.57	.001	20696	2–	99424	3
1251.508	50	79903.60	.006	7686	4–	87590	4	1270.905	100	78684.09	.000	47181	4–	125865	3
1252.400	20	79846.69	.008	17380	4–	97227	5	1271.318	3	78658.53	.003	18380	6–	97039	6
								1271.662	10	78637.25	.002	24490	2–	103127	3
1252.523	5	79838.85	.006	2256	1–	82095	1								
1252.646	200	79831.01	.000	36904	5–	116735	4	1272.156	200	78606.71	−.002	31211	5–	109818	4
1253.180	20	79797.00	.004	24490	2–	104287	2	1272.336	20b	78595.59	−.001	29245	3–	107840	4
1253.308	50	79788.85	.004	31821	3–	111610	3	1272.408	5	78591.14	.001	6277	3–	84869	4
1253.347	50	79786.36	.003	13992	2–	93778	1	1272.513	50	78584.66	.003	7686	4–	86271	4
1253.600	20	79770.26	.003	20432	4–	100202	4	1273.580	20	78518.82	.003	4461	2–	82980	2
1253.708	5	79763.39	.006	17380	4–	97144	4	1273.670	100	78513,27	.004	16621	2–	95134	2
1253.789	5	79758.24	.005	33972	2–	113730	1	1274.189	5	78481.29	.000	12881	1–	91362	2
1254.126	10	79736.80	.004	33972	2–	113709	3	1274.563	20	78458.26	.000	14899	3–	93358	3
1255.249	20	79665.47	.006	4461	2–	84127	3	1274.723	5	78448.42	.002	29800	2–	108249	3
								1275.053	3	78428.11	.002	22955	2–	101383	3
1275.222	100	78417.72	−.004	27252	3–	105670	2	1293.315	300b	77320.68	.000	15038	3–	92358	2
1275.287	100b	78413.72	.003	16723	5–	95137	5	1293.630	3	77301.86	.003	43378	4–	120680	5
1275.901	100	78375.99	.003	27252	3–	105628	4	1293.773	50	77293.31	−.001	6277	3–	83571	4
1276.056	10	78366.47	.002	13992	2–	92358	2	1294.014	100	77278.92	.000	28391	3–	105670	2
			−.004	29245	3–	107611	2	1294.071	10	77275.51	−.002	39344	2–	116620	2
1276.287	50	78352.28	.009	22955	2–	101307	1	1294.704	50	77237.73	−.002	28391	3–	105628	4
1276.476	200b	78340.68	−.001	4461	2–	82801	2	1295.129	50b	77212.39	−.004	22212	3–	99424	3
1276.676	20	78328.41	.002	18376	4–	96704	4	1295.157	50b	77210.72	.000	24097	1–	101307	1
1276.711	50	78326.26	−.001	14899	3–	93226	2	1295.535	50	77188.19	−.004	29512	0–	106699	1
1276.818	50b	78319.70	.005	15038	3–	93358	3	1295.634	20	77182.29	.001	7686	4–	84869	4
1276.897	100	78314.85	.000	10968	1–	89283	1	1296.046	100	77157.76	−.003	16621	2–	93778	1
1277.338	50	78287.81	.001	47577	2–	125865	3	1296.232	3	77146.68	.003	16723	5–	93870	6
1277.600	100	78271.76	.001	10968	1–	89240	2	1296.279	10	77143.89	.002	16723	5–	93867	4
1277.893	100	78253.81	−.001	18376	4–	96630	3	1296.545	300b	77128.06	.000	17380	4–	94508	3
1278.325	50	78227.37	.006	25963	6–	104191	5	1297.170	100	77090.90	−.003	33972	2–	111063	3
			−.005	23080	1–	101307	1	1297.475	200	77072.78	.002	19632	5–	96704	4
1278.623	5	78209.14	.001	19851	3–	98061	2	1297.561	200	77067.67	−.002	28977	4–	106044	3
1278.962	50	78188.41	−.007	15038	3–	93226	2	1297.927	200	77045.94	−.006	18376	4–	95422	3
1279.622	20	78148.08	.003	7686	4–	85834	3	1298.105	20	77035.37	−.001	27252	3–	104287	2
1279.939	1	78128.72	−.002	31821	3–	109950	3	1298.330	20	77022.02	−.001	12881	1–	89903	1
1280.648	1	78085.47	.002	24490	2–	102576	2	1299.201	1	76970.38	.000	28062	2–	105033	3
1280.815	20	78075.29	.000	13700	4–	91776	4	1299.376	200	76960.02	.000	2256	1–	79216	1
1280.979	200	78065.29	−.001	12427	2–	90492	1	1299.787	100	76935.68	−.002	15038	3–	91973	2
1281.368	100	78041.59	.000	17380	4–	95422	3	1299.972	100	76924.73	−.002	35429	6–	112353	6
1281.620	5	78026.25	−.001	37769	4–	115795	3	1300.343	100	76902.79	.001	12427	2–	89329	3
1281.656	20	78024.06	−.003	29245	3–	107269	3	1300.410	50	76898.82	.005	29800	2–	106699	1
1282.009	10	78002.57	.000	31838	1–	109841	2	1300.538	100	76891.26	−.002	33972	2–	110863	2
1282.103	20	77996.85	−.004	31821	3–	109818	4	1300.640	50	76885.23	−.001	23317	5–	100202	4
1282.360	300b	77981.22	.004	13992	2–	91973	2	1300.791	100	76876.30	.002	14899	3–	91776	4
1282.690	50	77961.16	−.003	24097	1–	102058	1	1301.075	20	76859.52	.002	22955	2–	99814	2
1283.397	100	77918.21	.002	43378	4–	121296	4	1301.137	300	76855.86	.008	12427	2–	89283	1
1283.711	300	77899.15	−.003	10968	1–	88867	0	1301.182	100	76853.20	−.002	19851	3–	96704	4
1283.904	1	77887.44	−.001	16621	2–	94508	3	1301.334	100	76844.22	.000	29855	1–	106699	1
1284.396	200	77857.61	−.001	24490	2–	102348	3	1301.412	3	76839.62	−.003	41403	2–	118243	3
1284.533	100	77849.30	−.001	6277	3–	84127	3	1301.537	1	76832.24	.005	42615	3–	119448	2
1285.160	50	77811.32	.003	27252	3–	105064	4				.000	4461	2–	81293	2
			−.005	37808	1–	115619	1	1301.858	20	76813.29	.000	12427	2–	89240	2
1285.664	300	77780.82	−.001	27252	3–	105033	3	1302.087	20	76799.78	−.002	29245	3–	106044	3
1285.822	50	77771.26	−.002	13992	2–	91763	3	1302.452	20	76778.26	.000	19851	3–	96630	3
1286.060	100	77756.87	−.002	17380	4–	95137	5	1302.731	200	76761.82	.001	30507	4–	107269	3
1286.304	200	77742.12	.000	30507	4–	108249	3	1302.827	100	76756.16	.001	18380	6–	95137	5
1287.345	50	77679.25	.000	19851	3–	97531	3	1303.133	200b	76738.14	.001	15038	3–	91776	4
1287.497	100	77670.08	.000	12427	2–	90097	3	1303.205	50	76733.90	.000	23080	1–	99814	2
1287.765	100	77653.92	−.001	28391	3–	106044	3	1303.621	100	76709.41	−.003	19851	3–	96561	2
1288.021	10	77638.49	.000	33972	2–	111610	3	1303.801	10	76698.82	.000	28062	2–	104761	2
1288.095	10	77634.03	.004	4461	2–	82095	1	1304.240	1	76673.00	−.001	28391	3–	105064	4
1288.466	5	77611.67	−.006	12881	1–	90492	1	1304.610	20b	76651.26	.000	28977	4–	105628	4
1288.615	50	77602.70	.005	26588	4–	104191	5	1304.985	100	76629.23	−.003	31211	5–	107840	4
			−.001	22212	3–	99814	2	1305.182	1	76617.67	−.001	36226	3–	112844	2
1288.733	200	77595.59	−.001	19632	5–	97227	5	1305.397	50	76605.05	−.002	16621	2–	93226	2
1289.197	5	77567.66	−.002	24490	2–	102058	1	1306.518	50	76539.32	.002	26588	4–	103127	3
1289.246	50	77564.72	.000	33631	4–	111196	4	1306.672	200b	76530.30	−.001	2256	1–	78786	2
1289.717	50	77536.39	−.002	7686	4–	85222	5	1306.779	100	76524.03	.000	6277	3–	82801	2
1290.121	100	77512.11	.000	19632	5–	97144	4	1307.187	100	76500.15	.001	13992	2–	90492	1
1290.167	5	77509.35	−.002	27252	3–	104761	2	1307.345	200	76490.90	−.003	36353	2–	112844	2
1290.464	50	77491.51	.000	20696	2–	98188	1	1307.412	100	76486.98	−.002	17380	4–	93867	4
1290.603	20	77483.16	−.004	36226	3–	113709	3	1307.618	100	76474.93	−.004	39321	3–	115795	3
1290.720	300b	77476.14	−.004	12427	2–	89903	1	1307.827	200	76462.71	−.003	14899	3–	91362	2
1290.850	20	77468.33	.000	29800	2–	107269	3	1308.024	50	76451.20	−.003	39344	2–	115795	3
1291.003	200	77459.15	−.004	14899	3–	92358	2	1308.212	200	76440.21	.002	7686	4–	84127	3
1291.108	100	77452.85	−.001	39321	3–	116774	3	1308.425	10	76427.77	−.002	31821	3–	108249	3
1291.464	20	77431.50	.002	33631	4–	111063	3	1308.476	3	76424.79	−.001	29245	3–	105670	2
1291.862	300	77407.65	−.002	19632	5–	97039	6	1308.859	10	76402.42	−.001	12881	1–	89283	1
1292.131	100	77391.53	.001	7686	4–	85078	4	1308.966	100	76396.18	.001	13700	4–	90097	3
1292.365	10	77377.52	.001	36353	2–	113730	1	1309.143	100	76385.85	.003	12427	2–	88813	3
1292.486	20	77370.28	−.001	13992	2–	91362	2	1309.185	10	76383.40	.001	29245	3–	105628	4
1292.588	1	77364.17	.005	20696	2–	98061	2	1309.273	200	76378.26	−.003	0	0–	76378	1
1292.722	20	77356.15	−.001	36353	2–	113709	3	1309.403	200	76370.68	.000	28391	3–	104761	2
1293.096	200b	77333.78	.002	6277	3–	83611	3	1309.595	20	76359.49	−.002	12881	1–	89240	2
			−.004	30507	4–	107840	4	1310.199	300	76324.28	.000	15038	3–	91362	2
1310.288	200	76319.10	−.003	33631	4–	109950	3	1332.178	20	75065.04	.000	28062	2–	103127	3
1311.046	20	76274.97	.001	39344	2–	115619	1	1332.280	200	75059.30	−.002	15038	3–	90097	3
1311.089	100	76272.47	−.001	20432	4–	96704	4	1332.393	10	75052.93	.001	16723	5–	91776	4
1311.125	20	76270.38	.000	7686	4–	83957	5	1332.436	5	75050.51	−.003	41570	1–	116620	2
1311.576	100	76244.15	−.001	29800	2–	106044	3	1332.577	20	75042.57	.000	29245	3–	104287	2
1311.913	100b	76224.57	.006	28062	2–	104287	2	1332.705	10	75035.36	−.004	37808	1–	112844	2
			−.005	6277	3–	82502	4	1333.520	100	74989.50	−.001	20432	4–	95422	3
1312.377	20b	76197.62	.000	20432	4–	96630	3	1333.658	200b	74981.74	−.001	18376	4–	93358	3
1312.560	100b	76186.99	−.001	33631	4–	109818	4	1334.029	20	74960.89	.000	29800	2–	104761	2
1313.506	200	76132.12	−.001	18376	4–	94508	3	1335.008	10	74905.92	.002	29855	1–	104761	2
1314.106	10	76097.36	.001	4461	2–	80558	3	1335.774	10b	74862.96	.000	51002	3–	125865	3
1314.292	50b	76086.59	.000	28977	4–	105064	4	1335.802	3b	74861.39	.000	31838	1–	106699	1
1314.822	20	76055.92	−.001	28977	4–	105033	3	1336.244	50	74836.63	-.001	36226	3–	111063	3
1315.457	20	76019.21	−.003	31821	3–	107840	4	1336.525	50	74820.90	.001	13992	2–	88813	3
1315.740	50	76002.86	.000	12427	2–	88429	3	1336.625	10	74815.30	.002	7686	4–	82502	4
1315.820	20	75998.24	.000	18376	4–	94374	4	1336.889	50	74800.53	.002	14899	3–	89700	4
1316.020	50	75986.69	−.003	12881	1–	88867	0	1336.993	10	74794.71	.000	26588	4–	101383	3
1316.177	200	75977.62	.007	33972	2–	109950	3	1337.704	100	74754.95	.002	4461	2–	79216	1
			.001	17380	4–	93358	3	1337.925	300b	74742.61	.000	19632	5–	94374	4
1316.941	50	75933.55	.000	20696	2–	96630	3	1338.167	100b	74729.09	−.002	13700	4–	88429	3
											−.004	36467	4–	111196	4
1317.007	1	75929.74	.005	13700	4–	89630	5								
1317.138	5	75922.19	−.004	37808	1–	113730	1	1338.512	50	74709.83	−.001	36353	2–	111063	3
1317.335	50	75910.84	.001	13992	2–	89903	1	1338.780	200	74694.87	−.004	27252	3–	101947	2
1317.537	10	75899.20	−.004	10968	1–	86867	2	1339.289	50	74666.48	.001	20696	2–	95363	1
1317.583	10	75896.55	.004	28391	3–	104287	2	1339.360	50	74662.53	−.003	15038	3–	89700	4
			−.001	10968	1–	86865	1	1339.465	3	74656.67	−.001	19851	3–	94508	3
1317.794	100b	75884.39	−.001	7686	4–	83571	4	1339.819	1	74636.95	.001	36226	3–	110863	2
1317.947	10	75875.59	−.002	27252	3–	103127	3	1340.156	100	74618.18	−.004	33631	4–	108249	3
1318.060	20	75869.08	.001	29800	2–	105670	2	1340.558	20	74595.80	−.001	36467	4–	111063	3
			−.005	33972	2–	109841	2	1340.921	100b	74575.61	.008	22955	2–	97531	3
											−.008	2256	1–	76831	2
1318.137	20	75864.65	−.003	20696	2–	96561	2								
1318.663	5	75834.39	−.004	20696	2–	96530	1	1341.228	50	74558.54	−.001	10968	1–	85527	2
1319.011	100	75814.38	−.002	29855	1–	105670	2	1341.257	10	74556.93	−.003	30507	4–	105064	4
1319.438	10b	75789.84	−.002	31821	3–	107611	2	1341.814	5b	74525.98	.001	30507	4–	105033	3
1319.470	5b	75788.01	.000	29245	3–	105033	3	1341.847	100	74524.14	.000	7686	4–	82210	5
1319.735	5	75772.79	.002	31838	1–	107611	2	1342.040	10	74513.43	−.003	28062	2–	102576	2
1319.966	20	75759.53	.001	26588	4–	102348	3	1342.095	20	74510.37	−.004	36353	2–	110863	2
1320.350	3	75737.49	.002	16621	2–	92358	2	1342.408	3	74493.00	−.003	22212	3–	96704	4
1320.706	3	75717.08	.008	24097	1–	99814	2	1342.514	20	74487.12	−.002	29800	2–	104287	2
1322.268	100	75627.63	−.003	42615	3–	118243	3	1342.550	5	74485.12	−.003	9904	0–	84389	1
								1343.355	20	74440.49	−.001	12427	2–	86867	2
1323.207	100	75573.97	.000	27252	3–	102826	4								
1324.174	3	75518.78	−.004	10968	1–	86487	2	1343.396	20	74438.21	−.004	20696	2–	95134	2–
1324.215	5	75516.44	.001	29245	3–	104761	2				−.005	12427	2–	86865	1
1324.413	300b	75505.15	−.001	19632	5–	95137	5				−.007	13992	2–	88429	3
1324.487	3b	75500.93	−.002	2256	1–	77757	2	1343.506	20	74432.12	.000	29855	1–	104287	2
1324.696	200	75489.02	.004	18380	6–	93870	6	1343.542	50	74430.13	.000	14899	3–	89329	3
1325.395	300b	75449.21	.001	36904	5–	112353	6	1343.765	10b	74417.77	.005	22212	3–	96630	3
1326.030	100	75413.08	−.004	41322	5–	116735	4	1344.160	200	74395.91	−.002	17380	4–	91776	4
1326.220	5	75402.27	.000	18376	4–	93778	5	1344.224	20	74392.36	−.007	41403	2–	115795	3
1326.303	10	75397.55	.004	18380	6–	93778	5	1344.296	10	74388.38	−.004	39321	3–	113709	3
								1344.338	10	74386.05	−.002	39344	2–	113730	1
1326.357	20	75394.48	.003	16723	5–	92117	4								
1326.540	5	75384.08	.005	36226	3–	111610	3	1344.396	100	74382.85	.000	17380	4–	91763	3
1326.784	1	75370.22	−.002	41403	2–	116774	3	1344.728	20	74364.48	.000	39344	2–	113709	3
1327.096	100	75352.50	.001	16621	2–	91973	2	1345.007	20	74349.06	−.001	22212	3–	96561	2
1327.560	100b	75326.16	−.001	50539	2–	125865	3	1345.163	100b	74340.43	.003	14899	3–	89240	2
1327.603	200b	75323.72	.007	24490	2–	99814	2	1345.439	200	74325.18	.002	4461	2–	78786	2
			−.001	27252	3–	102576	2	1346.041	100	74291.94	−.001	15038	3–	89329	3
1327.685	5	75319.07	−.001	22212	3–	97531	3				−.003	36904	5–	111196	4
1328.169	1	75291.62	−.006	13992	2–	89283	1	1346.243	5	74280.79	.000	6277	3–	80558	3
1328.772	20	75257.46	.001	36353	2–	111610	3	1346.310	3	74277.10	−.001	33972	2–	108249	3
								1347.020	50b	74237.95	.002	19632	5–	93870	6
1329.201	200	75233.17	−.001	22955	2–	98188	1								
1329.499	200	75216.30	.001	41403	2–	116620	2	1347.069	100	74235.25	−.001	19632	5–	93867	4
1329.539	20	75214.04	.001	28977	4–	104191	5	1347.287	40	74223.24	.000	31821	3–	106044	3
1329.889	50b	75194.25	−.002	12427	2–	87621	2	1347.533	80	74209.69	−.006	33631	4–	107840	4
1330.787	50	75143.51	.001	36467	4–	111610	3	1347.666	3	74202.36	.000	15038	3–	89240	2
1331,176	50	75121.55	−.002	30507	4–	105628	4	1348.413	10	74161.25	−.003	13700	4–	87862	5
1331.340	20b	75112.29	−.003	13700	4–	88813	3	1348.468	3	74158.23	−.002	42615	3–	116774	3
1331.430	100b	75107.22	.002	23080	1–	98188	1	1348.602	80	74150.86	.008	12427	2–	86578	3
1331.448	100b	75106.20	−.003	22955	2–	98061	2				−.006	28977	4–	103127	3
1331.634	5	75095.71	−.002	27252	3–	102348	3	1348.679	100	74146.63	.000	19632	5–	93778	5
								1348.967	1	74130.80	−.001	27252	3–	101383	3
1349.128	5b	74121.95	−.001	2256	1–	76378	1	1363.754	20	73327.01	.002	29800	2–	103127	3
1349.173	10	74119.48	−.001	42615	3–	116735	4	1363.888	100b	73319.80	.002	6277	3–	79597	4
1349.254	30	74115.03	−.001	47181	4–	121296	4	1364.304	80b	73297.45	.004	52568	3–	125865	3
1349.968	100	74075.83	.002	20432	4–	94508	3	1364.336	100b	73295.73	.002	4461	2–	77757	2
			−.003	10968	1–	85044	2	1364.383	50	73293.20	.001	37769	4–	111063	3
1350.254	40	74060.14	−.002	12427	2–	86487	2	1364.592	40	73281.98	−.001	16621	2–	89903	1
			−.004	45388	2–	119448	2	1365.333	10	73242.21	.002	31821	3–	105064	4
1350.434	1	74050.27	−.005	41570	1–	115619	1	1365.933	80	73210.03	−.002	22212	3–	95422	3
1351.068	40	74015.52	−.003	19851	3–	93867	4	1366.492	30	73180.08	−.002	42615	3–	115795	3
1351.272	10b	74004.35	.001	42615	3–	116620	2	1367.933	10	73103.00	−.003	29245	3–	102348	3
1351.595	50	73986.66	−.003	12881	1–	86867	2	1367.989	100	73100.00	−.002	12427	2–	85527	2
1351.643	100	73984.03	.000	12881	1–	86865	1	1368.324	30	73082.11	−.001	20696	2–	93778	1
1352.010	1	73963.95	.000	24097	1–	98061	2	1368.833	lb	73054.93	−.008	37808	1–	110863	2
1352.131	80	73957.33	−.005	28391	3–	102348	3	1369.104	30	73040.47	.001	24490	2–	97531	3
1352.406	30	73942.29	−.004	20432	4–	94374	4	1370.012	200	72992.06	.002	28391	3–	101383	3
1352.933	80	73913.49	−.003	14899	3–	88813	3	1370.242	400b	72979.81	−.001	31211	5–	104191	5
1352.997	100	73909.99	−.003	23317	5–	97227	5	1370.993	100	72939.83	.003	31821	3–	104761	2
1353.364	80	73889.95	−.004	13700	4–	87590	4	1371.314	30	72922.76	.008	31838	1–	104761	2
1353.705	20	73871.34	−.002	16621	2–	90492	1				−.004	22212	3–	95134	2
1353.971	40	73856.83	−.003	12427	2–	86283	3	1371.480	60	72913.93	.001	36904	5–	109818	4
1354.049	200	73852.57	−.005	31211	5–	105064	4	1371.603	50	72907.39	.007	16723	5–	89630	5
1354.110	50	73849.24	−.003	28977	4–	102826	4	1372.170	40	72877.27	.004	13700	4–	86578	3
1354.253	80b	73841.45	−.007	37769	4–	111610	3	1372.209	50	72875.20	.003	13992	2–	86867	2
1354.883	50	73807.11	−.003	31821	3–	105628	4	1372.265	100b	72872.22	−.005	7686	4–	80558	3
1354.929	80	73804.61	.000	0	0–	73804	1	1372.460	30	72861.87	.002	6277	3–	79139	3
1355.353	80	73781.52	.001	17380	4–	91161	5	1373.486	100	72807.44	−.004	7686	4–	80493	5
1355.468	100	73775.26	−.004	15038	3–	88813	3	1373.898	80	72785.61	−.001	18376	4–	91161	5
1355.739	10b	73760.51	−.001	29512	0–	103272	1	1373.985	30	72781.00	.001	18380	6–	91161	5
1356.086	80	73741.64	−.003	18376	4–	92117	4	1374.091	50	72775.38	−.001	29800	2–	102576	2
1356.408	80	73724.13	−.004	36226	3–	109950	3	1374.995	10	72727.54	.002	33972	2–	106699	1
1356.450	100	73721.85	−.001	23317	5–	97039	6	1375.110	50	72721.46	−.001	14899	3–	87621	2
1356.896	50	73697.62	.001	9904	0–	83601	1	1375.196	80	72716.91	−.002	17380	4–	90097	3
			−.001	24490	2–	98188	1	1375.345	80	72709.03	−.003	16621	2–	89329	3
1357.144	30	73684.15	.002	30507	4–	104191	5	1376.226	80	72662.48	−.003	16621	2–	89283	1
1357.307	80b	73675.30	−.003	22955	2–	96630	3	1376.537	100	72646.07	−.002	12881	1–	85527	2
1357.998	30	73637.81	−.001	33631	4–	107269	3	1376.777	300	72633.40	.000	10968	1–	83601	1
1358.158	10	73629.14	−.001	13992	2–	87621	2	1377.017	200b	72620.74	−.001	30507	4–	103127	3
1358.423	100	73614.77	−.002	36226	3–	109841	2	1377.086	200	72617.11	.000	12427	2–	85044	2
			−.007	26588	4–	100202	4	1377.208	100	72610.67	−.003	4461	2–	77071	3
1358.582	100	73606.16	−.002	12881	1–	86487	2	1377.732	200	72583.06	.002	15038	3–	87621	2
			−.002	22955	2–	96561	2				−.005	13700	4–	86283	3
1358.843	80	73592.02	−.002	36226	3–	109818	4	1377.968	100	72570.63	−.001	13700	4–	86271	4
1359.034	3b	73581.68	−.001	27726	0–	101307	1	1378.120	50	72562.62	−.009	27252	3–	99814	2
1359.141	40	73575.88	−.003	22955	2–	96530	1	1378.309	10	72552.67	.000	15038	3–	87590	4
1359.239	1	73570.58	−.002	24490	2–	98061	2	1378.419	50b	72546.88	.007	29800	2–	102348	3
1359.507	10	73556.08	.000	28391	3–	101947	2	1378.630	200	72535.78	−.001	2256	1–	74791	2
1359.986	100	73530.17	−.001	14899	3–	88429	3	1378.748	30	72529.57	−.003	20696	2–	93226	2
1360.121	100b	73522.87	−.002	39321	3–	112844	2	1379.143	300	72508.80	−.003	6277	3–	78786	2
1360.431	50	73506.12	.002	19851	3–	93358	3	1379.398	60	72495.39	−.008	13992	2–	86487	2
1360.567	3	73498.77	.006	39344	2–	112844	2	1379.950	50	72466.39	−.006	31821	3–	104287	2
			−.003	47181	4–	120680	5	1380.269	1	72449.65	−.007	31838	1–	104287	2
1360.773	100	73487.64	.003	36353	2–	109841	2	1380.575	3b	72433.59	.001	24097	1–	96530	1
1360.855	30	73483.21	−.002	36467	4–	109950	3	1380.731	30	72425.40	.002	0	0–	72425	1
1360.911	80	73480.19	.001	23080	1–	96561	2	1380.874	100	72417.90	−.004	43378	4–	115795	3
1360.981	1b	73476.41	−.005	16621	2–	90097	3	1380.953	50	72413.76	−.004	33631	4–	106044	3
1361.069	1	73471.66	−.001	29800	2–	103272	1	1381.057	50	72408.31	−.003	22955	2–	95363	1
1361.466	30	73450.24	−.005	23080	1–	96530	1	1381.104	300	72405.84	.000	28977	4–	101383	3
1361.756	80	73434.59	.002	20432	4–	93867	4	1381.781	200	72370.37	−.003	4461	2–	76831	2
1361.905	20	73426.56	−.004	37769	4–	111196	4	1382.609	30	72327.03	−.002	41403	2–	113730	1
1361.993	300	73421.82	.002	6277	3–	79699	3	1382.742	300	72320.07	−.001	17380	4–	89700	4
1362.091	80	73416.53	.003	29855	1–	103272	1	1383.189	1	72296.70	−.006	22212	3–	94508	3
1362.254	50b	73407.75	.002	12427	2–	85834	3	1383.283	5	72291.79	−.003	13992	2–	86283	3
1362.402	200	73399.77	.001	18376	4–	91776	4	1383.328	20	72289.44	.003	39321	3–	111610	3
1362.550	200	73391.80	.002	15038	3–	88429	3	1383.462	50	72282.43	−.002	23080	1–	95363	1
1362.637	300	73387.12	.000	23317	5–	96704	4	1383.777	30	72265.98	.001	19851	3–	92117	4
			−.005	18376	4–	91763	3				−.002	39344	2–	111610	3
1362.875	20	73374.30	−.003	19851	3–	93226	2	1383.945	10	72257.21	.001	29800	2–	102058	1
1362.940	80	73370.80	−.002	28977	4–	102348	3	1384.072	300	72250.58	.000	17380	4–	89630	5
1363.194	50	73357.13	.000	43378	4–	116735	4	1384.993	50	72202.53	−.003	29855	1–	102058	1
1363.305	10	73351.16	−.001	36467	4–	109818	4	1385.194	80	72192.05	−.001	16621	2–	88813	3
1363.403	1	73345.89	.005	20432	4–	93778	5	1385.429	200	72179.81	−.004	22955	2–	95134	2
1385.580	1	72171.94	−.004	27252	3–	99424	3	1403.507	80	71250.09	.000	18380	6–	89630	5
1385.778	80	72161.63	.002	16723	5–	88884	6	1403.591	80	71245.83	−.002	15038	3–	86283	3
1386.068	80	72146.53	−.004	29800	2–	101947	2	1403.833	40	71233.54	−.001	15038	3–	86271	4
1386.111	80	72144.29	−.002	19632	5–	91776	4	1403.989	200	71225.63	−.002	28977	4–	100202	4
1386.199	10	72139.71	−.003	24490	2–	96630	3	1404.987	30	71175.04	−.003	12427	2–	83601	1
1386.304	100	72134.25	−.005	13700	4–	85834	3	1405.117	20	71168.45	−.008	13700	4–	84869	4
1386.373	100	72130.66	−.002	6277	3–	78408	4	1405.480	100	71150.07	−.001	9904	0–	81054	1
			−.005	18380	6–	90511	7	1405.561	20	71145.97	.000	22212	3–	93358	3
1386.541	80	72121.92	−.003	19851	3–	91973	2	1405.705	20	71138.68	.002	16723	5–	87862	5
1387.125	20	72091.56	−.002	29855	1–	101947	2	1405.935	400b	71127.04	−.003	10968	1–	82095	1
1387.527	80	72070.67	−.003	24490	2–	96561	2	1406.944	15	71076.03	−.004	25963	6–	97039	6
1387.848	100	72054.00	−.004	23080	1–	95134	2	1407.238	50	71061.19	−.004	33972	2–	105033	3
1387.954	20b	72048.50	.002	37769	4–	109818	4	1407.323	50	71056.89	−.001	23317	5–	94374	4
1388.639	600b	72012.96	.002	7686	4–	79699	3	1407.419	50	71052.05	.000	13992	2–	85044	2
1388.941	100	71997.30	.000	33631	4–	105628	4	1407.468	40	71049.57	−.001	17380	4–	88429	3
1389.191	20	71984.34	−.002	46259	2–	118243	3	1407.603	5	71042.76	−.001	36226	3–	107269	3
1389.513	100	71967.66	.001	14899	3–	86867	2	1407.716	30b	71037.06	.008	24097	1–	95134	2
1389.617	80	71962.27	−.002	12427	2–	84389	1	1407.787	100	71033.47	−.006	28391	3–	99424	3
1389.865	40	71949.43	.002	17380	4–	89329	3	1407.834	50	71031.10	.000	41322	5–	112353	6
1390.143	1	71935.05	.003	37808	1–	109744	1	1408.175	5	71013.90	.001	22212	3–	93226	2
1390.496	3	71916.78	.002	4461	2–	76378	1	1408.355	50	71004.82	−.001	31821	3–	102826	4
1390.611	400b	71910.84	.004	7686	4–	79597	4	1408.446	50	71000.24	−.002	16621	2–	87621	2
1390.898	10	71896.00	.002	36353	2–	108249	3	1409.374	50	70953.49	.001	18376	4–	89329	3
1391.302	200	71875.12	−.005	39321	3–	111196	4	1409.587	200	70942.77	−.004	26588	4–	97531	3
1392.111	50	71833.35	−.001	10968	1–	82801	2	1410.914	20	70876.04	−.001	30507	4–	101383	3
1392.187	5	71829.43	.001	15038	3–	86867	2	1410.981	100	70872.68	−.002	24490	2–	95363	1
1392.382	100	71819.37	−.001	23317	5–	95137	5	1411.086	300	70867.40	.001	16723	5–	87590	4
1392.525	50b	71812.00	−.002	28391	3–	100202	4	1411.954	80	70823.84	−.004	22955	2–	93778	1
1392.588	50	71808.75	.002	16621	2–	88429	3	1412.260	3	70808.49	.001	27252	3–	98061	2
1392.843	30	71795.60	.005	29512	0–	101307	1	1412.388	50	70802.07	−.004	36467	4–	107269	3
1393.104	50	71782.15	−.001	36467	4–	108249	3	1412.489	200	70797.01	−.002	15038	3–	85834	3
1393.695	50b	71751.71	.001	28062	2–	99814	2	1412.548	100	70794.05	−.003	6277	3–	77071	3
1393.886	20	71741.88	−.002	39321	3–	111063	3	1412.639	50	70789.49	−.001	33972	2–	104761	2
1394.295	100b	71720.83	−.001	18376	4–	90097	3	1413.674	20	70737.67	−.001	31838	1–	102576	2
1394.703	30	71699.85	.002	12427	2–	84127	3	1414.006	80	70721.06	−.003	12881	1–	83601	1
1394.989	20	71685.15	.004	20432	4–	92117	4	1414.468	20	70697.96	−.002	23080	1–	93778	1
1395.115	40b	71678.68	−.001	14899	3–	86578	3	1415.116	1	70665.59	.003	20696	2–	91362	2
1395.221	50	71673.23	.003	4461	2–	76134	3				.000	47577	2–	118243	3
1395.575	20	71655.05	.002	22212	3–	93867	4	1415.546	20	70644.12	−.002	24490	2–	95134	2
1396.370	40	71614.26	.002	36226	3–	107840	4	1415.645	1	70639.18	−.002	26588	4–	97227	5
1396.896	80	71587.29	.000	14899	3–	86487	2	1415.847	50b	70629.10	.000	39321	3–	109950	3
1396.993	20	71582.32	.002	29800	2–	101383	3	1415.883	200	70627.30	−.002	14899	3–	85527	2
1397.069	30	71578.43	.004	4461	2–	76039	2	1417.231	100	70560.13	−.001	33631	4–	104191	5
1397.659	100	71548.21	.004	2256	1–	73804	1	1417.358	500	70553.81	−.005	6277	3–	76831	2
1397.811	100	71540.43	-.001	15038	3–	86578	3	1417.439	15	70549.77	−.006	23317	5–	93867	4
1397.924	200	71534.65	.004	13992	2–	85527	2	1417.905	100	70526.59	−.004	31821	3–	102348	3
1398.171	50	71522.01	.000	13700	4–	85222	5	1418.036	50	70520.07	−.004	39321	3–	109841	2
1398.240	5	71518.48	.001	39344	2–	110863	2	1418.356	250	70504.16	−.002	18380	6–	88884	6
1398.398	20	71510.40	.002	19851	3–	91362	2	1418.499	50	70497.05	.001	39321	3–	109818	4
1398.443	30	71508.10	.003	12881	1–	84389	1	1418.658	100	70489.15	−.004	15038	3–	85527	2
1399.008	500	71479.22	−.001	6277	3–	77757	2	1418.807	50	70481.75	−.003	17380	4–	87862	5
1399.518	400	71453.17	−.001	7686	4–	79139	3	1419.506	50	70447.04	−.005	28977	4–	99424	3
			−.003	47995	1–	119448	2	1419.716	100	70436.62	.002	18376	4–	88813	3
1399.600	30	71448.99	.001	15038	3–	86487	2	1419.924	200	70426.30	−.005	13700	4–	84127	3
1399.776	20	71440.00	.002	41403	2–	112844	2	1420.465	40	70399.48	−.006	39344	2–	109744	1
1399.916	300b	71432.86	−.003	17380	4–	88813	3	1420.509	10	70397.30	−.004	13992	2–	84389	1
			−.005	33631	4–	105064	4	1420.961	150	70374.91	−.004	12427	2–	82801	2
1400.096	50	71423.67	−.001	28391	3–	99814	2	1421.530	50	70346.74	−.002	36353	2–	106699	1
1400.876	500	71383.91	.001	14899	3–	86283	3	1421.850	200	70330.91	.005	43378	4–	113709	3
1401.008	5	71377.18	.003	13700	4–	85078	4				−.003	4461	2–	74791	2
1401.113	30b	71371.83	−.002	14899	3–	86271	4	1421.969	100	70325.02	−.003	10968	1–	81293	2
1401.664	20b	71343.77	−.002	20432	4–	91776	4	1422.448	50	70301.34	−.005	55564	4–	125865	3
1401.923	20	71330.59	.003	20432	4–	91763	3	1423.060	50	70271.11	−.002	22955	2–	93226	2
1402.054	50	71323.93	.002	18376	4–	89700	4	1423.363	30	70256.15	−.001	13700	4–	83957	5
1402.398	80	71306.43	−.004	31821	3–	103127	3	1423.558	100	70246.52	−.002	16621	2–	86867	2
1402.973	20	71277.21	−.004	20696	2–	91973	2	1423.605	15	70244.20	−.005	16621	2–	86865	1
1403.034	3	71274.11	−.001	41570	1–	112844	2	1423.928	15	70228.27	−.003	42615	3–	112844	2
1403.232	100	71264.05	−.005	25963	6–	97227	5	1424.102	100	70219.69	−.003	31838	1–	102058	1
1403.348	50	71258.16	.000	36353	2–	107611	2	1424.291	100	70210.37	−.002	17380	4–	87590	4
1403.418	10	71254.61	.000	18376	4–	89630	5	1424.937	100	70178.54	.009	29245	3–	99424	3
											−.001	14899	3–	85078	4
1425.123	100	70169.38	−.001	2256	1–	72425	1	1449.766	200	68976.65	.003	0	0–	68976	1
1425.584	5	70146.69	−.001	22212	3–	92358	2	1450.090	100	68961.24	.000	19851	3–	88813	3
1425.629	10	70144.48	−.002	14899	3–	85044	2	1451.020	5	68917.04	−.001	30507	4–	99424	3
1425.700	150	70140.98	.000	7686	4–	77827	4	1451.193	5	68908.82	.001	50539	2–	119448	2
1426.014	10	70125.54	−.002	31821	3–	101947	2	1451.255	50	68905.88	.001	16621	2–	85527	2
			−.007	28062	2–	98188	1	1451.310	15	68903.27	.002	17380	4–	86283	3
1426.197	100	70116.54	−.003	26588	4–	96704	4	1451.532	50	68892.73	.001	23080	1–	91973	2
1426.548	20	70099.29	−.003	12881	1–	82980	2	1451.567	50	68891.07	.001	17380	4–	86271	4
1427.122	50	70071.09	−.001	37769	4–	107840	4				−.001	37808	1–	106699	1
1427.175	200	70068.49	−.002	19632	5–	89700	4	1452.088	150	68866.35	.000	12427	2–	81293	2
1427.477	100	70053.67	−.003	18376	4–	88429	3	1452.781	100	68833.50	−.001	26588	4–	95422	3
1427.747	150	70040.42	−.004	15038	3–	85078	4	1453.156	30	68815.74	.001	29245	3–	98061	2
1428.205	10	70017.96	−.002	24490	2–	94508	3	1453.314	50	68808.25	−.001	22955	2–	91763	3
1428.444	100	70006.24	−.002	15038	3–	85044	2	1453.391	5	68804.61	−.001	27726	0–	96530	1
1428.590	250	69999.09	−.003	19632	5–	89630	5	1453.474	300	68800.68	.010	13700	4–	82502	4
1429.440	50	69957.47	−.003	16621	2–	86578	3	1453.809	50	68784.83	.002	24097	1–	92882	1
1430.059	40	69927.18	−.003	22955	2–	92882	1	1454.852	150	68735.51	−.001	24490	2–	93226	2
1430.391	300	69910.95	−.004	13700	4–	83611	3	1455.088	150	68724.37	.000	36904	5–	105628	4
1430.494	35	69905.92	−.002	22212	3–	92117	4	1455.354	150	68711.80	.002	14899	3–	83611	3
1430.783	50	69891.80	−.004	27252	3–	97144	4	1456.214	50	68671.23	.000	14899	3–	83571	4
1431.154	100	69873.68	−.001	41322	5–	111196	4	1457.147	15	68627.26	.000	12427	2–	81054	1
1431.227	40	69870.12	−.001	13700	4–	83571	4	1457.645	50	68603.81	.001	33972	2–	102576	2
1431.310	100	69866.07	−.001	16621	2–	86487	2	1458.192	35	68578.07	.000	19851	3–	88429	3
1431.499	150	69856.84	−.001	6277	3–	76134	3	1458.287	100	68573.61	.001	15038	3–	83611	3
1432.028	100	69831.04	−.001	15038	3–	84869	4	1458.424	15	68567.17	.001	28062	2–	96630	3
1432.282	100	69818.65	−.003	36226	3–	106044	3	1458.712	5	68553.63	.001	28977	4–	97531	3
1432.632	200	69801.60	−.007	23080	1–	92882	1	1458.819	30	68548.60	.000	26588	4–	95137	5
1433.444	300	69762.06	−.001	6277	3–	76039	2	1458.922	100	68543.76	.000	20696	2–	89240	2
1434.807	100	69695.78	−.002	30507	4–	100202	4	1459.151	150	68533.00	.000	15038	3–	83571	4
1435.098	40	69681.65	−.002	24097	1–	93778	1	1459.554	50	68514.08	.001	6277	3–	74791	2
1435.368	150	69668.54	−.004	12427	2–	82095	1	1459.643	100	68509.90	.000	13700	4–	82210	5
1435.488	30	69662.72	−.001	16621	2–	86283	3	1459.726	150	68506.01	.001	2256	1–	70762	2
1436.298	100	69623.43	−.001	29800	2–	99424	3	1459.855	500	68499.95	−.005	16723	5–	85222	5
1436.378	100	69619.56	.000	13992	2–	83611	3	1459.940	30	68495.97	−.003	41322	5–	109818	4
1436.578	50	69609.86	−.001	13992	2–	83601	1	1460.537	50	68467.97	−.003	28062	2–	96530	1
1437.241	100	69577.75	−.002	36467	4–	106044	3	1460.742	100	68458.36	.001	23317	5–	91776	4
1437.576	35	69561.54	−.001	31821	3–	101383	3	1460.825	40	68454.47	.002	17380	4–	85834	3
1437.787	50	69551.33	−.003	22212	3–	91763	3	1460.967	50	68447.82	.002	7686	4–	76134	3
1437.849	100	69548.33	−.001	16723	5–	86271	4	1461.009	50	68445.85	−.003	51002	3–	119448	2
1438.087	20	69536.82	−.001	46259	2–	115795	3	1461.499	5	68422.90	.004	16621	2–	85044	2
1438.859	35	69499.51	−.003	37769	4–	107269	3	1461.557	100	68420.18	.003	4461	2–	72881	3
1439.147	35	69485.61	.000	18376	4–	87862	5	1461.722	200	68412.46	−.001	12881	1–	81293	2
1439.239	250	69481.16	−.001	18380	6–	87862	5	1461.826	250	68407.59	−.006	22955	2–	91362	2
1439.300	15	69478.22	−.003	19851	3–	89329	3	1462.170	5	68391.50	.000	24490	2–	92882	1
1439.484	10	69469.34	−.001	31838	1–	101307	1	1462.264	5	68387.10	.004	29800	2–	98188	1
1439.835	100	69452.40	.000	27252	3–	96704	4	1462.397	200	68380.88	.007	31821	3–	100202	4
1440.017	20	69443.62	−.001	36226	3–	105670	2				−.007	20432	4–	88813	3
1440.908	45	69400.68	−.002	20696	2–	90097	3	1462.504	5	68375.88	−.002	33972	2–	102348	3
1441.160	200	69388.55	−.001	19851	3–	89240	2	1462.941	50	68355.46	−.009	16723	5–	85078	4
1442.100	50	69343.32	.002	4461	2–	73804	1	1463.225	10	68342.19	.004	45388	2–	113730	1
1442.654	15	69316.69	.000	36353	2–	105670	2	1463.270	5	68340.09	.001	41403	2–	109744	1
1442.752	150	69311.98	−.001	9904	0–	79216	1	1463.679	15	68320.99	−.003	45388	2–	113709	3
1442.822	50	69308.62	−.001	27252	3–	96561	2	1463.831	30	68313.90	−.001	28391	3–	96704	4
1442.999	100	69300.12	.000	33972	2–	103272	1	1464.433	25	68285.81	.000	29245	3–	97531	3
1443.135	20	69293.59	−.003	41570	1–	110863	2	1464.525	40	68281.52	.000	23080	1–	91362	2
1443.462	50	69277.89	.000	23080	1–	92358	2	1464.846	35	68266.56	.000	39344	2–	107611	2
1443.671	50	69267.86	.001	20432	4–	89700	4	1464.981	50	68260.27	−.001	29800	2–	98061	2
1444.332	200	69236.16	.000	4461	2–	73697	3	1465.630	250	68230.04	−.002	19632	5–	87862	5
1444.787	50	69214.35	.001	12881	1–	82095	1	1466.160	50	68205.38	−.001	29855	1–	98061	2
			−.001	18376	4–	87590	4	1466.850	50	68173.30	.000	12881	1–	81054	1
1444.952	30	69206.45	−.001	20696	2–	89903	1	1466.926	50	68169.76	.005	28391	3–	96561	2
1445.122	200	69198.31	.003	20432	4–	89630	5				−.006	27252	3–	95422	3
			−.006	17380	4–	86578	3	1466.989	100	68166.84	−.003	28977	4–	97144	4
1445.187	15	69195.20	−.002	33631	4–	102826	4	1467.142	35	68159.73	−.001	36904	5–	105064	4
1446.015	20	69155.58	.001	33972	2–	103127	3	1467.436	350	68146.07	−.007	16723	5–	84869	4
1446.338	45	69140.13	−.003	28391	3–	97531	3	1467.748	250	68131.59	−.001	12427	2–	80558	3
1446.575	40	69128.80	.002	24097	1–	93226	2	1468.076	50	68116.36	.002	20696	2–	88813	3
1447.409	30	69088.97	.000	15038	3–	84127	3	1468.132	400	68113.77	−.005	7686	4–	75800	5
1449.089	10	69008.87	.000	23080	1–	92089	0	1468.358	100	68103.28	.000	13992	2–	82095	1
1449.527	100	68988.02	.001	13992	2–	82980	2	1468.852	100	68080.38	.000	14899	3–	82980	2
1469.262	50	68061.38	.000	36226	3–	104287	2	1492.185	300	67015.82	−.004	19851	3–	86867	2
1470.641	250	67997.56	−.003	20432	4–	88429	3	1492.962	150	66980.94	−.001	16621	2–	83601	1
1470.744	50	67992.80	.003	31821	3–	99814	2	1493.695	50	66948.07	−.002	22955	2–	89903	1
			−.005	24097	1–	92089	0	1494.219	100	66924.59	.000	20696	2–	87621	2
1471.129	50	67975.00	−.004	33972	2–	101947	2	1494.342	50	66919.09	.001	36353	2–	103272	1
1471.355	400	67964.56	−.005	4461	2–	72425	1	1495.399	15	66871.78	.000	24490	2–	91362	2
1471.841	40	67942.12	.001	15038	3–	82980	2	1495.937	50	66847.73	.001	16723	5–	83571	4
1472.227	20	67924.31	−.002	39344	2–	107269	3	1496.061	350	66842.19	−.003	18380	6–	85222	5
1472.613	500	67906.50	−.004	25963	6–	93870	6	1496.349	50	66829.33	−.001	29800	2–	96630	3
1472.707	50	67902.17	−.003	14899	3–	82801	2	1496508	30	66822.23	−.001	23080	1–	89903	1
1472.775	40	67899.03	−.004	29245	3–	97144	4	1497.264	100	66788.49	−.001	10968	1–	77757	2
1472.858	50	67895.21	−.002	18376	4–	86271	4	1497.896	45	66760.31	−.002	29800	2–	96561	2
1473.076	50	67885.16	−.001	22212	3–	90097	3	1498.273	10	66743.51	.000	28391	3–	95134	2
1473.142	100	67882.12	−.001	27252	3–	95134	2	1498.576	15	66730.02	−.002	29800	2–	96530	1
1473.265	45	67876.45	.000	24097	1–	91973	2	1498.655	10	66726.50	.001	19851	3–	86578	3
1473.444	50	67868.21	−.002	24490	2–	92358	2	1498.721	20	66723.56	.003	39321	3–	106044	3
1473.598	50	67861.11	−.001	37808	1–	105670	2	1498.785	350	66720.71	−.002	2256	1–	68976	1
1473.643	40	67859.04	−.002	37769	4–	105628	4				−.008	30507	4–	97227	5
1473.968	100	67844.08	.002	23317	5–	91161	5	1498.965	100	66712.70	.000	12427	2–	79139	3
1474.541	100	67817.71	.005	10968	1–	78786	2	1499.132	5	66705.27	.002	29855	1–	96561	2
			.004	43378	4–	111196	4								
								1499.209	25	66701.84	.001	18376	4–	85078	4
1474.593	500	67815.32	−.009	25963	6–	93778	5	1499.248	50	66700.11	−.002	39344	2–	106044	3
1475.229	100	67786.09	.000	26588	4–	94374	4	1499.813	150	66674.98	.001	29855	1–	96530	1
1475.609	150	67768.63	−.010	16621	2–	84389	1	1500.132	45	66660.80	−.002	36467	4–	103127	3
1475.714	50	67763.81	−.001	15038	3–	82801	2	1500.614	100	66639.39	.002	19632	5–	86271	4
1475.974	25	67751.87	−.001	33631	4–	101383	3	1500.667	200	66637.04	−.003	30507	4–	97144	4
1476155	200	67743.56	−.001	6277	3–	74021	4	1501.170	100	66614.71	.000	27252	3–	93867	4
1476378	50	67733.33	.000	20696	2–	88429	3	1501.416	450	66603.79	−.002	6277	3–	72881	3
1476.508	5	67727.37	.003	28977	4–	96704	4	1501.477	150	66601.09	−.002	22212	3–	88813	3
1477.024	45	67703.71	.002	50539	2–	118243	3	1501.897	300	66582.46	−.002	2256	1–	68838	0
1477.151	50	67697.89	.000	17380	4–	85078	4								
								1502.031	30	66576.52	.002	17380	4–	83957	5
1477.434	40	67684.92	−.002	43378	4–	111063	3	1502.130	45	66572.13	.000	43378	4–	109950	3
1478.138	5	67652.68	.000	28977	4–	96630	3	1502.258	5	66566.46	.000	13992	2–	80558	3
1479.237	100	67602.42	.001	31821	3–	99424	3	1503.130	50	66527.85	−.002	47181	4–	113709	3
			−.003	14899	3–	82502	4	1503.343	5	66518.42	−.003	41322	5–	107840	4
1479.622	500	67584.83	−.003	18380	6–	85965	6	1503.921	500	66492.85	−.006	18376	4–	84869	4
1480.622	10	67539.18	−.001	10968	1–	78507	0	1504.238	30	66478.84	.002	37808	1–	104287	2
1480.662	50	67537.36	−.001	22955	2–	90492	1	1504.352	150	66473.80	.000	9904	0–	76378	1
1481.351	45	67505.95	.000	16621	2–	84127	3	1505.008	150	66444.83	−.006	28977	4–	95422	3
1481.736	150	67488.41	.004	17380	4–	84869	4	1505.115	5	66440.11	.000	43378	4–	109818	4
			−.001	22212	3–	89700	4								
1482.270	50	67464.09	−.001	15038	3–	82502	4	1505.302	150	66431.85	.001	19851	3–	86283	3
1482.456	25	67455.63	−.003	45388	2–	112844	2	1505.531	10	66421.75	.000	37769	4–	104191	5
1483.030	50	67429.52	−.001	20432	4–	87862	5	1505.578	10	66419.67	−.001	19851	3–	86271	4
1483.247	350	67419.65	−.003	6277	3–	73697	3	1506.134	35	66395.15	.001	24097	1–	90492	1
1483.432	150	67411.25	.006	23080	1–	90492	1	1506.167	25	66393.70	−.002	14899	3–	81293	2
			−.007	33972	2–	101383	3	1506.414	300	66382.81	−.004	23317	5–	89700	4
1484.011	100	67384.95	−.002	29245	3–	96630	3	1506.592	50	66374.97	−.001	22955	2–	89329	3
1484.583	250	67358.98	.001	28062	2–	95422	3	1506.946	50	66359.38	.000	12427	2–	78786	2
			−.002	0	0–	67358	1				−.004	36467	4–	102826	4
1485.119	150	67334.67	−.005	42615	3–	109950	3				−.006	16621	2–	82980	2
1485.532	150	67315.95	−.003	29245	3–	96561	2	1507.175	200	66349.30	.006	31838	1–	98188	1
1485.878	50	67300.28	−.001	28062	2–	95363	1	1507.504	500	66334.82	.009	12881	1–	79216	1
1486.009	35	67294.34	−.002	37769	4–	105064	4				−.003	7686	4–	74021	4
1486.166	50	67287.23	−.002	36904	5–	104191	5	1507.725	5	66325.09	.000	39344	2–	105670	2
1486.354	200	67278.72	.000	26588	4–	93867	4	1508.127	50	66307.41	.001	39321	3–	105628	4
1486.487	15	67272.70	−.001	24490	2–	91763	3	1508.268	500	66301.21	−.003	4461	2–	70762	2
			−.001	12427	2–	79699	3	1508.628	50	66285.39	−.001	22955	2–	89240	2
1486.652	45	67265.24	−.001	24097	1–	91362	2	1509.675	50	66239.42	−.003	31821	3–	98061	2
1486857	50	67255.96	−.001	27252	3–	94508	3	1510.065	4	66222.31	.004	31838	1–	98061	2
1487.199	50	67240.50	.004	51002	3–	118243	3				−.001	45388	2–	111610	3
1487.344	200	67233.94	−.003	16723	5–	83957	5	1510.110	50	66220.34	.002	4461	2–	70681	3
1488.315	50	67190.08	.001	26588	4–	93778	5	1510.164	50	66217.97	−.003	22212	3–	88429	3
1489.018	250	67158.36	−.004	20432	4–	87590	4	1510.513	20	66202.67	−.002	23080	1–	89283	1
1489.376	50	67142.21	−.001	22955	2–	90097	3	1510.626	100	66197.72	−.001	30507	4–	96704	4
1489,823	50	67122.07	.001	27252	3–	94374	4	1510,788	35	66190.62	.000	17380	4–	83571	4
1489.914	200	67117.97	−.002	22212	3–	89329	3	1511.013	5	66180.77	−.001	16621	2–	82801	2
1490.940	45	67071.78	−.003	28062	2–	95134	2	1511.102	25	66176.87	−.003	29245	3–	95422	3
1491.151	250	67062.29	−.002	13992	2–	81054	1	1511.293	200	66168.51	−.002	20696	2–	86865	1
1491.849	50	67030.91	.000	28391	3–	95422	3	1511.496	45	66159.62	.002	28977	4–	95137	5
1492.117	100	67018.87	−.003	29512	0–	96530	1				−.001	23080	1–	89240	2
1511.645	20	66153.10	.000	47577	2–	113730	1	1534.610	50	65163.14	−.001	28062	2–	93226	2
1511.808	150	66145.97	−.002	20432	4–	86578	3	1534.758	50	65156.85	−.001	36226	3–	101383	3
1512.136	15	66131.62	−.001	47577	2–	113709	3	1535.193	100	65138.39	−.002	20696	2–	85834	3
1512.335	150	66122.92	−.001	30507	4–	96630	3	1535.587	100	65121.68	.000	17380	4–	82502	4
1512.462	15	66117.36	.000	28391	3–	94508	3	1535.727	250	65115.74	−.002	55564	4–	120680	5
1513.302	20	66080.66	.002	50539	2–	116620	2	1535.957	4	65105.99	.004	27252	3–	92358	2
1513.945	45	66052.60	−.003	27726	0–	93778	1	1536.034	100	65102.73	.000	2256	1–	67358	1
1514.198	50	66041.56	−.002	41570	1–	107611	2	1536.212	450	65095.18	.006	2256	1–	67351	0
1514.907	50	66010.65	.000	7686	4–	73697	3	1536.777	100	65071.25	.000	10968	1–	76039	2
1515.270	100	65994.84	.000	36353	2–	102348	3	1537.118	5	65056.81	−.002	37769	4–	102826	4
1515.532	35	65983.43	.003	28391	3–	94374	4	1538.567	40	64995.54	.000	42615	3–	107611	2
			−.008	19851	3–	85834	3	1539.253	20	64966.58	.000	39321	3–	104287	2
1516.702	40	65932.53	−.003	31211	5–	97144	4	1539.537	10	64954.59	−.001	36353	2–	101307	1
1516.937	15	65922.32	−.004	36904	5–	102826	4	1539.814	10	64942.91	.000	39344	2–	104287	2
1517.325	5	65905.46	.000	12881	1 —	78786	2	1540.478	150	64914.92	−.004	30507	4–	95422	3
1517.524	20	65896.82	−.001	13700	4–	79597	4	1541.073	50	64889.85	−.001	28977	4–	93867	4
1517.696	50	65889.35	.000	29245	3–	95134	2	1541.231	25	64883.20	−.001	31821	3–	96704	4
1517.870	20	65881.79	.000	20696	2–	86578	3	1541.400	50	64876.09	−.003	12881	1–	77757	2
1518.415	50	65858.15	−.002	22955	2–	88813	3	1541.653	50	64865.44	−.002	27252	3–	92117	4
1518.575	50	65851.21	.000	20432	4–	86283	3	1541.892	250	64855.39	−.009	2256	1–	67111	2
			−.001	29512	0–	95363	1								
								1542.483	50	64830.54	−.003	17380	4–	82210	5
1518.777	50	65842.45	−.002	33972	2–	99814	2				−.003	20696	2–	85527	2
1518.856	10	65839.03	−.001	20432	4–	86271	4	1542.764	5	64818.73	.009	28062	2–	92882	1
1519.620	50	65805.93	−.001	24097	1–	89903	1	1543.116	45	64803.94	−.001	46259	2–	111063	3
1519.929	40	65792.55	.006	33631	4–	99424	3	1543.209	50	64800.04	−.002	14899	3–	79699	3
1520.068	20	65786.53	.004	23080	1–	88867	0	1543.375	100	64793.07	.004	51002	3–	115795	3
1520.241	450	65779.04	−.005	16723	5–	82502	4				−.005	24490	2–	89283	1
1521.319	250	65732.43	.005	51002	3–	116735	4	1545.055	50	64722.62	−.003	31838	1–	96561	2
1521.587	50	65720.86	−.003	36226	3–	101947	2	1545.415	100	64707.54	.001	29800	2–	94508	3
1521.896	10	65707.51	.002	13992	2–	79699	3				−.003	13700	4–	78408	4
1522.339	5	65688.39	.000	39344	2–	105033	3								
								1546.251	300	64672.55	−.005	16621	2–	81293	2
1522.645	150	65675.19	.003	52568	3–	118243	3	1546.403	10	64666.20	−.001	22955	2–	87621	2
			−.001	19851	3–	85527	2	1546.510	45	64661.72	.000	15038	3–	79699	3
1523.025	50	65658.80	.000	14899	3–	80558	3	1546.659	150	64655.49	−.002	22212	3–	86867	2
1523.613	10	65633.46	.000	42615	3–	108249	3	1546.709	30	64653.40	−.004	42615	3–	107269	3
1523.764	250	65626.96	−.007	12881	1–	78507	0	1546.914	100	64644.83	−.005	12427	2–	77071	3
1523.979	25	65617.70	−.002	51002	3–	116620	2	1547.004	25	64641.07	−.002	41403	2–	106044	3
1524.234	50	65606.72	−.002	24490	2–	90097	3	1547.461	15	64621.98	.000	29245	3–	93867	4
1524.320	150	65603.02	−.001	7686	4–	73289	5	1547.882	15	64604.41	−.003	46259	2–	110863	2
1524.530	10	65593.99	−.003	36353	2–	101947	2	1548.502	30	64578.54	−.005	37769	4–	102348	3
1524.840	25	65580.65	.000	18376	4–	83957	5								
								1549.321	250	64544.40	−.004	23317	5–	87862	5
1524.941	500	65576.31	−.004	18380	6–	83957	5	1549.818	50	64523.71	.000	27252	3–	91776	4
1525.155	100	65567.11	.001	23317	5–	88884	6	1550.009	250	64515.75	−.003	4461	2–	68976	1
1525.266	35	65562.33	−.001	29800	2–	95363	1	1550.128	10	64510.80	−.002	27252	3–	91763	3
1525.994	20	65531.06	−.001	28977	4–	94508	3	1550.754	350	64484.76	−.007	6277	3–	70762	2
1526.031	4	65529.47	−.003	26588	4–	92117	4	1551.914	50	64436.56	−.002	20432	4–	84869	4
1526.237	100	65520.62	−.001	15038	3–	80558	3	1552.090	30	64429.25	−.001	47181	4–	111610	3
1526.543	10	65507.49	−.002	29855	1–	95363	1	1552.695	450	64404.15	.004	12427	2–	76831	2
1527.008	20	65487.54	.001	16723	5–	82210	5				−.008	6277	3–	70681	3
1527.310	250	65474.59	.007	22955	2–	88429	3	1553.099	500	64387.40	−.008	7686	4–	72073	4
			−.006	16621	2–	82095	1								
								1553.259	50	64380.76	−.003	28977	4–	93358	3
1527.971	50	65446.27	−.001	19632	5–	85078	4	1553.608	250	64366.30	.001	22212	3–	86578	3
1528.143	50	65438.90	−.001	13700	4–	79139	3	1555.310	50	64295.86	−.003	28062	2–	92358	2
1528.661	15	65416.73	.002	39344	2–	104761	2	1555.446	150	64290.24	−.001	16723	5–	81013	6
1528.761	10	65412.45	.000	24490	2–	89903	1	1555.813	5	64275.08	.002	19851	3–	84127	3
1528.828	50	65409.58	−.001	10968	1–	76378	1				−.001	22212	3–	86487	2
1528.990	300	65402.65	−.006	20432	4–	85834	3	1556.014	45	64266.77	−.003	29512	0–	93778	1
1529.117	50	65397.22	−.001	28977	4–	94374	4	1556.661	50	64240.06	−.001	14899	3–	79139	3
1529.553	35	65378.58	.001	22212	3–	87590	4	1557.247	10	64215.89	−.002	33972	2–	98188	1
1530.184	10	65351.62	.001	46259	2–	111610	3	1557.484	50	64206.12	−.003	52568	3–	116774	3
1530.692	50	65329.93	.000	12427	2–	77757	2								
								1558.421	100	64167.51	−.004	52568	3–	116735	4
1530.867	15	65322.46	−.002	31821	3–	97144	4	1559.409	1000	64126.86	−.004	13700	4–	77827	4
1531.507	50	65295.16	−.001	28062	2–	93358	3	1559.749	50	64112.88	−.002	29245	3–	93358	3
1532.907	300	65235.53	−.006	18376	4–	83611	3	1561.049	50	64059.49	−.001	22212	3–	86271	4
1533.169	50	65224.38	−.007	13992	2–	79216	1	1561.226	45	64052.23	.001	52568	3–	116620	2
1533.781	350	65198.36	−.005	25963	6–	91161	5	1561.691	50	64033.15	−.003	47577	2–	111610	3
1533.864	250	65194.83	.000	7686	4–	72881	3	1562.144	5	64014.59	−.002	47181	4–	111196	4
			−.004	18376	4–	83571	4	1562.467	20	64001.35	−.004	30507	4–	94508	3
1533.921	150	65192.41	−.002	19851	3–	85044	2	1562.968	100	63980.84	−.002	29245	3–	93226	2
1534.059	200	65186.54	−.006	24097	1–	89283	1	1563.070	50	63976.66	−.004	36226	3–	100202	4
1534.335	50	65174.82	−.002	26588	4–	91763	3								
1563.710	200	63950.48	−.003	12881	1–	76831	2	1590.812	25	62860.98	−.003	45388	2–	108249	3
1563.874	500	63943.77	−.006	6277	3–	70221	4	1590.904	150	62857.34	−.004	14899	3–	77757	2
1564.023	50	63937.68	−.003	16621	2–	80558	3	1591.070	4	62850.79	.000	30507	4–	93358	3
1564.323	5	63925.42	−.001	31211	5–	95137	5	1591.222	100	62844.78	−.003	27252	3–	90097	3
1564.379	50	63923.13	−.005	29855	1–	93778	1	1591.359	100	62839.37	−.003	13992	2–	76831	2
1564.683	10	63910.71	−.001	28062	2–	91973	2	1591.539	150	62832.26	−.004	22212	3–	85044	2
1564.941	200	63900.17	.003	9904	0–	73804	1	1591.957	15	62815.77	−.003	56632	2–	119448	2
1565.268	150	63886.82	−.004	14899	3–	78786	2	1593.109	45	62770.34	.000	24097	1–	86867	2
1565.744	40	63867.40	−.001	30507	4–	94374	4	1593.168	150	62768.02	−.003	24097	1–	86865	1
1566.548	200	63834.62	−.005	18376	4–	82210	5	1593.210	100	62766.36	−.006	27726	0–	90492	1
1566.663	200	63829.94	.000	18380	6–	82210	5	1593.842	50	62741.48	−.002	26588	4–	89329	3
1567.233	15	63806.72	.000	39321	3–	103127	3	1594.222	500	62726.52	−.009	4461	2–	67187	3
1567.785	50	63784.26	−.001	23080	1–	86865	1	1594.419	45	62718.77	.005	15038	3–	77757	2
1568.115	300	63770.83	−.005	16723	5–	80493	5	1595.990	40	62657.03	−.003	22212	3–	84869	4
1568.259	100	63764.98	−.003	13992	2–	77757	2	1596.026	50	62655.62	−.003	31211	5–	93867	4
1568.380	200	63760.06	−.006	19851	3–	83611	3	1596.164	50	62650.20	.000	19851	3–	82502	4
1568.976	50	63735.84	−.004	36467	4–	100202	4				−.005	4461	2–	67111	2
1569.385	20	63719.23	−.001	19851	3–	83571	4	1596.220	250	62648.01	−.005	23317	5–	85965	6
1569.671	100	63707.62	−.003	12427	2–	76134	3	1596.504	30	62636.86	−.003	47181	4–	109818	4
1569.994	50	63694.51	.000	20432	4–	84127	3	1596.611	500	62632.66	−.003	18380	6–	81013	6
1570.036	50	63692.81	−.004	20696	2–	84389	1	1596.783	5	62625.92	.000	39321	3–	101947	2
1571.603	25	63629.30	.001	41403	2–	105033	3	1597.739	5	62588.45	.008	33972	2–	96561	2
1571.754	100	63623.19	.005	22955	2–	86578	3	1597.983	200	62578.89	−.003	19632	5–	82210	5
			−.008	22212	3–	85834	3	1598.157	4	62572.08	−.002	22955	2–	85527	2
1572.010	100	63612.83	−.003	12427	2–	76039	2	1598.513	50	62558.14	.007	33972	2–	96530	1
1573.382	100	63557.36	−.004	29800	2–	93358	3				−.007	29800	2–	92358	2
1574.007	15	63532.12	−.001	22955	2–	86487	2	1598.642	100	62553.09	−.006	31821	3–	94374	4
1574.199	150	63524.37	.005	31838	1–	95363	1	1599.109	400	62534.82	−.004	7686	4–	70221	4
1574.199	150	63524.37	.003	20432	4–	83957	5	1599.207	5	62530.99	−.001	29245	3–	91776	4
			−.008	24097	1–	87621	2	1599.454	200	62521.34	−.003	9904	0–	72425	1
1574.590	100	63508.60	.002	26588	4–	90097	3	1599.519	100	62518.79	−.002	16621	2–	79139	3
			−.001	14899	3–	78408	4	1599.921	50	62503.09	−.003	29855	1–	92358	2
1574.873	30	63497.18	−.003	12881	1–	76378	1	1601.331	150	62448.05	.001	42615	3–	105064	4
1575.164	50	63485.45	−.005	47577	2–	111063	3				−.004	27252	3–	89700	4
1575.760	45	63461.44	−.002	36353	2–	99814	2	1601.697	200	62433.78	−.003	13700	4–	76134	3
1576.527	150	63430.57	−.004	20696	2–	84127	3	1602.819	100	62390.08	−.003	24097	1–	86487	2
1576.661	45	63425.18	.000	29800	2–	93226	2	1602.919	200	62386.18	−.006	13992	2–	76378	1
1578.008	500	63371.03	−.006	13700	4–	77071	3	1603.266	5	62372.68	−.003	47577	2–	109950	3
1578.282	50	63360.03	−.001	30507	4–	93867	4	1604.636	50	62319.43	−.002	17380	4–	79699	3
1578.336	50	63357.87	−.002	41403	2–	104761	2	1605.017	50	62304.64	−.005	50539	2–	112844	2
1579.059	50	63328.86	−.003	22955	2–	86283	3	1605.557	100	62283.68	−.003	20696	2–	82980	2
1579.405	50	63314.98	−.001	22212	3–	85527	2	1606.083	25	62263.28	.001	47577	2–	109841	2
1579.797	50	63299.27	.003	28062	2–	91362	2	1606.412	50	62250.53	−.002	43378	4–	105628	4
1579.874	50	63296.19	−.004	31838	1–	95134	2	1606.840	5	62233.95	.000	29855	1–	92089	0
1582.494	5	63191.39	−.002	50539	2–	113730	1	1607.122	25	62223.03	−.002	45388	2–	107611	2
1582.821	150	63178.34	−.003	17380	4–	80558	3	1607.266	200	62217.45	−.003	17380	4–	79597	4
1583.032	30	63169.92	−.003	50539	2–	113709	3	1608.176	40	62182.25	−.001	18376	4–	80558	3
1583.208	50	63162.89	−.001	31211	5–	94374	4	1608.441	50	62172.00	−.002	14899	3–	77071	3
1583.309	100	63158.87	−.002	12881	1–	76039	2	1608.562	200	62167.33	−.001	10968	1–	73135	2
1583.768	30	63140.56	−.003	28977	4–	92117	4	1609.856	50	62117.36	.003	18376	4–	80493	5
											−.007	29245	3–	91362	2
1583.818	5	63138.57	−.001	20432	4–	83571	4								
1584.014	100	63130.76	−.003	24490	2–	87621	2	1609.969	200	62113.00	.000	18380	6–	80493	5
1584.074	5	63128.36	−.001	19851	3–	82980	2	1610.323	200	62099.34	−.002	13700	4–	75800	5
1584.487	30	63111.91	−.001	26588	4–	89700	4	1610.618	150	62087.97	−.004	24490	2–	86578	3
1585.319	100	63078.79	−.003	16621	2–	79699	3	1610.891	50	62077.45	−.001	27252	3–	89329	3
1585.449	5	63073.62	−.003	33631	4–	96704	4	1611.094	100	62069.62	−.002	20432	4–	82502	4
1585.514	30	63071.03	−.002	36353	2–	99424	3	1611.249	15	62063.65	−.003	57384	3–	119448	2
1585.939	15	63054.13	−.001	42615	3–	105670	2	1611.721	4	62045.48	.000	31821	3–	93867	4
1586.637	35	63026.39	−.002	29855	1–	92882	1	1612.021	400	62033.93	.008	28062	2–	90097	3
1587.331	50	62998.83	−.003	33631	4–	96630	3				−.006	15038	3–	77071	3
								1612.752	20	62005.81	−.002	37808	1–	99814	2
1587.424	300	62995.14	−.005	7686	4–	70681	3								
1588.383	15	62957.11	−.004	36467	4–	99424	3	1612.993	10	61996.55	−.001	24490	2–	86487	2
1588.466	50	62953.82	−.002	23317	5–	86271	4	1613.218	5	61987.90	−.001	27252	3–	89240	2
1589.288	200	62921.26	−.002	25963	6–	88884	6	1613.862	250	61963.17	.005	23080	1–	85044	2
1589.687	15	62905.47	−.003	20696	2–	83601	1				−.006	39344	2–	101307	1
1589.880	50	62897.83	−.003	4461	2–	67358	1	1615.117	100	61915.02	−.002	22212	3–	84127	3
1590.233	25	62883.87	.001	41403	2–	104287	2	1615.221	150	61911.03	−.003	12881	1–	74791	2
1590.329	150	62880.07	−.004	22955	2–	85834	3	1615.370	150	61905.32	−.004	23317	5–	85222	5
1590.468	400	62874.58	−.002	16723	5–	79597	4	1615.551	200	61898.39	−.004	25963	6–	87862	5
1590.606	20	62869.12	−.004	41322	5–	104191	5	1616.012	10	61880.73	−.003	45388	2–	107269	3
								1617.214	25	61834.74	.002	36353	2–	98188	1
											−.003	36226	3–	98061	2
1618.296	50	61793.39	−.001	15038	3–	76831	2	1654.242	50	60450.65	.000	16621	2–	77071	3
			−.006	24490	2–	86283	3	1654.338	150	60447.14	.003	17380	4–	77827	4
1619.180	300	61759.66	−.006	17380	4–	79139	3	1655.030	15	60421.87	.004	28391	3–	88813	3
1620.115	20	61724.01	.000	41403	2–	103127	3	1655.529	15	60403.65	.001	36226	3–	96630	3
1620.586	5	61706.07	.001	28391	3–	90097	3	1656.492	50	60368.54	.004	27252	3–	87621	2
1620.684	10	61702.34	.003	41570	1–	103272	1	1656.533	50	60367.04	-.001	28062	2–	88429	3
1621.131	300	61685.33	−.006	16723	5–	78408	4	1656.792	100	60357.61	.003	20696	2–	81054	1
1621.941	4	61654.52	−.002	37769	4–	99424	3	1657.724	15	60323.67	.006	50539	2–	110863	2
1623.095	20	61610.69	−.002	30507	4–	92117	4	1657.820	35	60320.18	.006	13700	4–	74021	4
			−.002	56632	2–	118243	3	1658.479	50	60296.21	.004	29800	2–	90097	3
			−.003	35429	6–	97039	6				-.002	31821	3–	92117	4
1624.392	15	61561.50	.000	29800	2–	91362	2	1658.654	100	60289.85	.004	22212	3–	82502	4
1624.661	100	61551.30	−.002	23317	5–	84869	4	1659.622	100	60254.68	.003	12881	1–	73135	2
1625.055	50	61536.38	−.002	31821	3–	93358	3	1659.696	5	60252.00	.004	37808	1–	98061	2
1625.859	100	61505.95	−.007	33631	4–	95137	5	1660.035	15	60239.69	.002	36904	5–	97144	4
1627.155	50	61456.96	.000	10968	1–	72425	1	1660.146	25	60235.67	.004	33631	4–	93867	4
1627.346	10	61449.75	−.002	33972	2–	95422	3	1660.268	4	60231.24	.003	55564	4–	115795	3
1627.751	10	61434.46	−.006	22955	2–	84389	1	1660.846	50	60210.28	.008	42615	3–	102826	4
1628.681	30	61399.38	.006	22212	3–	83611	3				.001	16621	2–	76831	2
1628.999	15	61387.39	.002	31838	1–	93226	2	1661.248	500	60195.71	.004	0	0–	60195	1
1629.160	35	61381.33	.002	19632	5–	81013	6	1662.754	20	60141.19	.002	56632	2–	116774	3
1629.262	100	61377.48	.000	12427	2–	73804	1	1662.922	35	60135.11	.003	36904	5–	97039	6
1630.703	100	61323.25	.003	18376	4–	79699	3				-.002	31838	1–	91973	2
1631.095	10	61308.51	−.002	23080	1–	84389	1	1663.177	30	60125.89	.005	20432	4–	80558	3
1633.060	10	61234.74	.002	14899	3–	76134	3	1663.815	20	60102.84	.004	39321	3–	99424	3
1634.591	45	61177.38	.001	28062	2–	89240	2	1664.241	30	60087.45	.002	47181	4–	107269	3
			.001	27252	3–	88429	3	1664.323	25	60084.49	.005	29245	3–	89329	3
1634.733	500	61172.07	.004	41403	2–	102576	2	1664.470	30	60079.18	.003	39344	2–	99424	3
			−.002	22955	2–	84127	3	1664.965	250	60061.32	.001	20432	4–	80493	5
1635.588	250	61140.09	−.002	14899	3–	76039	2	1665.411	450	60045.24	.001	7686	4–	67731	5
1636.131	50	61119.80	−.001	28977	4–	90097	3	1665.592	25	60038.71	.004	28391	3–	88429	3
1636.545	50	61104.34	.002	16723	5–	77827	4	1665.782	250	60031.86	.002	18376	4–	78408	4
1637.432	35	61071.24	.000	50539	2–	111610	3	1665.972	100	60025.02	.004	22955	2–	82980	2
1638.596	40	61027.86	.003	17380	4–	78408	4	1666.620	50	60001.68	.004	25963	6–	85965	6
1639.288	350	61002.09	.002	26588	4–	87590	4	1666.806	50	59994.98	.003	29245	3–	89240	2
			−.009	15038	3–	76039	2	1667.624	200	59965.56	.003	19632	5–	79597	4
1639.870	20	60980.44	−.003	29512	0–	90492	1	1668.058	20	59949.95	.007	31211	5–	91161	5
1640.846	50	60944.17	.000	41403	2–	102348	3	1669.343	30	59903.81	.002	41403	2–	101307	1
1641.402	40	60923.53	.001	12881	1–	73804	1	1669.473	25	59899.14	.006	23080	1–	82980	2
1641.559	20	60917.70	.000	36226	3–	97144	4	1669.669	300	59892.11	.001	14899	3–	74791	2
1641.781	30	60909.46	.003	6277	3–	67187	3	1669.790	5	59887.77	.005	56732	1–	116620	2
1641.871	30	60906.12	.000	31211	5–	92117	4	1670.515	5	59861.78	.006	20696	2–	80558	3
1642.532	5	60881.61	.002	39321	3–	100202	4	1670.909	10	59847.66	.006	19851	3–	79699	3
1642.658	10	60876.94	.002	33631	4–	94508	3	1670.934	15	59846.77	.001	22955	2–	82801	2
1643.065	250	60861.86	−.001	19632	5–	80493	5	1671.251	5	59835.42	.007	28977	4–	88813	3
1643.154	25	60858.57	−.002	57384	3–	118243	3	1671.895	250	59812.37	.002	13992	2–	73804	1
1643.335	10	60851.87	.001	29245	3–	90097	3	1672.066	5	59806.25	.002	33972	2–	93778	1
1643.837	50	60833.28	.003	6277	3–	67111	2	1672.420	250	59793.59	.004	10968	1–	70762	2
1644.379	50	60813.23	.001	43378	4–	104191	5	1673.043	25	59771.33	.002	29512	0–	89283	1
1644.746	10	60799.66	.004	13992	2–	74791	2	1673.326	10	59761.22	.003	37769	4–	97531	3
1645.729	40	60763.35	.002	18376	4–	79139	3	1673.448	25	59756.86	.004	16621	2–	76378	1
1645.812	50	60760.28	.001	36467	4–	97227	5	1673.535	5	59753.75	.004	15038	3–	74791	2
1646.278	45	60743.08	.003	33631	4–	94374	4	1673.758	5	59745.79	.002	19851	3–	79597	4
1647.210	200	60708.71	.001	12427	2–	73135	2	1673.984	20	59737.73	.003	41570	1–	101307	1
1647.264	45	60706.72	.001	19851	3–	80558	3	1674.144	15	59732.02	.005	42615	3–	102348	3
1647.679	50	60691.43	.001	29800	2–	90492	1	1674.299	50	59726.49	.004	33631	4–	93358	3
1648.077	4	60676.78	.002	36467	4–	97144	4	1674.455	100	59720.92	.002	23080	1–	82801	2
1648.557	20	60659.11	.002	47181	4–	107840	4	1674.816	100	59708.05	.002	35429	6–	95137	5
1648.628	10	60656.50	.004	22955	2–	83611	3	1675.289	35	59691.19	.004	47577	2–	107269	3
			−.002	45388	2–	106044	3				.004	17380	4–	77071	3
1648.695	30	60654.03	.003	41403	2–	102058	1	1675.524	40	59682.82	.004	26588	4–	86271	4
1648.891	10	60646.82	.003	22955	2–	83601	1	1676.829	150	59636.37	.002	24490	2–	84127	3
1649.943	100	60608.15	−.001	51002	3–	111610	3	1677.402	30	59616.00	.001	47995	1–	107611	2
1650.255	35	60596.70	.003	20696	2–	81293	2	1677.432	15	59614.94	.001	27252	3–	86867	2
1650.446	25	60589.68	.002	22212	3–	82801	2	1678.175	35	59588.54	.005	13700	4–	73289	5
1651.104	500	60565.54	.003	2256	1–	62821	2	1678.761	35	59567.74	.002	29245	3–	88813	3
1651.909	50	60536.02	.003	33972	2–	94508	3	1679.030	50	59558.20	.001	28062	2–	87621	2
1652.252	35	60523.46	.000	50539	2–	111063	3	1680.292	200	59513.47	.002	16621	2–	76134	3
1652.346	15	60520.01	.003	31838	1–	92358	2	1680.540	15	59504.68	.004	24097	1–	83601	1
1653.484	20	60478.36	.003	36226	3–	96704	4	1680.657	150	59500.54	.004	7686	4–	67187	3
1654.140	200	60454.38	.001	12427	2–	72881	3	1681.172	25	59482.31	.007	29800	2–	89283	1
1681.867	25	59457.73	.003	37769	4–	97227	5	1713.806	50	58349.66	.000	35429	6–	93778	5
1682.019	50	59452.36	.004	28977	4–	88429	3	1713.930	20	58345.44	−.001	29245	3–	87590	4
1682.051	50	59451.23	.000	18376	4–	77827	4	1714.137	40	58338.39	.000	22955	2–	81293	2
1682.387	40	59439.36	.005	29800	2–	89240	2	1714.230	400	58335.23	−.002	12427	2–	70762	2
1683.810	10	59389.12	.001	57384	3–	116774	3	1714.934	50	58311.28	−.001	24490	2–	82801	2
1683.936	100	59384.68	−.001	29855	1–	89240	2	1715.401	20	58295.41	.000	52568	3–	110863	2
1684.228	35	59374.38	.000	37769	4–	97144	4	1715.798	5	58281.92	.002	36226	3–	94508	3
1684.262	20	59373.19	.001	45388	2–	104761	2	1715.841	45	58280.46	.000	26588	4–	84869	4
1684.904	40	59350.56	−.002	57384	3–	116735	4	1716.015	45	58274.55	−.002	27252	3–	85527	2
1685.610	20	59325.70	.005	27252	3–	86578	3	1716.602	500	58254.62	−.002	12427	2–	70681	3
1687.508	10	59258.98	.006	25963	6–	85222	5	1717.244	50	58232.84	−.002	36904	5–	95137	5
1687.664	4	59253.50	.005	33972	2–	93226	2	1717.840	30	58212.64	−.001	23080	1–	81293	2
1688.205	25	59234.51	.002	27252	3–	86487	2	1719.074	50	58170.85	.001	16621	2–	74791	2
1689.317	15	59195.52	.001	36226	3–	95422	3	1719.745	50	58148.16	.000	36226	3–	94374	4
1689.639	50	59184.24	.001	23317	5–	82502	4	1719.845	30	58144.77	.001	55564	4–	113709	3
1690.240	10	59163.20	−.001	56632	2–	115795	3				.000	33631	4–	91776	4
1690.805	25	59143.43	.008	13992	2–	73135	2	1723.890	400	58008.34	−.002	10968	1–	68976	1
1690.939	40	59138.74	.003	27726	0–	86865	1	1723.986	35	58005.11	−.003	43378	4–	101383	3
1691.430	450	59121.57	−.001	14899	3–	74021	4	1724.681	50	57981.74	−.001	14899	3–	72881	3
1692.584	25	59081.26	.003	22212	3–	81293	2	1724.855	250	57975.89	−.002	20432	4–	78408	4
											−.004	19851	3–	77827	4
1692.710	50.	59076.86	.003	16723	5–	75800	5								
1692.838	35	59072.40	.003	9904	0–	68976	1	1724.922	40	57973.64	−.004	23080	1–	81054	1
1694.022	30	59031.11	.003	27252	3–	86283	3	1725.933	100	57939.68	.000	2256	1–	60195	1
1694.371	100	59018.95	.001	27252	3–	86271	4	1726.897	100	57907.33	.000	36467	4–	94374	4
1694.497	40	59014.56	.001	23080	1–	82095	1	1728.009	150	57870.07	−.001	10968	1–	68838	0
1694.568	40	59012.09	.004	29800	2–	88813	3	1728.799	450	57843.62	−.005	15038	3–	72881	3
1694.635	15	59009.76	.003	36353	2–	95363	1	1729.329	350	57825.90	−.004	27252	3–	85078	4
1694.825	200	59003.14	.000	20696	2–	79699	3	1729.493	100	57820.41	−.002	29800	2–	87621	2
1695.286	15	58987.10	.002	56632	2–	115619	1	1730.374	5	57790.97	−.002	33972	2–	91763	3
1695.397	150	58983.24	.002	15038	3–	74021	4	1731.136	50	57765.54	−.003	29855	1–	87621	2
								1733.229	300	57695.78	−.004	23317	5–	81013	6
1695.666	150	58973.88	.003	16723	5–	75697	6								
1695.783	5	58969.81	.002	43378	4–	102348	3	1733.907	150	57673.22	−.001	31211	5–	88884	6
1696.217	50	58954.72	.000	36467	4–	95422	3	1735.611	40	57616.60	−.004	27252	3–	84869	4
1696.422	15	58947.60	.002	51002	3–	109950	3	1735.999	150	57603.72	−.004	22955	2–	80558	3
1696.797	20	58934.57	.001	19851	3–	78786	2	1736.499	50	57587.13	−.004	42615	3–	100202	4
1697.994	250	58893.02	.000	23317	5–	82210	5	1737.964	35	57538.59	−.003	26588	4–	84127	3
1698.099	350	58889.38	−.001	13992	2–	72881	3	1738.205	50	57530.61	−.003	33631	4–	91161	5
1698.239	50	58884.53	.002	28977	4–	87862	5	1740.026	100	57470.41	−.004	36904	5–	94374	4
1698.282	50	58883.04	.000	24097	1–	82980	2	1740.501	50	57454.72	−.003	9904	0–	67358	1
1698.935	50	58860.40	−.001	37769	4–	96630	3	1741.572	200	57419.39	−.001	7686	4–	65106	4
											−.003	18380	6–	75800	5
1699.431	15	58843.22	−.001	39344	2–	98188	1								
1699.571	5	58838.38	.001	51002	3–	109841	2	1742.157	40	57400.11	−.004	36467	4–	93867	4
1699.866	150	58828.17	.002	6277	3–	65106	4	1742.588	200	57385.91	−.008	22212	3–	79597	4
1700.623	5	58801.98	.003	28062	2–	86865	1	1743.529	100	57354.94	−.006	30507	4–	87862	5
1701.368	100	58776.23	.002	19632	5–	78408	4	1744.923	40	57309.12	−.008	39321	3–	96630	3
1701.630	40	58767.18	.004	42615	3–	101383	3	1745.252	150	57298.32	−.006	16723	5–	74021	4
1702.009	50	58754.10	.003	17380	4–	76134	3	1748.282	40	57199.01	−.008	42615	3–	99814	2
1702.065	20	58752.16	.000	37808	1–	96561	2	1748.366	2	57196.26	.001	24097	1–	81293	2
1702425	10	58739.74	.002	39321	3–	98061	2	1748.982	20	57176.12	−.001	23317	5–	80493	5
1703.368	40	58707.22	.002	20432	4–	79139	3	1750.211	5	57135.97	−.001	28391	3–	85527	2
								1750.354	1	57131.30	.009	36226	3–	93358	3
1703.713	100	58695.33	.000	18376	4–	77071	3								
1704.454	40	58669.81	.001	36467	4–	95137	5	1752.330	20	57066.88	−.007	29800	2–	86867	2
1705.483	100	58634.42	.000	26588	4–	85222	5	1752.406	15	57064.40	−.007	29800	2–	86865	1
1705.639	50	58629.05	.001	29800	2–	88429	3	1753.208	20	57038.30	.005	29245	3–	86283	3
1706.999	30	58582.34	.002	27252	3–	85834	3	1753.496	3	57028.93	.000	31838	1–	88867	0
1707.752	300	58556.51	−.001	19851	3–	78408	4	1753.973	1	57013.42	−.004	46259	2–	103272	1
1708.829	45	58519.61	.000	20696	2–	79216	1	1754.018	5	57011.96	−.006	29855	1–	86867	2
1708.950	50	58515.46	.000	28062	2–	86578	3	1754.087	2	57009.72	.005	47181	4–	104191	5
1709.703	50	58489.69	.001	26588	4–	85078	4	1754.233	50	57004.97	−.006	36353	2–	93358	3
			−.002	24490	2–	82980	2	1754.390	50	56999.87	−.009	36226	3–	93226	2
								1754.974	60b	56980.90	−.007	13700	4–	70681	3
1709.798	20	58486.44	.001	33631	4–	92117	4								
1710.092	20	58476.39	.001	28391	3–	86867	2	1755.440	80	56965.77	−.001	36904	5–	93870	6
1711.066	15	58443.10	.003	20696	2–	79139	3	1755.527	10	56962.95	−.002	36904	5–	93867	4
1711.126	100	58441.05	.002	35429	6–	93870	6	1756.612	80	56927.77	−.001	22212	3–	79139	3
1711.353	40	58433.30	.002	13992	2–	72425	1	1757.757	15	56890.69	.003	36467	4–	93358	3
1711.621	25	58424.15	.000	28062	2–	86487	2	1758.264	80	56874,28	,007	27252	3–	84127	3
1711.747	400	58419.85	−.001	17380	4–	75800	5				.001	36904	5–	93778	5
1712.010	10	58410.87	.005	57384	3–	115795	3	1758.427	3	56869.01	−.006	46259	2–	103127	3
			−.005	41403	2–	99814	2	1758.786	80	56857.40	.003	28977	4–	85834	3
1713.485	40	58360.59	.002	4461	2–	62821	2	1759.388	20	56837.95	.004	51002	3–	107840	4
								1759.801	50	56824.61	.003	43378	4–	100202	4
1760.310	3	56808.18	.004	42615	3–	99424	3	1791.422	100	55821.58	.001	41322	5–	97144	4
1760.479	1	56802.72	.004	24490	2–	81293	2	1791.688	2	55813.29	.002	39321	3–	95134	2
1761.055	30	56784.14	.001	41403	2–	98188	1	1792.147	10	55798.99	−.001	46259	2–	102058	1
1761.263	5	56777.44	.003	29800	2–	86578	3				−.003	29245	3–	85044	2
1761.496	60	56769.93	.006	13992	2–	70762	2	1792.448	2	55789.62	.001	39344	2–	95134	2
1762.282	50	56744.61	.004	22955	2–	79699	3	1792.702	1	55781.72	.004	14899	3–	70681	3
1762.471	10	56738.52	.005	37769	4–	94508	3	1792.872	2	55776.43	.002	30507	4–	86283	3
1762.754	1	56729.41	.005	50539	2–	107269	3	1793.115	2	55768.87	.002	31821	3–	87590	4
1763.996	30b	56689.47	.001	13992	2–	70681	3	1793.266	30	55764.18	.002	30507	4–	86271	4
1764.067	60	56687.19	.000	28391	3–	85078	4	1794.177	50	55735.86	.003	28391	3–	84127	3
1764.096	60	56686.26	−.001	29800	2–	86487	2	1794.214	30	55734.71	−.001	4461	2–	60195	1
1764.822	30	56662.94	.003	27726	0–	84389	1	1794.445	1	55727.54	.004	27252	3–	82980	2
1765.133	10	56652.95	.004	28391	3–	85044	2	1794.497	10	55725.92	.007	29800	2–	85527	2
1765.222	50	56650.10	.005	31211	5–	87862	5	1794.553	80	55724.18	.001	15038	3–	70762	2
1765.505	100	56641.02	−.002	17380	4–	74021	4	1794.783	10	55717.04	.001	41322	5–	97039	6
1765.810	40	56631.23	.004	29855	1–	86487	2	1795.149	300	55705.68	−.001	23080	1–	78786	2
1766.514	10	56608.67	.003	51002	3–	107611	2	1795.268	50	55701.99	.003	20432	4–	76134	3
1766.639	60	56604.66	.006	37769	4–	94374	4	1795.383	30	55698.42	.003	33631	4–	89329	3
1767.840	30	56566.21	.007	16723	5–	73289	5	1795.931	10	55681.43	.001	20696	2–	76378	1
1767.917	1	56563.74	.000	24490	2–	81054	1				−.003	52568	3–	108249	3
1768.358	50	56549.64	.002	12427	2–	68976	1	1796.262	3	55671.17	.003	29855	1–	85527	2
1768.534	80	56544.01	.001	6277	3–	62821	2	1797.112	200	55644.83	.004	18376	4–	74021	4
1769.278	20	56520.23	.005	13700	4–	70221	4				.001	47181	4–	102826	4
			−.008	33972	2–	90492	1	1797.147	10b	55643.75	−.005	15038	3–	70681	3
1770.192	1	56491.05	.000	41570	1–	98061	2	1797.796	50	55623.66	.002	29245	3–	84869	4
1770.452	20	56482.75	.004	29800	2–	86283	3	1797.843	20	55622.21	.003	26588	4–	82210	5
1770.607	80	56477.81	.004	28391	3–	84869	4	1797.906	50	55620.26	.001	36353	2–	91973	2
1773.357	80	56390.22	.005	10968	1–	67358	1	1798.061	60	55615.47	.003	22212	3–	77827	4
1773.584	50	56383.01	.002	10968	1–	67351	0	1798.941	60	55588.26	.001	37769	4–	93358	3
1773.713	80	56378.91	.001	31211	5–	87590	4	1800.192	20	55549.63	.004	36226	3–	91776	4
1773.833	10	56375.09	.001	20696	2–	77071	3	1800.220	30	55548.77	−.001	28062	2–	83611	3
1774.338	30	56359.05	.004	27252	3–	83611	3	1800.351	3	55544.72	.006	22212	3–	77757	2
1775.428	15	56324.45	.001	57384	3–	113709	3	1800.534	1	55539.08	−.002	28062	2–	83601	1
1775.615	300	56318.51	.000	27252	3–	83571	4	1803.588	40	55445.04	.003	42615	3–	98061	2
1775.658	200b	56317.15	−.004	46259	2–	102576	2	1803.819	200	55437.93	.001	20696	2–	76134	3
			−.006	17380	4–	73697	3	1804.185	8	55426.69	.006	23080	1–	78507	0
1776.776	1	56281.71	.000	29245	3–	85527	2	1804.734	10	55409.83	.004	36353	2–	91763	3
1776.836	50	56279.81	.004	23317	5–	79597	4	1805.631	50	55382.30	.003	41322	5–	96704	4
1777.426	30	56261.13	.002	22955	2–	79216	1	1806.112	50	55367.55	.004	20432	4–	75800	5
1777.450	20	56260.37	.001	16621	2–	72881	3	1806.439	50	55357.53	.003	33972	2–	89329	3
1777.920	60	56245.50	.001	28977	4–	85222	5	1806.673	10	55350.36	.005	16723	5–	72073	4
1779.006	1	56211.17	.003	56632	2–	112844	2	1807.413	1	55327.70	−.001	30507	4–	85834	3
1779.481	1	56196.16	.003	22212	3–	78408	4	1807.620	20	55321.36	.006	14899	3–	70221	4
1780.368	300	56168.16	.000	19632	5–	75800	5	1807.959	30	55310.99	.003	33972	2–	89283	1
1781.177	200	56142.65	.000	10968	1–	67111	2	1808.027	40	55308.91	.001	36467	4–	91776	4
1781.428	10	56134.74	.001	20696	2–	76831	2	1808.454	100	55295.85	.004	36467	4–	91763	3
1781.509	5	56132.19	.002	36226	3–	92358	2	1809.214	10	55272.62	.004	52568	3–	107840	4
1781.742	1	56124.85	.000	33972	2–	90097	3	1809.365	8	55268.01	.001	33972	2–	89240	2
1782.161	5	56111.65	.001	56732	1–	112844	2	1809.974	1	55249.41	.005	27252	3–	82502	4
1782.507	20	56100.76	.002 −.001	28977 39321	4-3–	85078 95422	4 3	1810.739	1	55226.07	.003	41403	2–	96630	3
								1811.253	40	55210.40	.005	6277	3–	61488	3
1782.613	20	56097.43	.001	37769	4–	93867	4	1811.295	20	55209.12	.002	24490	2–	79699	3
1782.666	20	56095.76	.000	12881	1–	68976	1	1811.978	30	55188.31	.004	29855	1–	85044	2
1783.451	10	56071.07	.002	30507	4–	86578	3	1812.009	1b	55187.36	−.006	39321	3–	94508	3
1783.522	15 '	56068.83	.005	33631	4–	89700	4	1812.144	500	55183.25	.002	15038	3–	70221	4
1783.551	10b	56067.92	.004	24490	2–	80558	3	1812.245	5	55180.18	−.007	28391	3–	83571	4
1783.636	1000	56065.25	−.003	19632	5–	75697	6	1812.795	1	55163.44	.003	39344	2–	94508	3
1784.252	5b	56045.89	.003	43378	4–	99424	3	1813.004	10	55157.08	.001	41403	2–	96561	2
1784.631	30	56033.99	.003	29800	2–	85834	3	1813.254	40	55149.47	.004	28977	4–	84127	3
1785.729	100	55999.54	.001	33631	4–	89630	5	1813.707	30	55135.70	.006	36226	3–	91362	2
1787.072	10	55957.45	.003	12881	1–	68838	0								
								1813.877	5	55130.53	−.003	50539	2–	105670	2
1787.429	5	55946.28	.002	47181	4–	103127	3	1814.001	2	55126.76	.001	41403	2–	96530	1
1787.929	1	55930.63	.001	33972	2–	89903	1	1814.095	1	55123.90	.006	46259	2–	101383	3
1788.292	10	55919.28	.001	45388	2–	101307	1	1814.257	5	55118.98	.004	24097	1–	79216	1
1788.480	100	55913.40	.004	26588	4–	82502	4	1815.199	10	55090.38	.006	23317	5–	78408	4
1788.610	500	55909.34	−.002	17380	4–	73289	5	1815.469	800	55082.19	.003	35429	6–	90511	7
1788.746	1	55905.09	−.001	41322	5–	97227	5	1816.213	20	55059.62	.005	31211	5–	86271	4
1789.181	80	55891.49	.002	28977	4–	84869	4	1816.426	10	55053.16	.006	39321	3–	94374	4
			−.002	36226	3–	92117	4	1816.545	10	55049.56	.004	25963	6–	81013	6
1789.688	1	55875.66	.003	27726	0–	83601	1	1816.676	50	55045.59	.005	31821	3–	86867	2
1791.105	2	55831.46	−.001	22955	2–	78786	2								
1816.792	1	55042.07	.005	51002	3–	106044	3	1851.392	1	54013.41	−.008	31821	3–	85834	3
1817.232	1	55028.75	.006	31838	1–	86867	2	1851.468	20	54011.20	.000	31211	5–	85222	5
1818.479	2	54991.01	.002	41570	1–	96561	2	1851.632	25	54006.41	.001	37769	4–	91776	4
1818.696	5	54984.45	.007	13992	2–	68976	1	1852.882	40b	53969.98	.004	26588	4–	80558	3
1818.859	300	54979.52	.002	28977	4–	83957	5	1853.254	50	53959.14	.004	33631	4–	87590	4
1819.020	10	54974.66	.006	2256	1–	57231	2				.002	41403	2–	95363	1
1820.170	10	54939.92	.005	19851	3–	74791	2	1853.735	5	53945.14	.000	42615	3–	96561	2
1820.442	200	54931.71	.004	12427	2–	67358	1	1854.516	30	53922.43	.003	22212	3–	76134	3
1820.928	5	54917.05	.006	28062	2–	82980	2	1855.103	80	53905.36	.000	26588	4–	80493	5
1821.049	500	54913.40	−.005	18376	4–	73289	5	1856.100	50	53876.41	−.002	22955	2–	76831	2
1822.104	10	54881.61	.005	29245	3–	84127	3	1856.294	1	53870.78	−.001	36226	3–	90097	3
1822.254	30	54877.09	.005	29512	0–	84389	1	1856.438	1	53866.60	−.003	31211	5–	85078	4
1822.840	30	54859.45	.007	22212	3–	77071	3	1857.034	50	53849.31	.002	43378	4–	97227	5
1823.466	2	54840.62	.005	33972	2–	88813	3	1857.166	30	53845.48	.000	19851	3–	73697	3
1824.757	60	54801.82	.005	22955	2–	77757	2	1857.771	30	53827.95	−.008	22212	3–	76039	2
1826.265	40	54756.57	.003	31821	3–	86578	3	1858.350	20	53811.18	.002	57384	3–	111196	4
1826.357	300	54753.81	.001	31211	5–	85965	6	1858.549	2	53805.41	.001	47577	2–	101383	3
1826.858	20	54738.79	.003	28062	2–	82801	2	1858.679	5	53801.65	.001	7686	4–	61488	3
1827.297	30	54725.64	.001	24490	2–	79216	1	1858.972	40	53793.17	−.001	41570	1–	95363	1
1827.633	80	54715.58	.004	30507	4–	85222	5	1859.913	50	53765.96	−.002	43378	4–	97144	4
1828.121	1	54700.97	.004	52568	3–	107269	3	1860.148	5	53759.16	−.004	51002	3–	104761	2
1828.381	60	54693.20	.005	17380	4–	72073	4	1860.443	5	53750.64	−.002	23080	1–	76831	2
1828.517	20	54689.13	.007	24097	1–	78786	2	1860.524	20	53748.30	−.001	50539	2–	104287	2
1828.954	60	54676.06	.004	23080	1–	77757	2	1860.675	2	53743.94	−.002	36353	2–	90097	3
1829.316	30	54665.24	.003	31821	3–	86487	2	1861.137	10b	53730.60	.002	41403	2–	95134	2
1829.856	20	54649.11	.004	24490	2–	79139	3	1861.150	10b	53730.22	−.006	47577	2–	101307	1
1830.359	80	54634.09	.005	28977	4–	83611	3	1862.598	60	53688.45	−.001	31838	1–	85527	2
1830.860	10	54619.14	.006	22212	3–	76831	2	1863.591	30	53659.84	.001	24097	1–	77757	2
1831.722	60	54593.44	.005	28977	4–	83571	4	1863.679	50	53657.31	−.002	31211	5–	84869	4
1831.873	60	54588.94	.005	28391	3–	82980	2	1864.631	80	53629.91	.000	36467	4–	90097	3
1832.154	1	54580.56	.006	47995	1–	102576	2	1864.981	80	53619.85	−.003	30507	4–	84127	3
1832.479	20	54570.88	.004	30507	4–	85078	4	1866.062	60	53588.79	.001	20432	4–	74021	4
1833.323	10	54545.76	.007	39321	3–	93867	4	1866.905	1	53564.59	.000	41570	1–	95134	2
1833.742	100	54533.30	.007	29855	1–	84389	1	1867.295	30	53553.40	.000	37808	1–	91362	2
1833.853	5	54530.00	.003	25963	6–	80493	5	1868.299	50	53524.62	.001	28977	4–	82502	4
1833.918	10	54528.07	.005	42615	3–	97144	4	1869.226	40	53498.08	.001	16723	5–	70221	4
1834.536	200	54509.70	.005	23317	5–	77827	4	1869.643	20	53486.15	.010	13700	4–	67187	3
1834.695	1	54504.97	.004	18376	4–	72881	3	1869.967	5	53476.88	.001	52568	3–	106044	3
1835.612	100	54477.74	.005	12881	1–	67358	1	1870.068	80	53473.99	−.001	36226	3–	89700	4
1836.147	60	54461.87	.004	31821	3–	86283	3	1870.700	150	53455.93	.001	35429	6–	88884	6
1836.295	50	54457.48	.004	33972	2–	88429	3	1870.916	100	53449.75	.000	30507	4–	83957	5
1836.559	40	54449.65	.004	31821	3–	86271	4	1872.930	20	53392.28	−.004	37769	4–	91161	5
1837.096	10	54433.74	.000	39344	2–	93778	1	1873.823	20	53366.83	−.002	13992	2–	67358	1
1837.361	20	54425.89	.009	45388	2–	99814	2	1875.182	20	53328.16	−.002	27726	0–	81054	1
1837.876	50	54410.63	.003	28391	3–	82801	2	1875.231	40	53326.76	−.003	43378	4–	96704	4
			−.003	24097	1–	78507	0	1875.550	1	53317.69	−.004	56632	2–	109950	3
1839.279	2	54369.13	.008	47577	2–	101947	2	1875.957	60	53306.13	−.001	27252	3–	80558	3
			.003	27726	0–	82095	1	1876.131	80	53301.18	.002	17380	4–	70681	3
1839.990	1	54348.12	.000	37769	4–	92117	4	1876.263	50	53297.43	−.006	23080	1–	76378	1
1840.746	100	54325.80	−.001	29245	3–	83571	4	1876.697	10b	53285.11	.001	51002	3–	104287	2
1841.764	1	54295.77	.004	24490	2–	78786	2	1876.736	60	53284.00	−.001	19851	3–	73135	2
1842.277	40	54280.65	.003	37808	1–	92089	0	1877.351	2	53266.54	−.004	24490	2–	77757	2
1843.065	80	54257.45	.005	36904	5–	91161	5	1877.409	1	53264.90	−.003	20432	4–	73697	3
1843.192	10	54253.71	.002	55564	4–	109818	4	1877.697	10	53256.73	.003	29245	3–	82502	4
1843.995	100	54230.08	.003	12881	1–	67111	2				−.007	31821	3–	85078	4
1844.953	1	54201.92	.002	35429	6–	89630	5	1878.521	40	53233.37	.001	28977	4–	82210	5
1846.062	200	54169.36	.004	19851	3–	74021	4				−.008	36467	4–	89700	4
1846.225	1	54164.58	.003	37808	1–	91973	2	1878.620	5	53230.56	−.003	28062	2–	81293	2
1846.627	5	54152.79	.002	43378	4–	97531	3	1879.502	40	53205.58	.001	31838	1–	85044	2
1847.030	30	54140.97	.007	16621	2–	70762	2	1879.866	30	53195.28	−.002	13992	2–	67187	3
1847.860	30	54116.65	.002	22955	2–	77071	3	1880.422	60	53179.55	.001	22955	2–	76134	3
1848.053	200	54111.00	.000	28391	3–	82502	4				−.008	29800	2–	82980	2
1848.592	80	54095.22	.004	20696	2–	74791	2	1880.981	40	53163.75	−.002	36467	4–	89630	5
1848.773	10	54089.93	.001	29512	0–	83601	1	1882.372	25	53124.46	−.001	29855	1–	82980	2
1848.806	50	54088.96	.001	42615	3–	96704	4	1882.561	2	53119.13	−.002	13992	2–	67111	2
1849.783	60	54060.40	.005	16621	2–	70681	3	1882.840	2	53111.26	.002	56632	2–	109744	1
1850.450	60	54040.91	.000	27252	3–	81293	2				−.002	26588	4–	79699	3
1850.595	20	54036.67	.004	39321	3–	93358	3	1883.081	20	53104.46	.001	41403	2–	94508	3
1850.791	100	54030.95	.001	13700	4–	67731	5				−.001	30507	4–	83611	3
1851.235	20	54017.99	.001	41403	2–	95422	3	1883.111	10	53103.61	−.004	36226	3–	89329	3
1884.636	1	53060.64	.000	52568	3–	105628	4	1927.253	300	51887.32	.007	24490	2–	76378	1
1885.109	20	53047.33	−.003	31821	3–	84869	4				−.002	27252	3–	79139	3
1885.456	1	53037.57	−.005	39321	3–	92358	2	1928.175	50	51862.51	.003	33972	2–	85834	3
1885.737	20	53029.66	−.001	19851	3–	72881	3	1928.223	80	51861.22	.000	37769	4–	89630	5
1886.372	10	53011.81	−.003	56732	1–	109744	1	1928.830	30	51844.90	.002	18376	4–	70221	4
1886.460	80	53009.34	−.005	26588	4–	79597	4	1929.129	3	51836.87	.002	22955	2–	74791	2
1886.761	2	53000.88	.003	29800	2–	82801	2	1929.766	80	51819.75	.002	26588	4–	78408	4
			−.005	20696	2–	73697	3	1930.157	3	51809.26	.000	22212	3–	74021	4
1889.279	2	52930.24	−.005	36353	2–	89283	1	1932.040	50	51758.76	.004	43378	4–	95137	5
1890.619	20	52892.73	−.001	33972	2–	86865	1				−.003	42615	3–	94374	4
1890.821	20	52887.08	.000	36353	2–	89240	2	1932.394	20	51749.28	.000	31821	3–	83571	4
1891.692	20	52862.73	−.002	36467	4–	89329	3	1933.819	1	51711.15	−.001	23080	1–	74791	2
1892.472	40	52840.94	.000	17380	4–	70221	4	1934.102	200b	51703.58	−.001	30507	4–	82210	5
1893.721	50	52806.09	−.001	42615	3–	95422	3	1935.886	15	51655.93	.003	41570	1–	93226	2
1894.064	30	52796.53	.003	39321	3–	92117	4	1936334	10	51643.98	.001	24490	2–	76134	3
1895.902	200	52745.34	−.003	31211	5–	83957	5	1936.604	2	51636.78	.001	28062	2–	79699	3
1896.573	60	52726.68	−.002	36904	5–	89630	5	1937.361	60	51616.61	.000	56632	2–	108249	3
1898.078	10	52684.87	−.002	55564	4–	108249	3	1938.303	2	51591.52	.001	33631	4–	85222	5
1898.515	20	52672.75	−.008	45388	2–	98061	2	1938.694	20	51581.12	.002	28977	4–	80558	3
1899.250	40	52652.36	−.001	39321	3–	91973	2	1939.483	25	51560.13	.001	37769	4–	89329	3
			−.002	33631	4–	86283	3								
								1939.690	2	51554.63	.002	33972	2–	85527	2
1899.686	60	52640.28	−.007	33631	4–	86271	4	1939.898	80	51549.10	.004	24490	2–	76039	2
1900.102	5	52628.75	−.003	39344	2–	91973	2	1940.154	1	51542.30	.001	29512	0–	81054	1
1900.922	10	52606.05	.001	33972	2–	86578	3	1940.471	50	51533.88	.002	27252	3–	78786	2
1901.736	1	52583.53	−.004	29512	0–	82095	1	1941.131	60	51516.36	.003	28977	4–	80493	5
1901.822	50	52581.16	.000	24490	2–	77071	3	1942.032	80	51492.46	.004	29800	2–	81293	2
1901.863	10b	52580.02	−.006	22212	3–	74791	2	1942.307	30	51485.17	.004	22212	3–	73697	3
1902.923	20	52550.73	−.002	31838	1–	84389	1	1942.712	30	51474.43	.002	37808	1–	89283	1
1903.034	200	52547.67	−.003	41322	5–	93870	6	1943.756	40	51446.79	.002	33631	4–	85078	4
1903.140	20	52544.74	−.001	41322	5–	93867	4	1945.336	50	51405.00	.004	13700	4–	65106	4
1904.225	1	52514.80	−.002	33972	2–	86487	2								
								1945.728	80	51394.65	.004	36226	3–	87621	2
1904.907	1	52496.00	−.001	52568	3–	105064	4				.003	36467	4–	87862	5
1905.400	80	52482.42	−.001	23317	5–	75800	5	1946.882	60	51364.18	.002	36226	3–	87590	4
1906.223	20	52459.76	.001	36353	2–	88813	3	1948.990	10	51308.63	.001	28391	3–	79699	3
1906.395	100b	52455.03	−.003	39321	3–	91776	4	1949.685	1	51290.34	−.001	31211	5–	82502	4
1906.680	80	52447.19	.001	27252	3–	79699	3	1951.171	60	51251.27	.001	42615	3–	93867	4
1906.880	300	52441.68	.001	19632	5–	72073	4	1951.541	40	51241.56	.001	45388	2–	96630	3
1907.194	60	52433.05	−.001	35429	6–	87862	5	1951.693	60	51237.57	.001	33631	4–	84869	4
1907.730	15	52418.32	.000	39344	2–	91763	3	1952.872	50	51206.63	.000	28391	3–	79597	4
1909.146	80	52379.44	−.001	23317	5–	75697	6	1953.181	30	51198.53	.002	29855	1–	81054	1
1909.878	30	52359.37	−.003	31211	5–	83571	4								
								1954.712	20	51158.43	.000	31821	3–	82980	2
1910.011	1	52355.72	.000	16621	2–	68976	1	1954.812	5	51155.81	.000	27252	3–	78408	4
1910.393	50	52345.25	−.002	27252	3–	79597	4	1954.905	40	51153.38	−.003	28062	2–	79216	1
1910.554	1	52340.84	−.001	24490	2–	76831	2	1955.134	40	51147.39	.000	39344	2–	90492	1
1911.043	40	52327.45	−.001	37769	4–	90097	3	1955.326	1	51142.37	−.006	45388	2–	96530	1
1911.629	1	52311.41	.000	33972	2–	86283	3	1957.832	2	51076.91	−.002	28062	2–	79139	3
1911.854	100	52305.25	.001	31821	3–	84127	3	1958.029	60	51071.77	.003	33972	2–	85044	2
			−.001	18376	4–	70681	3	1959.036	1	51045.51	.003	24097	1–	75142	0
1912.246	80	52294.53	.002	29800	2–	82095	1	1960.451	80	51008.67	.001	16723	5–	67731	5
1912.502	80	52287.53	.002	14899	3–	67187	3	1960.823	5	50998.99	.003	31211	5–	82210	5
1914.253	5	52239.70	−.001	29855	1–	82095	1								
								1961.552	1	50980.04	.001	31821	3–	82801	2
1914.906	1	52221.88	.000	19851	3–	72073	4	1962.416	80	50957.60	.002	36904	5–	87862	5
1915.291	50	52211.39	.000	14899	3–	67111	2	1962.528	1	50954.69	.002	41403	2–	92358	2
1915.389	5	52208.72	−.002	41570	1–	93778	1	1962.587	2	50953.16	.003	6277	3–	57231	2
1915.579	10	52203.54	−.001	33631	4–	85834	3	1963.722	10	50923.71	.002	22212	3–	73135	2
			−.002	36226	3–	88429	3	1964.237	1	50910.35	.001	19851	3–	70762	2
1916.899	1	52167.59	−.002	28391	3–	80558	3	1966.010	1	50864.44	.002	57384	3–	108249	3
1917.572	2	52149.28	.002	15038	3–	67187	3	1967.356	20	50829.64	.005	19851	3–	70681	3
1917.818	30	52142.59	−.002	45388	2–	97531	3	1969.439	10	50775.88	.002	39321	3–	90097	3
1918.457	2	52125.22	.001	51002	3–	103127	3	1970.145	20	50757.69	.003	29800	2–	80558	3
1920.378	100	52073.08	.003	15038	3–	67111	2								
								1970.494	50	50748.70	.001	28391	3–	79139	3
1920.791	60	52061.88	.001	23080	1–	75142	0	1970.743	100	50742.28	.001	22955	2–	73697	3
1921.297	5	52048.17	−.001	29245	3–	81293	2				−.006	42615	3–	93358	3
1921.468	20	52043.54	.008	43378	4–	95422	3	1970.919	40	50737.75	.003	16621	2–	67358	1
1921.725	1	52036.58	.001	50539	2–	102576	2	1971.464	200	50723.73	.002	23080	1–	73804	1
1922.433	1	52017.42	.000	39344	2–	91362	2				−.008	28062	2–	78786	2
1923.269	80	51994.81	.000	30507	4–	82502	4	1972.252	5	50703.46	.004	23317	5–	74021	4
1923.794	500	51980.62	−.001	36904	5–	88884	6	1972.925	80	50686.16	.006	36904	5–	87590	4
1924.781	40	51953.96	.005	41403	2–	93358	3	1973.153	1	50680.31	.001	31821	3–	82502	4
1925.201	1	51942.63	.001	24097	1–	76039	2	1973.580	15	50669.34	.003	22212	3–	72881	3
1925.653	5	51930.44	.008	37769	4–	89700	4								
1974.684	5	50641.01	.001	36226	3–	86867	2	2020.753	100	49470.55	−.003	22955	2–	72425	1
1975.493	80	50620.28	.001	28977	4–	79597	4	2020.850	60	49468.17	−.001	39344	2–	88813	3
1975.881	1	50610.34	−.001	47577	2–	98188	1	2021.395	2h	49454.84	.000	31838	1–	81293	2
1976.701	800	50589.34	−.002	19632	5–	70221	4	2022.144	100	49436.52	.001	28391	3–	77827	4
1977.606	50	50566.19	.004	16621	2–	67187	3	2022.370	40	49431.00	−.004	28977	4–	78408	4
1977.928	60	50557.96	.004	39344	2–	89903	1	2025.037	30h	49365.91	.000	28391	3–	77757	2
1978.765	80	50536.57	.001	35429	6–	85965	6	2025.468	5h	49355.40	.006	18376	4–	67731	5
1979.425	2	50519.72	−.002	41570	1–	92089	0	2025.652	40	49350.92	.005	18380	6–	67731	5
1979.741	1	50511.66	.003	36353	2–	86865	1	2025.908	80	49344.69	.000	23080	1–	72425	1
1980.022	60	50504.49	−.001	27252	3–	77757	2	2026.142	5H	49338.99	−.002	29800	2–	79139	3
1980.590	100	50490.01	.004	16621	2–	67111	2	2027.169	20	49314.00	.001	24490	2–	73804	1
1980.628	20b	50489.04	−.001	43378	4–	93867	4	2027.724	1h	49300.50	.002	36226	3–	85527	2
1980.859	20	50483.15	.004	47577	2–	98061	2	2028.482	100	49282.08	.002	31211	5–	80493	5
			.002	26588	4–	77071	3	2030.744	100	49227.19	.005	10968	1–	60195	1
1981.985	2	50454.47	.000	29245	3–	79699	3	2031.220	80	49215.66	.004	31838	1–	81054	1
1983.463	3	50416.87	.002	33972	2–	84389	1	2031.389	2h	49211.57	.004	26588	4–	75800	5
1984.331	80	50394.82	−.002	12427	2–	62821	2	2031.588	40	49206.75	.001	24490	2–	73697	3
1984.899	5	50380.40	.005	51002	3–	101383	3	2031.858	1	49200.21	.001	51002	3–	100202	4
1984.951	100	50379.08	.003	39321	3–	89700	4	2032.170	5h	49192.66	−.007	30507	4–	79699	3
1985.330	1	50369.46	.001	19851	3–	70221	4	2035.648	10	49108.62	.002	39321	3–	88429	3
1985.732	60	50359.26	.001	41403	2–	91763	3	2036.403	20	49090.41	.002	30507	4–	79597	4
1986.033	1000	50351.63	−.004	17380	4–	67731	5	2036.627	40	49085.02	−.001	39344	2–	88429	3
1988.015	5	50301.43	−.002	24490	2–	74791	2	2037.614	900	49061.24	.001	12427	2–	61488	3
1988.407	60	50291.51	.002	9904	0–	60195	1				−.001	36904	5–	85965	6
1989.192	1	50271.67	−.003	46259	2–	96530	1	2037.731	80	49058.43	.006	37808	1–	86867	2
1989.778	20	50256.86	−.001	31838	1–	82095	1	2037.829	1h	49056.07	.002	37808	1–	86865	1
1991.035	50	50225.13	−.001	36353	2–	86578	3	2038.554	40	49038.62	.003	24097	1–	73135	2
1991.777	800	50206.42	−.007	14899	3–	65106	4	2039.799	100	49008.70	.003	28062	2–	77071	3
1992.321	10	50192.71	−.001	47995	1–	98188	1	2042.765	10	48937.55	.004	56732	1–	105670	2
1992.792	3	50180.85	−.002	22955	2–	73135	2	2045.085	40	48882.04	.001	27252	3–	76134	3
1993.525	2	50162.40	−.001	28977	4–	79139	3	2045.561	30	48870.67	−.001	33631	4–	82502	4
1993.828	1	50154.78	−.003	33972	2–	84127	3	2046.418	60	48850.20	.000	28977	4–	77827	4
1994.660	5	50133.86	−.003	36353	2–	86487	2	2047.280	200	48829.64	.003	13992	2–	62821	2
1996.316	30	50092.27	−.001	37769	4–	87862	5				−.007	33972	2–	82801	2
1997.286	800	50067.94	.003	15038	3–	65106	4	2047.782	40	48817.67	.002	36226	3–	85044	2
1997.376	20	50065.69	−.002	20696	2–	70762	2	2047.888	1h	48815.14	.002	52568	3–	101383	3
			−.003	47995	1–	98061	2	2048.067	500	48810.88	.004	18376	4–	67187	3
1997.801	20	50055.04	−.001	23080	1–	73135	2	2048.159	1b	48808.68	−.002	37769	4–	86578	3
1997.948	15	50051.35	.000	30507	4–	80558	3	2049.062	100	48787.18	.004	27252	3–	76039	2
1998.160	20	50046.04	−.002	47181	4–	97227	5	2049.849	40	48768.45	.000	28062	2–	76831	2
1998.659	8	50033.55	−.004	45388	2–	95422	3	2050.378	100	48755.87	.003	23317	5–	72073	4
1999.652	1	50008.70	−.001	39321	3–	89329	3				−.008	36467	4–	85222	5
1999.672	2	50008.20	−.003	52568	3–	102576	2	2050.782	1	48746.27	.006	42615	3–	91362	2
1999.887	80	50002.83	.000	30507	4–	80493	5	2051.064	60	48739.56	.003	43378	4–	92117	4
1999.950	40b	50001.25	−.002	39344	2–	89329	3	2051.178	1	48736.86	.000	31821	3–	80558	3
			−.002	20696	2–	70681	3	2053.545	200	48680.69	−.002	28391	3–	77071	3
2000.480	80	49971.81	.002	23317	5–	73289	5	2054.418	5h	48660.00	.002	57384	3–	106044	3
2000.850	30h	49962.57	−.002	47181	4–	97144	4	2054.764	30h	48651.81	.006	29855	1–	78507	0
2001.020	5h	49958.33	.002	41403	2–	91362	2				.002	27726	0–	76378	1
2001.216	1	49953.44	−.006	47577	2–	97531	3	2055.042	200	48645.23	.001	24490	2–	73135	2
2002.139	10	49930.41	.001	36353	2–	86283	3	2057.703	20	48582.33	.001	29245	3–	77827	4
2003.544	2	49895.40	.002	39344	2–	89240	2	2057.830	300	48579.33	.003	33631	4–	82210	5
2003.989	30	49884.32	−.003	57384	3–	107269	3	2058.413	2	48565.58	.003	47995	1–	96561	2
2005.799	300	49839.32	.004	41322	5–	91161	5	2059.059	2h	48550.34	−.007	22212	3–	70762	2
2006.539	40	49820.94	−.001	37769	4–	87590	4	2060.009	30h	48527.95	.003	35429	6–	83957	5
2006.606	2	49819.28	−.003	27252	3–	77071	3	2061.125	2	48501.68	.002	37769	4–	86271	4
2006.729	10	49816.22	.010	36467	4–	86283	3	2063.739	100b	48440.26	.002	28391	3–	76831	2
2007.104	800	49806.92	.002	17380	4–	67187	3	2064.534	1	48421.61	−.004	51002	3–	99424	3
2007.214	1h	49804.19	.002	36467	4–	86271	4	2065.386	1h	48401.63	.004	36467	4–	84869	4
2007.313	500	49801.73	−.001	31211	5–	81013	6	2065.839	1	48391.02	−.004	24490	2–	72881	3
2007.622	200	49794.07	−.004	35429	6–	85222	5	2066.054	10H	48385.99	−.001	31211	5–	79597	4
2007.836	20h	49788.76	.002	20432	4–	70221	4	2066.093	15H	48385.07	−.003	43378	4–	91763	3
2008.186	lh	49780.08	−.001	52568	3–	102348	3	2066.405	10	48377.77	.005	41322	5–	89700	4
2009.695	5	49742.71	.002	42615	3–	92358	2	2068.938	100	48318.55	.002	36904	5–	85222	5
2011.250	30	49704.26	−.001	29512	0–	79216	1	2069.372	50	48308.42	.001	41322	5–	89630	5
2011.659	60h	49694.15	−.004	28062	2–	77757	2	2070.371	30	48285.11	.000	57384	3–	105670	2
2016.340	100	49578.81	.002	27252	3–	76831	2	2070.583	5	48280.17	.001	20696	2–	68976	1
2017.674	200	49546.03	.001	26588	4–	76134	3	2070.753	2	48276.20	.002	39344	2–	87621	2
2018.605	1h	49523.18	.005	47181	4–	96704	4	2078.347	800	48099.83	.003	19632	5–	67731	5
2019.472	1	49501.92	.001	42615	3–	92117	4	2078.591	20	48094.18	.005	28977	4–	77071	3
2079.850	10	48065.08	.004	37769	4–	85834	3	2142.065	30	46669.22	−.005	31838	1–	78507	0
2083.680	20	47976.74	.005	28062	2–	76039	2	2142.186	1	46666.58	.004	36904	5–	83571	4
2084.209	50	47964.56	.003	36904	5–	84869	4	2142.383	60	46662.29	.000	20696	2–	67358	1
2085.897	3	47925.75	.010	41403	2–	89329	3	2143.423	10	46639.65	.000	56632	2–	103272	1
2086.062	50	47921.96	.004	14899	3–	62821	2	2144.011	5	46626.86	.001	36353	2–	82980	2
2086.971	20	47901.09	.007	29855	1–	77757	2	2144.516	300	46615.89	.000	31211	5–	77827	4
			−.001	30507	4–	78408	4	2145.386	100	46596.98	.003	47181	4–	93778	5
2089.456	10	47844.13	.002	47577	2–	95422	3	2145.775	800	46588.54	.001	14899	3–	61488	3
2089.793	20b	47836.42	.000	41403	2–	89240	2	2145.865	500	46586.58	.000	31821	3–	78408	4
2091.071	40	47807.18	.002	22955	2–	70762	2	2145.936	100	46585.04	.001	45388	2–	91973	2
2091.938	30	47787.37	.002	13700	4–	61488	3	2146.293	lOh	46577.30	−.003	29800	2–	76378	1
2092.096	900	47783.76	.002	15038	3–	62821	2	2146.887	100	46564.41	.002	30507	4–	77071	3
			−.003	43378	4–	91161	5	2148.012	10	46540.02	.002	56732	1–	103272	1
2092.441	30h	47775.89	.004	31821	3–	79597	4	2148.828	60	46522.35	−.001	29855	1–	76378	1
2092.545	40h	47773.51	.009	36353	2–	84127	3	2150.084	5	46495.18	.000	56632	2–	103127	3
2092.755	800	47768.72	.002	12427	2–	60195	1	2150.329	1	46489.88	.005	39344	2–	85834	3
2093.857	10	47743.58	−.003	28391	3–	76134	3	2152.161	800	46450.31	.000	15038	3–	61488	3
2094.648	800b	47725.55	.004	17380	4–	65106	4	2152.243	20b	46448.54	−.002	36353	2–	82801	2
2094.982	lh	47717.95	.005	37808	1–	85527	2	2152.417	100	46444.79	.001	27252	3–	73697	3
2095.180	20	47713.44	−.001	41570	1–	89283	1	2153.822	80	46414.50	.004	20696	2–	67111	2
2096.588	20	47681.40	.002	23080	1–	70762	2	2154.452	40	46400.92	−.003	28391	3–	74791	2
2097.076	40	47670.30	.003	41570	1–	89240	2	2156.477	1	46357.36	−.005	37769	4–	84127	3
2098.032	1	47648.58	.006	28391	3–	76039	2	2157.580	30	46333.66	.004	29800	2–	76134	3
			−.002	50539	2–	98188	1	2159.473	10	46293.05	−.004	26588	4–	72881	3
2101.837	10	47562.34	.003	41322	5–	88884	6	2160.290	2h	46275.54	.004	36226	3–	82502	4
2102.142	40	47555.44	.002	56732	1–	104287	2	2160.468	60	46271.73	−.002	24490	2–	70762	2
2102.551	40	47546.19	.002	39321	3–	86867	2	2160.635	2	46268.16	.000	41322	5–	87590	4
2102.852	200	47539.38	.002	27252	3–	74791	2	2160.897	500	46262.55	−.002	10968	1–	57231	2
2103.726	80	47519.63	−.005	46259	2–	93778	1	2161.352	30	46252.81	−.003	43378	4–	89630	5
2104.761	300b	47496.27	−.002	13992	2–	61488	3	2162.004	5h	46238.86	.004	29800	2–	76039	2
2106.673	200	47453.17	.001	37769	4–	85222	5	2163.646	60	46203.77	−.003	13992	2–	60195	1
2108.630	2h	47409.13	.000	41403	2–	88813	3	2163.788	800b	46200.74	.005	47577	2–	93778	1
2109.690	lh	47385.31	−.004	36226	3–	83611	3				.001	16621	2–	62821	2
2110.027	40	47377.75	−.004	31838	1–	79216	1	2163.959	10	46197.09	.003	42615	3–	88813	3
2110.474	30	47367.71	.000	47995	1–	95363	1	2164.242	60	46191.05	.000	24490	2–	70681	3
2111.502	20	47344.65	−.003	36226	3–	83571	4	2164.420	100	46187.25	.000	37769	4–	83957	5
2111.907	10	47335.58	−.003	19851	3–	67187	3	2164.567	1	46184.12	−.003	29855	1–	76039	2
2112.293	1	47326.93	−.004	47181	4–	94508	3	2164.927	10	46176.44	.000	47181	4–	93358	3
2112.582	30	47320.45	−.004	30507	4–	77827	4	2168.970	lh	46090.37	.008	50539	2–	96630	3
2112.688	80	47318.08	−.001	31821	3–	79139	3	2169.530	1	46078.48	−.005	27726	0–	73804	1
2112.830	100	47314.90	−.003	12881	1–	60195	1	2170.808	lh	46051.35	−.008	41570	1–	87621	2
2113.121	1	47308.39	.005	37769	4–	85078	4	2171.588	30	46034.81	−.001	36467	4–	82502	4
2113.518	40	47299.50	−.004	20432	4–	67731	5	2172.202	80	46021.80	−.002	22955	2–	68976	1
2114.797	10	47270.90	−.001	29800	2–	77071	3	2174.469	40	45973.83	−.001	45388	2–	91362	2
2115.407	5h	47257.27	−.006	39321	3–	86578	3	2174.827	100	45966.26	.002	33631	4–	79597	4
2116.470	5h	47233.54	−.003	39344	2–	86578	3	2175.906	30	45943.47	−.003	56632	2–	102576	2
2118.121	40	47196.72	−.006	31211	5–	78408	4	2177.091	60	45918.47	−.002	31838	1–	77757	2
2118.285	5	47193.07	−.003	47181	4–	94374	4	2178.157	lh	45896.00	−.001	23080	1–	68976	1
2119.507	30	47165.86	−.002	39321	3–	86487	2	2182.052	20b	45814.08	−.004	42615	3–	88429	3
2119.898	80	47157.17	.000	28977	4–	76134	3	2182.657	1	45801.38	−.006	37769	4–	83571	4
2120.570	50	47142.22	−.003	39344	2–	86487	2	2183.055	1	45793.03	−.001	37808	1–	83601	1
2122.521	1	47098.90	.000	46259	2–	93358	3	2183.516	15	45783.37	−.006	47995	1–	93778	1
2123.168	1	47084.54	−.003	42615	3–	89700	4	2183.658	1	45780.39	−.007	47577	2–	93358	3
2123.279	20	47082.08	−.004	33972	2–	81054	1	2185.424	500	45743.40	.007	36467	4–	82210	5
2124.606	80	47052.68	−.001	36904	5–	83957	5	2185.498	50b	45741.85	−.010	28062	2–	73804	1
2125.605	5h	47030.57	−.002	29800	2–	76831	2	2186.400	1	45722.98	−.003	39321	3–	85044	2
2125.812	2h	47025.99	−.001	41403	2–	88429	3	2187.390	5	45702.29	−.004	51002	3–	96704	4
2127.374	30	46991.47	.002	50539	2–	97531	3	2187.532	20	45699.32	−.004	39344	2–	85044	2
2128.087	2H	46975.72	−.004	29855	1–	76831	2	2190.841	40	45630.31	−.002	28391	3–	74021	4
2128.492	20	46966.79	.003	46259	2–	93226	2	2190.980	100	45627.41	.000	51002	3–	96630	3
											−.004	30507	4–	76134	3
2128.687	20	46962.48	.000	39321	3–	86283	3								
2130.133	2h	46930.61	.001	47577	2–	94508	3	2192.405	80	45597.76	−.002	36904	5–	82502	4
2130.286	20	46927.24	−.002	33631	4–	80558	3	2193.043	900	45584.49	−.002	35429	6–	81013	6
2131.364	80	46903.51	.002	23317	5–	70221	4	2196.714	1	45508.32	.003	33631	4–	79139	3
2133.065	40	46866.11	−.001	29512	0–	76378	1	2197.831	100	45485.20	.001	26588	4–	72073	4
2133.228	20	46862.53	−.003	33631	4–	80493	5	2198.373	60	45473.99	.001	19632	5–	65106	4
2135.040	200	46822.76	.000	28977	4–	75800	5	2198.878	20	45463.54	−.001	41403	2–	86867	2
2139.779	2	46719.07	−.006	43378	4–	90097	3	2198.997	2	45461.08	.000	41403	2–	86865	1
2140.601	20	46701.13	−.001	26588	4–	73289	5	2200.261	40	45434.97	−.006	43378	4–	88813	3
2141.312	50	46685.63	.000	47181	4–	93867	4	2206.125	1H	45314.21	.008	56632	2–	101947	2
								2206.501	20	45306.49	−.002	36904	5–	82210	5
2207.057	10	45295.08	−.002	41570	1–	86865	1	2268.823	20	44062.09	−.001	52568	3–	96630	3
2207.450	30	45287.02	−.002	29855	1–	75142	0	2270.454	20	44030.44	−.003	23080	1–	67111	2
2209.052	80	45254.18	.000	19851	3–	65106	4	2271.830	20	44003.78	−.002	29800	2–	73804	1
2210.204	30	45230.59	−.001	47995	1–	93226	2	2272.386	5	43993.01	.000	52568	3–	96561	2
2212.122	5	45191.38	−.004	57384	3–	102576	2	2273.040	2	43980.36	−.002	47181	4–	91161	5
2213.103	20b	45171.35	−.005	37808	1–	82980	2	2277.184	1	43900.33	.005	41322	5–	85222	5
2216.427	30	45103.61	.008	45388	2–	90492	1	2277.387	lh	43896.41	.004	29800	2–	73697	3
2217.934	50	45072.97	−.004	28062	2–	73135	2	2280.425	80	43837.94	.005	46259	2–	90097	3
2218.224	20	45067.08	−.005	36226	3–	81293	2	2288.527	100	43682.76	−.005	28391	3–	72073	4
2218.332	100	45064.88	.000	35429	6–	80493	5	2289.311	50	43667.80	.000	42615	3–	86283	3
2218.979	1	45051.74	−.003	43378	4–	88429	3	2289.952	20	43655.58	.000	42615	3–	86271	4
2219.365	100	45043.91	.000	28977	4–	74021	4	2291.017	30	43635.28	−.002	39344	2–	82980	2
2221.055	1	45009.64	.000	31821	3–	76831	2	2291.146	30	43632.83	−.001	26588	4–	70221	4
2221.273	2	45005.22	.001	42615	3–	87621	2	2293.421	100	43589.55	−.002	36904	5–	80493	5
2221.883	20h	44992.87	.001	37808	1–	82801	2	2294.194	10	43574.86	−.004	16621	2–	60195	1
			−.003	31838	1–	76831	2	2295.686	40	43546.55	−.004	41322	5–	84869	4
2221.970	20h	44991.11	−.001	29800	2–	74791	2	2297.399	30	43514.08	.000	30507	4–	74021	4
2222.769	80b	44974.94	−.010	42615	3–	87590	4	2301.298	8	43440.36	−.003	33631	4–	77071	3
2223.354	20h	44963.10	.006	57384	3–	102348	3	2306.285	2	43346.44	.000	36353	2–	79699	3
			−.004	52568	3–	97531	3	2309.824	10b	43280.03	.003	29855	1–	73135	2
2224.049	80	44949.05	−.004	41322	5–	86271	4	2312.020	10	43238.92	−.001	13992	2–	57231	2
2224.682	40	44936.27	.000	47181	4–	92117	4	2312.365	2	43232.47	−.001	36467	4–	79699	3
			−.003	29855	1–	74791	2	2313.088	2	43218.96	.001	42615	3–	85834	3
2226.525	2b	44899.07	.002	22212	3–	67111	2	2314.094	40	43200.18	−.001	43378	4–	86578	3
2227.482	50	44879.79	.000	41403	2–	86283	3	2314.638	100	43190.02	.003	30507	4–	73697	3
			−.006	24097	1–	68976	1	2318.821	1	43112.12	−.008	18376	4–	61488	3
2228.100	30	44867.34	−.003	16621	2–	61488	3	2321.045	40	43070.81	−.002	46259	2–	89329	3
2230.393	40b	44821.22	.000	27252	3–	72073	4	2324.104	40	43014.13	−.004	24097	1–	67111	2
2230.522	1	44818.62	−.004	28062	2–	72881	3	2325.884	5	42981.21	.000	46259	2–	89240	2
2231.162	40	44805.77	.000	39321	3–	84127	3	2326.482	40	42970.16	.001	31821	3–	74791	2
											−.010	19851	3–	62821	2
2231.251	500	44803.98	−.002	12427	2–	57231	2								
2232.344	1	44782.05	.002	39344	2–	84127	3	2326.552	5	42968.87	−.003	27252	3–	70221	4
2232.597	10	44776.97	−.003	33631	4–	78408	4	2329.439	lh	42915.62	−.003	47181	4–	90097	3
2234.204	80	44744.77	−.001	28391	3–	73135	2	2329.559	5h	42913.41	.005	29512	0–	72425	1
2234.828	80	44732.28	.001	37769	4–	82502	4				−.003	36226	3–	79139	3
2236.007	10	44708.69	−.002	45388	2–	90097	3	2330.653	40h	42893.27	.000	43378	4–	86271	4
2237.688	1	44675.11	−.003	56632	2–	101307	1	2332.009	100	42868.33	.000	24490	2–	67358	1
2237.769	10	44673.49	.001	20432	4–	65106	4	2332.214	1	42864.56	−.002	51002	3–	93867	4
2239.296	100	44643.03	.001	41322	5–	85965	6	2332.302	80	42862.95	−.001	36353	2–	79216	1
2239.531	1	44638.35	.001	55564	4–	100202	4	2332.511	1	42859.11	−.003	33972	2–	76831	2
								2336.343	20	42788.82	.001	37769	4–	80558	3
2242.040	200	44588.40	.002	31211	5–	75800	5								
2242.660	40h	44576.07	.000	52568	3–	97144	4	2338.438	lh	42750.48	.000	39344	2–	82095	1
2243.351	1	44562.35	−.004	57384	3–	101947	2	2339.883	30h	42724.09	.001	37769	4–	80493	5
2244.499	5	44539.56	−.002	31838	1–	76378	1	2341.239	30	42699.34	−.002	28062	2–	70762	2
2246.982	100	44490.34	.003	28391	3–	72881	3	2342.713	20	42672.48	.002	36467	4–	79139	3
2247.229	10	44485.45	.000	31211	5–	75697	6	2344.803	20	42634.45	.002	41322	5–	83957	5
2247.319	40	44483.67	.005	43378	4–	87862	5	2345.677	200	42618.56	.006	28062	2–	70681	3
2250.573	2	44419.36	−.005	51002	3–	95422	3	2348.542	1	42566.58	−.002	52568	3–	95134	2
2250.836	1	44414.17	−.002	23317	5–	67731	5	2348.901	5	42560.07	−.003	36226	3–	78786	2
2251.358	100	44403.87	.000	22955	2–	67358	1	2351.151	5	42519.35	.002	47577	2–	90097	3
								2352.048	40	42503.13	.002	33631	4–	76134	3
2251.769	1	44395.77	.001	47577	2–	91973	2								
2254.089	60	44350.08	−.004	12881	1–	57231	2	2352.397	20	42496.83	.003	47995	1–	90492	1
2256.009	500	44312.34	−.006	28977	4–	73289	5	2354.624	50	42456.64	.003	43378	4–	85834	3
2257.124	40b	44290.45	−.002	39321	3–	83611	3	2355.026	40	42449.39	−.002	47181	4–	89630	5
2257.325	10	44286.51	−.001	37808	1–	82095	1	2355.930	5h	42433.10	.003	36353	2–	78786	2
2257.754	200	44278.09	.000	23080	1–	67358	1	2357.450	80	42405.74	.000	33972	2–	76378	1
2258.122	200	44270.88	−.004	23080	1–	67351	0	2359.209	100	42374.13	.006	30507	4–	72881	3
2258.335	40	44266.70	.002	39344	2–	83611	3	2360.257	2	42355.32	.001	51002	3–	93358	3
2259.201	1	44249.74	.002	39321	3–	83571	4	2360.977	1	42342.40	.004	50539	2–	92882	1
2260.051	30h	44233.10	.003	46259	2–	90492	1	2361.601	30	42331.21	.001	14899	3–	57231	2
								2365.974	20	42252.98	.004	42615	3–	84869	4
2261.105	40h	44212.48	−.002	43378	4–	87590	4								
2261.939	100	44196.18	.002	33631	4–	77827	4	2366.226	10	42248.48	.001	41322	5–	83571	4
2263.988	200	44156.18	−.001	22955	2–	67111	2	2367.115	1	42232.62	.002	45388	2–	87621	2
2264.980	2	44136.85	.001	52568	3–	96704	4	2368.516	10	42207.64	.005	41403	2–	83611	3
2265.237	1	44131.84	.002	51002	3–	95134	2	2368.974	300	42199.48	.006	31821	3–	74021	4
2265.679	10h	44123.23	−.010	41403	2–	85527	2	2370.706	100	42168.65	.007	33631	4=	75800	5
2266.404	80b	44109.12	−.001	36904	5–	81013	6	2375.827	200	42077.76	.008	31211	5–	73289	5
2266.459	60b	44108.05	−.004	17380	4–	61488	3	2381.811	200	41972.06	.006	39321	3–	81293	2
2267.229	1	44093.07	.001	26588	4–	70681	3	2383.577	lOh	41940.96	.006	36467	4–	78408	4
2267.316	60h	44091.38	−.002	36467	4–	80558	3	2387.301	40	41875.54	.003	31821	3–	73697	3
								2389.885	60	41830.27	−.001	28391	3–	70221	4
2390.016	80	41827.98	−.002	37769	4–	79597	4	2509.989	7b	39828.82	−.007	56732	1–	96561	2
2393.431	1	41768.30	−.009	25963	6–	67731	5	2510.636	1	39818.55	−.001	39321	3–	79139	3
2394.361	1	41752.08	.003	47577	2–	89329	3	2510.965	10	39813.34	.000	36226	3–	76039	2
2396.814	1	41709.35	.002	39344	2–	81054	1	2516.548	30	39725.02	.000	33972	2–	73697	3
2397.103	200	41704.32	−.008	28977	4–	70681	3	2517.245	80	39714.02	.004	30507	4–	70221	4
2401.296	2h	41631.51	−.002	47181	4–	88813	3	2518.715	200	39690.84	.005	41322	5–	81013	6
2407.134	2h	41530.54	.000	36226	3–	77757	2	2518.990	5	39686.51	−.001	36353	2–	76039	2
2408.679	20h	41503.91	.004	36904	5–	78408	4	2520.210	40	39667.30	.001	36467	4–	76134	3
2409.445	30H	41490.71	.003	43378	4–	84869	4	2520.785	10	39658.25	−.001	33631	4–	73289	5
2410.275	20H	41476.43	.004	45388	2–	86865	1	2522.412	30	39632.67	.001	27726	0–	67358	1
2414.508	1	41403.72	−.002	36353	2–	77757	2	2522.855	5	39625.71	.002	47995	1–	87621	2
2416.476	1h	41370.00	.002	37769	4–	79139	3	2527.697	2h	39549.81	.001	52568	3–	92117	4
2416.943	10H	41362.01	.001	46259	2–	87621	2	2531.889	1h	39484.33	−.001	41570	1–	81054	1
2419.745	40h	41314.12	−.003	31821	3–	73135	2	2533.142	1h	39464.80	−.001	29512	0–	68976	1
2420.733	l0h	41297.26	.000	31838	1–	73135	2	2534.636	2	39441.54	−.001	39344	2–	78786	2
2423.865	200	41243.90	.001	28977	4–	70221	4	2541.637	40	39332.91	.000	36467	4–	75800	5
2424.371	10	41235.29	−.001	47577	2–	88813	3	2542.471	1h	39320.01	.001	57384	3–	96704	4
2424.577	80	41231.79	.001	41570	1–	82801	2	2544.023	10	39296.02	−.004	28062	2–	67358	1
2427.652	30H	41179.56	−.001	41322	5–	82502	4	2545.851	2h	39267.81	.002	46259	2–	85527	2
2429.787	200	41143.38	.001	26588	4–	67731	5	2556.639	1h	39102.12	.000	47181	4–	86283	3
2432.441	1h	41098.50	.003	45388	2–	86487	2	2557.621	5	39087.11	−.002	39321	3–	78408	4
2439.619	1	40977.58	−.003	37808	1–	78786	2	2562.700	40	39009.65	−.004	31211	5–	70221	4
2440.963	1h	40955.02	.004	42615	3–	83571	4	2570.196	30	38895.88	−.004	36904	5–	75800	5
2443.422	1	40913.81	.002	28062	2–	68976	1	2572.580	80	38859.84	.002	31821	3–	70681	3
2444.530	l0h	40895.27	−.004	45388	2–	86283	3	2576.790	200	38796.36	−.005	28391	3–	67187	3
2444.943	100	40888.36	−.004	41322	5–	82210	5	2579.570	800	38754.55	−.003	28977	4–	67731	5
2448.866	1h	40822.86	−.006	50539	2–	91362	2	2582.810	1	38705.94	.000	47577	2–	86283	3
2453.317	l0h	40748.80	−.001	43378	4–	84127	3	2583.150	1	38700.84	−.003	50539	2–	89240	2
2456.333	1	40698.77	−.001	37808	1–	78507	0	2584.701	1h	38677.62	−.009	42615	3–	81293	2
2457.441	60	40680.42	−.002	47181	4–	87862	5	2591.968	30	38569.19	−.003	37808	1–	76378	1
2459.971	1h	40638.59	−.001	37769	4–	78408	4	2592.207	1	38565.63	−.010	36226	3–	74791	2
2462.380	l0h	40598.83	.000	26588	4–	67187	3	2595.442	1	38517.57	−.004	26588	4–	65106	4
2466.841	2H	40525.42	.000	41570	1–	82095	1	2600.523	50	38442.31	.000	33631	4–	72073	4
2471.661	10H	40446.40	−.001	45388	2–	85834	3	2603.413	l0h	38399.64	−.004	31821	3–	70221	4
2473.950	40h	40408.98	.005	47181	4–	87590	4	2605.775	40h	38364.84	−.001	37769	4–	76134	3
2475.116	2h	40389.94	−.001	33631	4–	74021	4	2608.050	40h	38331.37	−.007	24490	2–	62821	2
2476.264	5h	40371.22	−.002	35429	6–	75800	5	2614.441	2h	38237.68	−.002	51002	3–	89240	2
2482.091	60	40276.45	.001	39321	3–	79597	4	2616.059	2h	38214.03	.003	55564	4–	93778	5
2482.600	40	40268.19	.000	35429	6–	75697	6	2628.686	40	38030.48	−.004	37769	4–	75800	5
2485.091	1	40227.83	.006	46259	2–	86487	2	2645.068	2h	37794.95	−.005	36226	3–	74021	4
2487.257	5h	40192.80	−.001	43378	4–	83571	4	2652.621	1h	37687.34	.003	47181	4–	84869	4
2488.401	20h	40174.33	.002	30507	4–	70681	3	2662.033	2h	37554.10	−.003	36467	4–	74021	4
2495.133	2h	40065.94	−.001	33631	4–	73697	3	2666.162	l0h	37495.95	−.006	29855	1–	67351	0
2495.635	1h	40057.88	−.001	37769	4–	77827	4	2673.962	1	37386.58	−.005	29800	2–	67187	3
2497.706	1h	40024.67	.007	36353	2–	76378	1	2677.007	l0h	37344.05	−.001	36353	2–	73697	3
			−.006	46259	2–	86283	3	2677.742	1	37333.80	−.001	37808	1–	75142	0
2502.494	l0h	39948.10	−.002	37808	1–	77757	2								
2504.995	l0h	39908.21	−.005	36226	3–	76134	3								
2506.167	1	39889.55	−.006	41403	2–	81293	2								
2507.593	60	39866.87	−.003	22955	2–	62821	2								

a The symbols represent: b=blended; h=hazy; H=very hazy.

b The first named level is from the (5*d*^4^*+*5*d*^3^ 6*s+*5*d*^2^ 6*s*) even configurations and the second level is from the (5*d*^3^ 6*p+*5*d*6*s*6*p*) odd configurations.

**Table 2:** Unclassified lines attributed to W III

Wavelength (Å)	Int.	Wavenumber (cm^−1^)	Wavelength(Å)	Int	Wavenumber (cm^−1^)	Wavelength (Å)	Int.	Wavenumber (cm^−1^)
904.587	500b	110547.68	1257.980	10	79492.52	1317.767	50b	75885.94
910.063	400b	109882.50	1260.485	50b	79334.54	1318.014	3	75871.72
927.665	50	107797.53	1262.665	5	79197.57	1319.499	5b	75786.34
932.853	500	107198.02	1262.956	50	79179.32	1321.696	10	75660.36
932.933	5	107188.83	1263.328	20b	79156.00	1321.869	5	75650.46
935.040	50	106947.29	1263.594	20	79139.34	1324.615	3h	75493.63
937.234	15	106696.94	1265.526	10	79018.52	1324.816	10	75482.18
937.331	50	106685.89	1265.610	10	79013.28	1329.267	50	75229.43
1050.408	5	95201.10	1265.678	20	79009.03	1330.413	20	75164.62
1092.515	3	91531.92	1268.431	30b	78837.55	1330.832	5	75140.96
1108.732	3	90193.12	1268.480	60	78834.51	1331.121	5	75124.65
1109.972	5	90092.36	1269.392	20	78777.87	1331.319	20b	75113.47
1116.908	3	89532.88	1269.522	5b	78769.80	1331.518	50	75102.25
1119.663	10b	89312.58	1271.033	5	78676.16	1333.289	20	75002.49
1130.600	20	88448.61	1271.452	3h	78650.23	1333.844	50	74971.28
1134.317	10	88158.77	1272.611	5	78578.60	1334.962	3	74908.49
1135.834	3	88041.03	1272.810	20	78566.32	1337.325	3	74776.13
1142.652	2	87515.70	1274.263	10	78476.73	1338.360	5	74718.31
1143.909	10	87419.54	1275.324	20b	78411.44	1339.575	20	74650.54
1147.869	5b	87117.95	1275.769	3	78384.09	1339.964	50	74628.87
1152.146	50	86794.55	1277.642	5	78269.18	1340.246	3h	74613.16
1154.200	50	86640.09	1279.875	20	78132.63	1340.331	50	74608.43
1156.398	3	86475.41	1280.463	20	78096.75	1342.767	3	74473.08
1157.974	10	86357.72	1281.409	5	78039.09	1342.922	10	74464.48
1163.467	3	85950.01	1281.584	5	78028.44	1343.464	10	74434.44
1175.141	5b	85096.17	1281.968	5	78005.06	1344.692	10	74366.47
1175.712	5h	85054.84	1282.845	10	77951.74	1345.298	3	74332.97
1180.161	2	84734.20	1283.204	20	77929.93	1346.411	5	74271.52
1181.548	5	84634.73	1284.082	10	77876.64	1346.591	10	74261.59
1188.080	20	84169.41	1284.227	5	77867.85	1346.873	3h	74246.05
1190.214	2b	84018.50	1286.002	50	77760.37	1348.044	3	74181.55
1198.703	10	83423.50	1287.554	3	77666.64	1350.069	10	74070.28
1202.803	5b	83139.13	1288.055	5b	77636.43	1351.221	30	74007.13
1206.056	2b	82914.89	1288.277	20	77623.05	1352.492	3h	73937.59
1208.430	2	82752.00	1290.526	10	77487.78	1352.760	50	73922.94
1208.887	200	82720.71	1291.657	3	77419.93	1352.890	30b	73915.83
1209.120	2	82704.77	1291.965	3h	77401.47	1353.545	500b	73880.07
1209.742	3	82662.25	1292.660	20	77359.86	1353.886	40	73861.46
1211.844	2	82518.87	1294.445	3	77253.18	1354.519	200b	73826.94
1212.585	5	82468.44	1295.790	20	77172.99	1354.616	3h	73821.65
1216.554	3	82199.39	1296.681	10	77119.97	1354.677	3h	73818.33
1220.126	10	81958.74	1297.703	20	77059.23	1355.131	10b	73793.60
1222.763	2	81781.99	1298.560	20	77008.37	1355.219	30b	73788.81
1225.234	5b	81617.06	1299.604	3	76946.51	1355.304	80	73784.18
1225.281	10	81613.93	1299.636	3	76944.62	1355.654	10	73765.13
1225.939	3	81570.12	1300.087	3	76917.92	1357.009	5h	73691.47
1227.565	5	81462.08	1301.820	10	76815.53	1359.382	200	73562.84
1227.702	3	81452.99	1304.172	10	76677.00	1360.723	40	73490.34
1229.741	2	81317.93	1305.271	50	76612.44	1361.590	l0h	73443.54
1236.209	20	80892.47	1306.850	50	76519.87	1362.342	20b	73403.00
1236.982	20b	80841.92	1307.778	100	76465.57	1362.724	3h	73382.43
1237.787	2h	80789.34	1309.020	10	76393.02	1363.032	3h	73365.84
1237.917	10	80780.86	1309.521	100b	76363.80	1363.663	3	73331.90
1240.864	50b	80589.00	1309.691	50	76353.88	1366.103	20	73200.92
1242.018	5	80514.13	1310.538	5	76304.54	1367.343	20H	73134.53
1242.065	3h	80511.08	1310.825	10	76287.83	1368.054	20	73096.53
1242.308	50	80495.33	1310.887	10	76284.22	1368.266	50	73085.20
1243.177	100b	80439.06	1310.942	3	76281.02	1368.769	3	73058.34
1244.487	3	80354.39	1311.226	5	76264.50	1369.011	30	73045.43
1245.880	10	80264.55	1311.322	5	76258.92	1369.762	10	73005.38
1246.146	3	80247.41	1311.466	1000h	76250.54	1372.699	3	72849.18
1247.798	3	80141.17	1311.825	100	76229.68	1372.842	20	72841.59
1248.214	10	80114.46	1311.883	50b	76226.31	1373.372	50	72813.48
1249.357	lb	80041.17	1312.168	10	76209.75	1374.024	40	72778.93
1249.753	10b	80015.81	1312.281	200h	76203.19	1375.279	5	72712.51
1249.843	100	80010.04	1312.405	100b	76195.99	1375.492	10	72701.25
1253.092	10	79802.60	1312.585	20b	76185.54	1376.851	20b	72629.50
1254.193	3	79732.54	1313.884	10b	76110.22	1377.634	10	72588.22
1254.263	3	79728.09	1316.241	10	75973.92	1378.072	3h	72565.14
1254.711	3	79699.62	1317.688	5	75890.49	1378.224	10	72557.14
1378.466	10b	72544.40	1408.081	50	71018.64	1515.430	20	65987.87
1378.498	10b	72542.72	1408.846	10	70980.07	1515.741	25	65974.33
1378.837	10b	72524.88	1408.941	50	70975.29	1516.901	25	65923.88
1379.019	3	72515.31	1409.176	5h	70963.45	1517.213	5	65910.32
1379.288	10	72501.17	1409.232	1b	70960.63	1517.244	20	65908.97
1380.169	3h	72454.89	1409.413	20	70951.52	1520.530	15	65766.54
1380.355	5h	72445.13	1409.480	3	70948.15	1524.720	10	65585.81
1381.132	50b	72404.37	1409.541	50b	70945.08	1526.500	25	65509.33
1381.252	10	72398.08	1409.759	300h	70934.11	1527.469	30	65467.77
1381.635	3	72378.01	1409.994	50b	70922.28	1527.722	20	65456.93
1381.847	5b	72366.91	1410.151	3	70914.39	1529.068	35	65399.31
1382.334	30	72341.41	1410.410	5	70901.36	1529.272	4	65390.59
1382.512	5	72332.10	1410.497	10	70896.99	1530.009	45	65359.09
1382.558	3h	72329.69	1411.291	3	70857.10	1530.497	20	65338.25
1383.725	50	72268.69	1411.748	10	70834.17	1531.353	40	65301.73
1384.031	50b	72252.71	1412.855	30	70778.67	1531.413	30	65299.17
1384.795	3	72212.85	1413.504	5	70746.17	1531.775	5	65283.73
1385.155	50	72194.08	1414.155	10	70713.60	1531.907	100	65278.11
1385.270	3h	72188.09	1415.192	100	70661.79	1533.276	10	65219.83
1385.336	50	72184.65	1415.583	3b	70642.27	1533.690	4	65202.22
1385.728	10b	72164.23	1416.347	5	70604.16	1536.415	40	65086.58
1385.817	80	72159.60	1416.647	50	70589.21	1537.588	25	65036.92
1386.775	3b	72109.75	1416.801	50	70581.54	1537.886	5	65024.32
1388.327	5	72029.14	1416.874	30	70577.90	1538.285	4	65007.46
1388.414	20	72024.62	1417.014	5b	70570.93	1538.841	15	64983.97
1388.491	10	72020.63	1417.073	200h	70567.99	1538.935	5	64980.00
1388.787	3	72005.28	1419.650	50	70439.89	1539.994	150	64935.31
1389.023	5	71993.04	1421.466	4	70349.90	1540.066	5	64932.28
1389.339	20	71976.67	1421.913	40	70327.79	1540.324	5	64921.40
1389.827	5b	71951.40	1427.833	40	70036.20	1540.743	5	64903.75
1390.692	3h	71906.64	1427.975	50	70029.23	1541.534	50	64870.44
1390.963	5	71892.63	1429.808	15	69939.46	1541.968	20	64852.18
1391.399	100	71870.11	1444.152	10	69244.78	1542.413	10	64833.47
1391.457	100	71867.11	1459.031	10	68538.64	1542.698	4	64821.50
1392.500	40b	71813.28	1459.974	5	68494.37	1543.711	5	64778.96
1393.250	80	71774.62	1462.593	4	68371.72	1543.841	4	64773.51
1393.457	10	71763.96	1463.104	5	68347.84	1544.914	4	64728.52
1393.519	50b	71760.77	1463.771	4	68316.69	1545.662	50	64697.19
1393.659	30	71753.56	1464.100	4	68301.34	1546.078	25	64679.79
1393.846	20	71743.93	1464.153	5	68298.87	1551.425	50	64456.87
1394.125	5	71729.57	1464.809	15	68268.28	1556.602	100	64242.49
1395.288	50	71669.79	1465.239	100	68248.25	1558.247	150	64174.67
1395.395	10b	71664.29	1465.346	4	68243.26	1559.227	35	64134.34
1396.165	5	71624.77	1466.218	100	68202.68	1560.791	10	64070.07
1396.828	5h	71590.77	1467.939	200	68122.72	1561.388	200	64045.58
1398.099	30	71525.69	1469.210	5	68063.78	1562.078	20	64017.29
1398.905	5	71484.48	1469.756	5	68038.50	1563.546	15	63957.18
1399.195	3	71469.66	1470.495	25	68004.31	1564.794	50	63906.17
1399.723	50	71442.70	1476.649	4	67720.90	1565.369	50	63882.70
1400.244	3	71416.12	1477.936	5	67661.92	1568.000	10	63775.51
1400.693	5h	71393.23	1481.093	5	67517.70	1569.604	15	63710.33
1401.452	3b	71354.56	1482.676	5	67445.61	1569.721	10	63705.58
1402.112	50	71320.97	1484.908	10	67344.24	1570.408	5	63677.71
1402.557	5	71298.35	1487.473	50	67228.11	1570.503	10	63673.86
1402.643	3H	71293.97	1494.297	40	66921.10	1570.727	15	63664.78
1403.751	40	71237.70	1497.644	200	66771.54	1573.273	35	63561.75
1404.113	10	71219.33	1499.512	4	66688.36	1574.803	30	63500.00
1404.178	10	71216.04	1499.580	25	66685.33	1574.973	5	63493.15
1404.457	3	71201.89	1500.304	5	66653.15	1575.217	15	63483.31
1404.809	3	71184.05	1501.264	5	66610.53	1575.329	5	63478.80
1404.898	3	71179.54	1501.596	5	66595.80	1575.481	5	63472.67
1405.342	10	71157.05	1502.228	5	66567.79	1576.200	5	63443.72
1405.629	5	71142.52	1503.492	10	66511.82	1576.309	10	63439.33
1406.256	5	71110.80	1504.758	4	66455.86	1576.768	4	63420.87
1406.515	50	71097.71	1504.829	5	66452.73	1577.382	4	63396.18
1406.864	50	71080.07	1505.621	50	66417.77	1577.758	5	63381.07
1407.118	30	71067.24	1506.255	4	66389.82	1578.237	4	63361.84
1407.561	5	71044.87	1506.895	15	66361.62	1578.566	25	63348.63
1407.644	3	71040.68	1509.998	50	66225.25	1578.668	4	63344.54
1408.025	20	71021.46	1513.369	10	66077.73	1579.242	5	63321.51
1580.032	50	63289.85	1631.247	45	61302.79	1770.733	2	56473.78
1580.123	200	63286.21	1632.750	15	61246.36	1770.950	10	56466.86
1580.377	4	63276.04	1633.367	20	61223.22	1771.053	2	56463.58
1580.920	5	63254.30	1633.429	50	61220.90	1771.514	2	56448.89
1582.027	5	63210.04	1633.566	15	61215.77	1771.599	2	56446.18
1582.060	4	63208.72	1634.216	5	61191.42	1771.631	2	56445.16
1582.700	300	63183.16	1635.900	5	61128.43	1771.678	15	56443.66
1583.243	45	63161.49	1640.699	20	60949.63	1771.712	5	56442.58
1583.852	4	63137.21	1645.501	5	60771.76	1773.382	20	56389.43
1584.980	4	63092.27	1646.208	10	60745.66	1775.053	3	56336.34
1585.761	5	63061.20	1647.738	4	60689.26	1775.220	3	56331.04
1588.278	50	62961.27	1648.183	5	60672.87	1777.009	5b	56274.33
1588.355	5	62958.21	1649.883	4	60610.35	1777.130	40	56270.50
1590.772	10	62862.56	1650.349	5	60593.24	1777.509	20	56258.50
1592.320	4	62801.44	1650.869	20	60574.15	1779.054	20	56209.64
1592.634	25	62789.06	1652.016	4	60532.10	1779.701	2	56189.21
1593.035	5	62773.26	1656.861	5	60355.09	1779.881	20	56183.53
1593.579	10	62751.83	1659.065	5	60274.91	1782.322	5	56106.58
1593.752	15	62745.01	1659.432	25	60261.58	1782.579	5b	56098.49
1595.471	35	62677.41	1661.405	10	60190.02	1784.075	5	56051.45
1596.460	5	62638.58	1662.098	10	60164.92	1784.271	20b	56045.29
1597.177	5	62610.46	1663.280	35	60122.16	1785.444	40	56008.47
1597.404	5	62601.57	1663.725	10	60106.08	1786.135	2	55986.81
1597.644	5	62592.16	1666.225	30	60015.90	1786.471	20	55976.27
1597.850	4	62584.09	1671.777	4	59816.59	1787.150	2	55955.01
1598.100	10	62574.30	1673.498	10	59755.07	1787.977	5	55929.13
1598.316	5	62565.85	1673.615	5	59750.89	1788.851	2	55901.80
1598.425	10	62561.58	1678.451	5	59578.74	1789.789	10	55872.50
1598.778	5	62547.77	1678.862	15	59564.15	1790.688	5	55844.45
1598.894	10	62543.23	1679.738	5	59533.09	1791.175	5	55829.27
1599.983	10	62500.66	1681.221	5	59480.58	1792.843	2	55777.33
1600.193	5	62492.46	1682.536	5	59434.09	1794.345	2	55730.64
1600.475	5	62481.45	1687.180	5	59270.49	1794.963	2	55711.45
1600.591	5	62476.92	1689.575	15	59186.48	1795.534	5	55693.73
1600.921	10	62464.04	1689.841	15	59177.16	1796.166	2	55674.14
1601.269	5	62450.46	1689.915	10	59174.57	1796.491	5	55664.07
1601.629	4	62436.43	1696.157	10	58956.80	1796.550	8	55662.24
1602.005	5	62421.77	1702.391	4	58740.91	1798.601	2	55598.76
1602.402	5	62406.31	1702.988	5	58720.32	1799.148	20	55581.86
1602.709	50	62394.35	1704.396	15	58671.81	1799.549	5	55569.47
1603.305	5	62371.16	1717.949	5	58208.94	1799.871	2	55559.53
1603.666	5	62357.12	1739.457	10	57489.20	1800.380	2	55543.83
1603.929	20	62346.89	1744.218	15	57332.28	1800.458	10	55541.42
1604.142	10	62338.62	1749.308	10	57165.46	1800.924	2	55527.05
1604.270	20	62333.64	1750.995	10	57110.38	1802.595	5	55475.57
1605.112	4	62300.94	1751.034	2	57109.11	1803.975	2	55433.14
1605.404	5	62289.61	1751.740	20	57086.09	1804.521	20	55416.36
1606.468	50	62248.36	1751.973	80	57078.50	1808.149	5	55305.17
1606.568	15	62244.48	1752.107	10	57074.14	1808.499	30	55294.47
1610.088	25	62108.40	1753.191	20	57038.85	1808.653	2	55289.76
1610.151	150	62105.97	1753.561	100	57026.81	1809.585	2	55261.28
1611.776	50	62043.36	1753.662	20	57023.53	1810.011	2	55248.28
1612.811	4	62003.54	1754.273	5	57003.67	1811.914	20	55190.25
1613.749	50	61967.50	1754.451	20	56997.88	1812.182	100	55182.09
1614.490	5	61939.06	1755.004	30b	56979.92	1812.967	10	55158.20
1616.091	35	61877.70	1755.328	30	56969.41	1813.737	40	55134.78
1616.264	5	61871.08	1757.646	2	56894.27	1813.864	5	55130.92
1616.602	4	61858.14	1758.842	2	56855.59	1814.197	10	55120.80
1616.737	5	61852.97	1759.434	5	56836.46	1814.628	2	55107.71
1616.816	15	61849.95	1760.057	10	56816.34	1815.028	10	55095.56
1617.007	45	61842.65	1761.450	10	56771.41	1815.692	30	55075.42
1620.821	10	61697.12	1762.565	20b	56735.49	1817.137	10	55031.62
1621.458	5	61672.88	1762.575	30	56735.17	1817.342	20b	55025.41
1625.768	25	61509.39	1763.967	30	56690.40	1819.491	2	54960.42
1625.968	5	61501.82	1766.292	80	56615.78	1820.241	2	54937.78
1628.221	10	61416.72	1766.671	5b	56603.63	1820.577	20	54927.64
1629.339	5	61374.58	1767.151	3	56588.26	1821.415	5	54902.37
1629.608	5	61364.45	1768.678	10	56539.40	1821.839	3	54889.59
1629.769	35	61358.38	1769.346	15	56518.05	1822.963	5	54855.74
1630.621	10	61326.32	1769.372	2	56517.22	1824.256	20	54816.86
1824.713	5	54803.13	1888.427	10	52954.12	1965.183	5	50885.84
1825.547	5	54778.10	1888.701	20	52946.44	1965.421	50	50879.68
1825.676	5	54774.23	1889.446	10	52925.56	1966.615	5	50848.79
1825.547	10	54778.10	1889.978	5	52910.66	1969.858	2	50765.08
1825.675	8	54774.26	1890.299	2	52901.68	1971.067	5	50733.94
1826.043	8	54763.22	1890.690	2	52890.74	1971.155	2	50731.67
1826.229	2	54757.64	1892.429	40	52842.14	1971.625	2	50719.58
1827.264	40	54726.63	1893.043	2	52825.00	1972.498	2	50697.13
1827.386	10	54722.97	1893.681	2b	52807.20	1973.739	3	50665.26
1828.344	10	54694.30	1893.873	2	52801.85	1973.979	5	50659.10
1830.167	5	54639.82	1897.800	8	52692.59	1976.016	3	50606.87
1830.210	8	54638.53	1899.116	5	52656.07	1976.354	5	50598.22
1830.327	5	54635.04	1901.403	2	52592.74	1978.726	10b	50537.56
1830.940	3	54616.75	1902.405	10	52565.04	1979.215	10	50525.08
1831.113	2	54611.59	1902.461	5b	52563.49	1981.160	60	50475.47
1837.402	20	54424.67	1902.863	5	52552.39	1981.404	30	50469.26
1838.629	2	54388.35	1903.229	8	52542.28	1982.359	40	50444.95
1840.641	10	54328.90	1906.011	2	52465.59	1984.000	10	50403.22
1841.556	30	54301.90	1906.827	60b	52443.14	1985.536	15b	50364.23
1842.400	80	54277.03	1908.166	30	52406.34	1985.550	20b	50363.87
1842.803	5	54265.16	1908.718	23b	52391.18	1986.297	10	50344.93
1843.375	2b	54248.32	1908.742	30	52390.52	1986.811	2	50331.91
1844.166	40	54225.05	1909.788	5	52361.83	1987.738	30	50308.44
1844.883	2	54203.97	1910.331	2	52346.94	1989.729	20	50258.10
1845.701	20	54179.95	1911.357	2	52318.85	1993.140	5	50172.09
1846.373	20	54160.23	1912.324	5	52292.39	1994.202	50	50145.37
1847.712	2	54120.98	1913.576	10	52258.18	1996.842	60	50079.07
1848.301	20	54103.74	1915.096	5	52216.70	1997.091	5	50072.83
1849.296	2	54074.63	1915.902	2	52194.73	1997.216	40b	50069.69
1849.574	5	54066.50	1916.382	30	52181.66	1997.917	2b	50052.12
1850.750	10b	54032.14	1917.730	2	52144.98	1998.001	3	50050.02
1851.899	5	53998.62	1919.147	3	52106.48	1999.290	60	50017.75
1854.388	50	53926.14	1920.193	20	52078.09	2001.757	80H	49939.93
1854.850	10	53912.71	1920.607	5	52066.87	2014.431	10	49625.78
1854.969	30	53909.25	1921.201	30	52050.77	2015.780	60	49592.57
1858.004	2	53821.19	1921.953	5	52030.40	2016.801	2h	49567.47
1858.273	3	53813.40	1923.122	2	51998.78	2017.238	40h	49556.73
1858.458	20	53808.04	1924.592	5	51959.06	2017.407	10	49552.58
1858.824	10	53797.45	1927.370	2	51884.17	2017.829	5	49542.22
1858.857	20	53796.50	1927.471	2b	51881.45	2022.513	5	49427.50
1860.029	10	53762.60	1927.899	2	51869.93	2027.032	40	49317.32
1860.421	2b	53751.27	1928.063	5	51865.52	2027.305	5H	49310.68
1860.705	2	53743.07	1928.584	40	51851.51	2033.243	2	49166.69
1862.728	5	53684.70	1929.295	20b	51832.40	2033.974	5	49149.03
1863.070	5	53674.84	1929.312	30b	51831.94	2037.929	2H	49053.66
1863.360	30	53666.49	1931.341	40	51777.49	2039.285	20	49021.04
1863.456	50	53663.73	1932.331	2	51750.96	2042.190	5	48951.32
1863.541	2	53661.28	1933.427	40	51721.63	2043.545	40	48918.87
1863.630	5	53658.72	1934.338	60	51697.27	2047.096	20	48834.02
1864.275	2	53640.15	1934.600	2	51690.27	2049.503	5	48776.68
1865.205	2	53613.41	1935.403	50	51668.82	2054.510	30	48657.82
1867.685	20	53542.21	1935.753	2	51659.48	2054.688	50	48653.61
1868.972	40	53505.34	1937.850	2	51603.58	2055.257	10	48640.14
1869.864	20	53479.82	1938.059	40	51598.01	2056.845	3	48602.59
1870.359	15	53465.67	1940.371	30	51536.53	2057.921	10	48577.18
1870.417	2	53464.01	1941.301	20	51511.84	2059.870	2	48531.22
1871.889	10	53421.97	1943.085	3	51464.55	2062.679	2	48465.14
1872.183	10	53413.58	1944.631	20	51423.63	2064.744	2h	48416.68
1873.346	2	53380.42	1946.818	5	51365.87	2068.650	5	48325.27
1879.927	10	53193.55	1947.686	3	51342.97	2073.877	5	48203.49
1882.247	20	53127.99	1949.092	2b	51305.94	2075.597	30	48163.55
1882.879	20	53110.15	1949.544	60	51294.04	2075.962	2	48155.08
1883.998	5	53078.61	1950.258	2	51275.26	2077.013	10	48130.72
1884.065	2	53076.72	1950.929	3	51257.63	2079.123	100	48081.88
1884.308	10	53069.88	1951.407	30	51245.07	2086.433	5	47913.44
1884.677	20	53059.49	1952.528	5	51215.65	2087.479	3	47889.43
1885.557	5	53034.72	1956.035	5b	51123.83	2087.712	2	47884.09
1885.812	2h	53027.55	1957.361	2	51089.19	2088.214	50	47872.58
1886.391	5b	53011.27	1958.551	5	51058.15	2089.758	20b	47837.21
1887.870	5	52969.74	1963.659	2	50925.33	2090.188	10	47827.37
2092.631	2h	47771.54	2184.174	2h	45769.57			
2094.758	100b	47723.04	2187.282	80b	45704.54	2384.822	10	41919.06
2095.601	10	47703.85	2187.457	2	45700.88	2385.198	5h	41912.46
2096.915	2	47673.96	2189.301	2h	45662.40	2389.791	2	41831.91
2105.763	20	47473.67	2189.742	2	45653.20	2390.378	40	41821.64
2106.137	5	47465.24	2203.149	10	45375.41	2390.882	5	41812.82
2107.929	l0h	47424.89	2207.922	2h	45277.33	2406.102	10H	41548.35
2109.494	2h	47389.71	2213.065	5b	45172.12	2409.463	20H	41490.40
2111.380	10	47347.39	2213.697	20	45159.23	2410.423	5	41473.88
2114.890	2	47268.81	2214.239	2	45148.17	2419.355	20	41320.77
						2420.502	5	41301.19
2116.133	2h	47241.05	2215.252	2	45127.53			
2116.335	5h	47236.54	2215.346	5	45125.61	2421.010	10	41292.53
2124.984	40h	47044.31	2218.163	5b	45068.31	2424.668	2h	41230.23
2128.825	2h	46959.44	2222.584	5	44978.67	2446.390	40	40864.17
2129.737	20h	46939.33	2224.193	80b	44946.14	2462.021	5h	40604.75
2137.183	5h	46775.81	2226.783	30	44893.87	2475.589	l0h	40382.22
2139.310	200	46729.31	2227.330	2	44882.84	2478.567	2h	40333.71
2140.027	2H	46713.65	2228.909	2	44851.05	2480.002	50	40310.37
2144.613	30	46613.77	2231.953	2	44789.89	2491.577	2h	40123.11
2146.370	10H	46575.62	2235.357	30	44721.69	2492.934	30	40101.28
						2499.230	5H	40000.26
2146.672	2	46569.07	2239.775	20	44633.48			
2151.596	20	46462.51	2247.686	5	44476.40	2546.285	2	39261.11
2152.540	2	46442.13	2249.348	5h	44443.54	2551.358	2b	39183.05
2153.072	2	46430.66	2249.896	100	44432.72	2552.349	5	39167.84
2155.250	2h	46383.74	2251.924	2h	44392.71	2557.930	2	39082.38
2156.426	30	46358.45	2257.221	2h	44288.54	2594.231	30H	38535.54
2156.942	2h	46347.36	2267.592	2	44086.01	2615.448	20	38222.95
2161.868	2h	46241.77	2273.173	l0h	43977.78	2616.328	2h	38210.10
2162.916	30h	46219.36	2283.338	30	43782.01	2620.204	20	38153.58
2164.479	10	46185.99	2283.979	5	43769.73	2626.244	2h	38065.83
						2632.206	2h	37979.62
2169.375	20h	46081.77	2284.612	5	43757.60			
2169.947	20	46069.62	2287.760	5	43697.40	2634.654	10h	37944.33
2170.234	30h	46063.53	2298.648	5	43490.43	2647.711	5	37757.22
2171.543	2b	46035.76	2307.661	5	43320.59	2658.824	20	37599.42
2171.764	2	46031.08	2318.892	30	43110.79			
2178.906	2b	45880.22	2322.621	2	43041.58			
2180.942	5	45837.39	2323.114	20	43032.45			
2181.869	20h	45817.92	2343.473	10b	42658.64			
2182.086	10b	45813.36	2343.501	20b	42658.13			
2183.783	2	45777.76	2352.863	10	42488.41			

a The symbols represent: b=blended; h=hazy; H=very hazy.

**Table 3:** Predicted terms in *LS*-coupling scheme for the low configurations of W III

Even configurations: 5*d*^4^ 5*d*^3^ 6*s*, 5*d*^2^ 6*S*^2^														
			*d*^4^				*d*^3^()*s*				*d*^2^*s*^2^			
		^5^D					(^4^F)	^5^F	^3^F					
	^3^P	^3^D	^3^F	^3^G	^3^H		(^4^P)	^5^P	^3^P		^3^P		^3^F	
	^3^P		^3^F				(^2^G)	^3^G	^1^G	^1^s		^1^D		^1^G
^1^S		^1^D	F	^1^G		^1^I	(^2^D)	^3^D	^1^D					
^1^S		^1^D		^1^G			(^2^P)	^3^P	^1^P					
							(^2^H)	^3^H	^1^H					
							(^2^F)	^3^F	^1^F					
							(^2^D)	^3^D	^1^D					

Odd configurations: 5*d*^3^ 6*p*, 5*d*^2^ 6*s*6*p*								
	Terms					Terms		
*d*^3^()*P*				*d*^2^()	*sp*()			
(^4^F)	^5^(DFG)	^3^(DFG)		(^3^F)	(^3^P)	^5^(DFG)	^3^(DFG)	^1^(DFG)
(^4^P)	^5^(SPD)	^3^(SPD)		(^3^F)	('P)		^3^(DFG)	
(^2^*G*)	^3^(FGH)	^1^(FGH)		(^3^P)	(^3^P)	^5^(SPD)	^3^(SPD)	^1^(SPD)
(^2^D)	^3^(PDF)	^1^(PDF)		(^3^P)	(^1^P)		^3^(SPD)	
(^2^p)	^3^(SPD)	^1^(SPD)		(^1^G)	(^3^P)		^3^(FGH)	
(^2^H)	^3^(GHI)	^1^(GHI)		(^1^G)	(^1^p)			^1^(FGH)
(^2^F)	^3^(DFG)	^1^(DFG)		(^1^D)	(^3^P)		^3^(PDF)	
(^2^D)	^3^(PDF)	^1^(PDF)		(^1^D)	(^1^P)			^1^(PDF)
				(^1^S)	(^3^P)		^3^P	
				(^1^S)	(^1^P)			^1^P

**Table 4:** Observed even levels of W III (5*d*^4^, 5*d*^3^()6*s*, 5*d*^2^ 6*s*^2*^)

Configuration	Term	*J*	Obs. Level (cm^−1^)	Unc.	No.	O-C	Leading percentages		
5*d*^4^	^5^D	0	0.00	.11	8	55.	64 ^5^D	15 ^3^P1	15 ^3^P2
		1	2256.20	.07	24	0.	83 ^5^D	8 ^3^P1	7 ^3^P2
		2	4461.19	.05	31	−22.	93 ^5^D		
		3	6277.81	.05	42	11.	91 ^5^D		
		4	7686.68	.05	36	91.	78 ^5^D	12 ^3^F2	5 ^3^F1
5*d*^4^	^3^P2	0	9904.30	.07	14	−123.	35 ^5^D	30 ^3^P2	17 ^3^P1
5*d*^3^(^4^F)6*s*	^5^F	1	10968.54	.04	34	21.	82 (^4^F)^5^F	5 (^2^D2)^3^D	
		2	12427.09	.03	48	−33.	79 (^4^F)^5^F		
5*d*^4^	^3^P2	1	12881.03	.04	40	−78.	38 ^3^P2	18 ^3^P1	13 ^5^D
5*d*^4^	^3^H	4	13700.95	.05	42	−46.	52 ^3^H	16 ^3^G	10 ^5^d
5*d*^4^		2	13992.14	.04	51	15.	40 ^3^F2	13 ^3^F1	12 ^1^D2
		3	14899.80	.03	49	17.	37 (^4^F)^5^F	27 ^3^G	12 ^3^F2
5*d*^3^(^4^F)6*s*	^5^F	3	15038.04	.03	56	−70.	56 (^4^F)^5^F	14 ^3^F2	13 ^3^G
5*d*^4^	^3^P2	2	16621.08	.03	49	−29.	36 ^3^P2	16 ^3^P1	13 (^2^P)^3^P
5*d*^4^	^3^H	5	16723.24	.05	33	−24.	73 ^3^H	22 ^3^G	
5*d*^3^(^4^F)6*s*	^5^F	4	17380.40	.03	53	−17.	73 (^4^F)^5^F	8 ^3^H	8 (^2^G)^3^G
		4	18376.40	.04	46	−15.	27 ^3^F2	24 ^3^H	17 (^4^F)^5^F
5*d*^4^	^3^H	6	18380.90	.06	18	57.	81 ^3^H	17 ^1^I	
5*d*^3^(^4^F)6*s*	^5^F	5	19632.05	.04	30	−26.	69 (^4^F)^5^F	15 (^2^G)^3^G	8 ^3^H
5*d*^4^		3	19851.87	.03	54	−19.	36 ^3^F2	34 ^3^G	8 (^4^F)^3^F
5*d*^4^	^3^G	4	20432.53	.04	42	−9.	46 ^3^G	18 ^1^G2	17 ^3^F2
5*d*^4^		2	20696.62	.03	49	−22.	22 ^3^D	18 ^1^D2	11 ^3^F2
5*d*^4^	^3^D	3	22212.08	.03	57	46.	64 ^3^D	8 (^2^D2)^3^D	8 ^3^G
5*d*^3^(^4^F)6*s*	^5^P	2	22955.03	.03	59	−17.	38 (^4^P)^5^P	16 ^3^P2	9 (^2^P)^3^P
		1	23080.82	.03	46	−27.	76 (^4^P)^5^P	14 (^2^P)^3^P	
5*d*^4^	^3^G	5	23317.80	.04	36	2.	57 ^3^G	22 (^4^F)^3^F	14 ^3^H
5*d*^4^	^3^D	1	24097.14	.04	32	−62.	70 ^3^D	15 (^2^D2)^3^D	
		2	24490.58	.03	50	−57.	19 (^4^F)^3^F	17 (^4^P)^5^P	16 ^3^D
5*d*^4^	^1^I	6	25963.79	.08	10	44.	83 ^1^I	16 ^3^H	
		4	26588.54	.03	47	128.	37 ^1^G2	24 (^2^G)^1^G	11 (^4^F)^3^F
5*d*^3^(^4^F)6*s*	^5^P	3	27252.53	.02	67	51.	95 (^4^P)^5^P		
5*d*^4^	^1^S	0	27726.22	.05	13	139.	53 ^1^S	17 ^1^S2	14 (^2^P)^3^P
5*d*^3^(^4^F)6*s*	^3^F	2	28062.95	.03	54	−10.	48 (^4^F)^3^F	22 ^3^D	9 ^1^D2
5*d*^3^(^4^F)6*s*	^3^G	3	28391.09	.03	60	26.	45 (^2^G)^3^G	33 (^4^F)^3^F	6 ^3^F1
		4	28977.44	.03	54	88.	39 (^2^H)^3^H	25 (^2^G)^3^G	9^1^G1
5*d*^4^	^1^F	3	29245.28	.04	46	−33.	60 ^1^F	12 (^2^F)^1^F	11 ^3^D
5*d*^3^(^2^F)6*s*	^3^P	0	29512.01	.04	17	31.	55 (^2^P)^3^P	21 ^3^P2	10 ^3^P*
		2	29800.86	.03	59	25.	28 (^4^P)^5^P	23 ^3^P1	15 (^2^P)^3^P
		1	29855.75	.03	33	39.	33 (^2^P)^3^P	20 (^4^P)^5^P	13 ^3^P2
		4	30507.27	.03	52	−180.	35 (^2^G)^3^G	21 ^1^G1	18 (^2^G)^1^G
		5	31211.76	.03	33	−53.	42 (^2^H)^3^H	30 (^2^G)^3^G	12 ^3^G
		3	31821.76	.02	58	71.	35 (^2^G)^3^G	34 (^4^F)^3^F	10 ^3^F1
		1	31838.58	.03	32	74.	23 (^2^P)^1^P	22 (^2^D2)^3^D	16 ^3^D
		4	33631.43	.02	53	114.	27 (^2^H)^3^H	23 ^3^F1	22 (^4^F)F
		2	33972.34	.03	52	−48.	17 ^3^F2	17 (^2^D2)^3^D	14 ^3^F1
5*d*^3^(^2^H)6*s*	^3^H	6	35429.01	.04	17	−99.	97 (^2^H)^3^H		
		3	36226.46	.03	56	−92.	22 ^3^F2	19 ^3^F1	19 (^2^D2)^3^D
		2	36353.31	.03	44	0.	16 ^3^F2	15 ^3^F*	13 (^2^D2)^3^D
		4	36467.29	.03	45	6.	28 ^3^F1	18 (^2^H)^3^H	15 ^3^F2
5*d*^3^(^2^H)6*s*	^3^H	5	36904.37	.03	31	65.	52 (^2^H)^3^H	38 (^3^G)^3^G	
		4	37769.78	.02	49	94.	36 (^4^F)^3^F	16 ^1^Gl	12 (^2^G)^3^G
		1	37808.94	.03	29	−7.	33 ^3^P1	29 (^2^P)^3^P	16 (^4^P)^3^P
		3	39321.27	.03	48	60.	52 (^2^D2)^3^D	11 ^3^D	9 (^4^F)^3^F
		2	39344.94	.03	45	−28.	20 (^2^D2)^3^D	17 (^2^P)^3^P	16 ^3^P1
5*d*^3^(^2^H)6*s*	^1^H	5	41322.55	.03	27	47.	88 (^2^H)^1^H	11 (^2^G)^3^G	
5*d*^3^(^2^F)6*s*	^3^F	2	41403.97	.03	39	−67.	39 (^2^F)^3^F	18 ^3^F1	11 ^3^F*
5*d*^3^(^2^P)6*s*	1P	1	41570.01	.03	23	21.	50 (^2^P)^1^P	14 (^2^D2)^3^D	11 ^3^P2
5*d*^3^(^2^F)6*s*	^3^F	3	42615.95	.03	40	3.	67 (^2^F)^3^F	22 ^3^F1	
		4	43378.25	.03	37	29.	67 (^2^F)^3^F	13 ^3^F1	
		2	45388.55	.03	28	18.	19 (^2^F)^3^F	17 (^2^D2)^3^D	13 (^2^Dl)^3^D
5*d*^3^(^4^P)6s	^3^P	2	46259.17	.03	23	−64.	54 (^4^P)^3^P	20 ^3^P2	5 ^3^F*
		4	47181.63	.03	30	−184.	34 (^2^G)^1^G	31 ^1^G2	22 ^1^Gl
		2	47577.82	.03	24	−60.	28 ^1^Dl	15 ^3^F*	13 ^1^D*
		1	47955.45	.04	12	−243.	44 (^4^P)^3^p	20 ^3^P2	18 (^2^P)^1^P
		2	50539.59	.04	20	68.	31 ^3^F*	19 ^1^Dl	15 ^1^D2
		3	51992.73	.03	25	−62.	43 ^3^F*	21 (^2^F)^1^F	15 (^2^D1)^3^D
		3	52568.07	.03	23	35.	39 (^3^F)^1^F	27 ^3^F*	11 ^1^F
5*d*^2^6*s*^2^	F	4	55564.60	.06	9	29.	79 ^3^F*	9 (^2^F)^3^F	8 ^3^F1
5*d*^3^(^2^D1)6*s*	^3^D	2	56632.80	.05	14	69.	66 (^2^Dl)^3^D	20 (^2^D2)^3^D	
		1	56732.38	.06	7	21.	65 (^2^Dl)^3^D	32 (^2^D2)^3^D	
		3	57384.91	.04	15	15.	60 (^2^Dl)^3^D	22 (^2^F)^1^F	8 (^2^D2)^3^D

**Table 5:** Observed odd levels of W III (*5d*^3^()6*p*, 5*d*^2^ 6*s*6*p*())

*J*	Obs. Level (cm^−1^)	Unc.	No.	O-C		Leading percentages	
2	57231.04	.05	7	−432.	72 (^4^F)^5^G	9 (^4^F)^3^F	8 (^2^D2)^3^F
1	60195.86	.05	9	−30.	46 (^4^F)^3^F	34 (^4^F)^3^D	
3	61488.36	.04	9	−183.	83 (^4^F)^5^G	6 (^4^F)^3^F	
2	62821.85	.05	10	−277.	42 (^4^F)^5^D	27 (^4^F)^3^D	10 (^4^F)^5^D
4	65106.05	.04	10	−124.	70 (^4^F)^3^G	7 (^4^F)^5^F	7 (^4^F)^3^G
2	67111.20	.04	14	−251.	34 (^4^F)^5^F	11 (^2^P)^3^D	10 (^4^F)^3^G
3	67187.37	.04	14	415.	50 (^4^F)^5^F	17 (^4^F)^5^D	12 (^4^F)^3^D
0	67351.62	.10	3	−555.	65 (^4^F)^5^D	11 (^2^P)^1^S	10 (^2^D2)^3^P
1	67358.91	.04	15	2.	25 (^4^F)^5^F	21 (^2^P)^3^D	15 (^4^F)^3^D
5	67731.94	.04	11	−268.	36 (^4^F)^5^G	15 (^4^F)^5^F	14 (^4^F)^3^G
0	68838.57	.11	3	−112.	29 (^4^F)^3^D	25 (^2^P)^3^P	22 (^2^P)^1^S
1	68976.80	.04	15	480.	27 (^4^F)^5^D	14 (^4^P)^5^D	11 (^2^P)^3^P
4	70221.35	.03	19	309.	33 (^4^F)^5^F	24 (^4^F)^5^D	11 (^4^F)^3^G
3	70681.63	.04	18	224.	34 (^4^F)^3^D	23 (^4^F)^3^G	11 (^4^F)^3^F
2	70762.26	.04	15	488.	54 (^4^F)^5^D	10 (^4^F)^3^F	7 (^4^P)^5^D
4	72073.75	.04	10	115.	31 (^4^F)^5^D	15 (^4^F)^3^G	14 (^2^G)^3^H
1	72425.51	.06	9	963.	44 (^4^F)^3^D	13 (^4^P)^3^P	9 (^2^P)^3^S
3	72881.50.	04	16	551.	37 (^4^F)^3^F	24 (^4^F)^3^G	14 (^2^G)^3^G
2	73135.83	.04	15	−1459.	40 (^4^F)^3^F	13 (^4^P)^3^D	12 (^4^F)^5^D
5	73289.66	.05	9	109.	59 (^4^F)^3^G	13 (^4^F)^3^F	8 (^2^G)^3^G
3	73697.35.	03	17	−637.	22 (^4^P)^5^D	15 (^2^P)^3^D	10 (^4^F)^5^D
1	73804.59	.05	11	192.	23 (^4^P)^5^D	23 (^4^F)^3^D	17 (^4^P)^3^P
4	74021.35	.03	19	280.	42 (^2^G)^3^H	34 (^4^F)^3^F	8 (^2^H)^3^H
2	74791.95	.04	19	−524.	31 (^4^F)^3^D	8 (^2^D2)^3^F	7 (^4^F)^5^D
0	75142.73	.05	4	458.	52 (^4^P)^5^D	30 (^2^P)^1^S	9 (^4^F)^3^D
6	75697.21	.06	5	678.	79 (^4^F)^3^G	18 (^2^G)^3^H	
5	75800.20	.03	16	236.	36 (^4^F)^5^F	30 (^2^H)^3^I	17 (^2^G)^3^H
2	76039.80	.04	18	−17.	18 ^3^F(^3^P)^5^G	16 (^4^P)^3^S	14 (^4^P)^5^P
3	76134.60	.03	23	−915.	21 (^2^G)^3^G	20 (^4^F)^5^D	15 (^4^F)^3^G
1	76378.09	.04	19	−456.	34 (^4^P)^3^D	25 (^2^P)^3^D	6 (^2^D2)^3^D
2	76831.39	.04	22	404.	21 (^4^P)^5^D	20 (^4^P)^5^P	16 ^3^F(^3^P)^5^G
3	77071.73	.04	21	−685.	28 (^4^F)^3^F	11 (^2^G)^1^F	7 (^2^D2)^3^F
2	77757.00	.04	22	−932.	35 ^3^F(^3^P)^5^G	8 (^2^D2)^3^F	8 (^4^F)^3^S
4	77827.64	.04	15	685.	23 (^2^H)^3^H	21 (^2^G)^1^G	19 (^4^F)^3^D
4	78408.35	.03	22	385.	54 (^4^F)^3^G	14 (^4^F)^5^F	7 (^2^H)^3^G
0	78507.70	.06	7	203.	43 (^2^P)^3^P	16 (^4^P)^3^P	12 (^2^D2)^3^P
2	78786.47	.04	18	−517.	32 (^4^P)^5^P	18 (^4^P)^5^D	10 (^2^P)^1^D
3	79139.81	.03	23	743.	16 ^3^F(^3^P)^5^D	14 (^2^G)^3^F	11 (^4^P)^5^D
1	79216.24	.05	13	−119.	35 (^4^P)^5^P	14 (^2^P)^3^D	10 (^2^D2)^3^D
4	79597.73	.04	19	41.	42 (^4^F)^3^F	18 (^2^G)^3^F	5 (^4^F)^3^G
3	79699.75	.04	21	361.	44 (^4^P)^5^P	15 (^2^P)^3^D	11 (^4^P)^5^D
5	80493.89	.04	15	−227.	32 (^4^F)^3^G	20 (^2^H)^3^H	19 (^4^F)^5^F
3	80558.62	.04	25	247.	28 ^3^F(^3^P)^5^G	24 (^4^P)^5^D	22 (^4^F)^3^D
6	81013.46	.05	9	−629.	51 (^2^H)^3^I	28 (^2^H)^3^H	
1	81054.33	.04	15	−45.	34 (^2^P)^3^S	27 (^4^P)^3^P	6 (^4^P)^5^P
2	81293.43	.04	21	207.	20 (^2^D2)^3^D	9 (^2^D2)^3^P	9 (^4^P)^5^D
1	82095.43	.04	17	−486.	53 ^3^F(^3^P)^5^F	17 (^2^P)^3^P	5 ^3^F(^3^P)^3^D
5	82210.83	.04	16	−171.	34 (^2^G)^3^H	32 (^2^H)^3^I	6 (^2^H)^1^H
4	82502.08	.03	24	164.	55 (^2^G)^3^G	10 (^2^G)^1^G	7 (^2^G)^3^H
2	82801.82	.04	20	188.	11 ^3^F(^3^P)^5^F	11 (^2^P)^1^D	9 (^2^F)^3^F
2	82980.19	.04	21	−470.	36 ^2^G(^3^F)	17 (^2^P)^3^D	9 (^4^P)^5^S
4	83571.04	.04	22	−109.	65 (^4^P)^5^D	6 (^2^D1)^3^F	6 ^3^F(^3^P)^5^G
1	83601.96	.06	13	350.	27 (^2^P)^1^P	12 (^2^D2)^3^P	10 (^4^P)^3^D
3	83611.69	.05	19	−240.	32 ^3^F(^3^P)^5^G	15 (^2^D2)^3^F	10 (^4^P)^5^D
5	83957.03	.04	14	110.	38 (^3^G)^3^G	13 (^2^G)^3^H	11 (^2^H)^3^H
3	84127.03	.04	25	−369.	25 (^2^G)^3^G	13 (^4^F)^3^F	12 (^4^P)^5^P
1	84389.26	.07	15	538.	29 (^2^P)^3^P	16 (^4^P)^3^P	11 (^4^P)^5^D
4	84869.01	.03	24	1.	28 (^2^H)^3^H	27 (^2^G)^3^F	9 (^2^G)^3^H
2	85044.18	.05	18	297.	22 ^3^F(^3^P)^5^F	18 (^4^P)^5^S	17 (^2^D2)^3^P
4	85078.28	.05	18	77.	53 ^3^F(^3^P)^5^G	11 (^4^P)^5^D	9 (^2^H)^3^H
5	85222.97	.05	16	200.	30 (^2^H)^3^H	24 (^4^F)^3^G	19 (^2^H)^3^G
2	85527.01	.04	23	408.	21 (^2^G)^3^F	11 (^2^P)^1^D	9 (^2^D2)^3^P
3	85834.94	.04	22	403.	18 (^2^G)^3^F	17 (^2^H)^3^G	8 (2D2)^3^F
6	85965.60	.06	7	671.	37 (^2^H)^1^I	36 (^2^G)^3^H	15 (^2^H)^3^H
4	86271.52	.04	24	−129.	17 (^2^H)^1^G	15 (^4^F)^3^F	13 ^3^F(^3^P)^5^G
3	86283.75	.03	28	63.	18 (^2^F)^3^F	9 (^2^F)^3^G	9 ^3^F(^3^P)^5^F
2	86487.10	.04	23	418.	27 ^3^F(^3^P)^5^F	12 (^4^P)^5^P	10 (^2^P)^3^D
3	86578.41	.04	25	−567.	16 (^2^D2)^3^D	15 (^2^F)^3^G	11 (^2^D2)^1^F
1	86865.05	.04	17	496.	16 (^4^P)^3^P	13 (^2^D2)^1^P	10 (^2^D2)^3^D
2	86867.50	.05	21	167.	18 (^2^F)^3^P	11 (^2^G)^3^F	8 (^4^P)^3^P
4	87590.69	.04	20	−23.	21 (^2^F)^3^F	13 (^2^F)^3^G	13 (^2^H)^3^G
2	87621.20	.04	20	−8.	26 (^4^P)^3^P	11 (^4^P)^3^D	9 ^3^F(^3^P)^5^D
5	87862.02	.04	18	−263.	48 (^2^G)'H	27 (^2^H)^3^G	7 (^2^H)^3^I
3	88429.94	.04	27	608.	24 ^3^F(^3^P)^5^F	12 (^2^G)^1^F	11 (^2^H)^3^I
3	88813.10	.04	26	418.	11 'D(^3^P)^3^D	9 (^2^D2)^3^D	9 ^3^F(^3^P)^3^D
0	88867.52	.13	4	−184.	54 ^3^F(^3^P)^5^D	13 ^1^D(^3^P)^3^P	11 ^3^P(^3^P)^5^D
6	88884.95	.06	8	−353.	44 (^2^H)^3^H	41 (^2^H)^3^I	10 (^2^G)^3^H
2	89240.38	.03	22	536.	24 (^4^P)^3^P	13 ^3^P(^3^P)^5^F	10 ^3^F(^3^P)^5^D
1	89283.42	.06	15	−161.	50 (^4^P)^3^D	15 (^2^D2)^3^D	5 ^3^F(^3^P)^3^F
3	89329.95	.04	22	22.	43 ^3^F(^3^P)^3^F	12 (^4^P)^3^D	7 (^2^D2)^3^D
5	89630.99	.04	15	−931.	77 ^3^F(^3^P)^5^G	6 ^3^F(^3^P)^3^F	
4	89700.42	.06	17	350.	28 (^2^D2)^3^F	9 ^1^D(^3^P)^3^F	8 (^2^H)^1^G
1	89903.01	.07	12	111.	57 ^3^F(^3^P)^5^D	7 ^3^F(^3^P)^5^F	6 ^3^F(^3^P)^3^D
3	90097.20	.03	30	−360.	17 (^2^F)^3^F	14 (^4^P)^3^D	11^3^F(^3^P)^3^F
1	90492.33	.05	18	−264.	31 (^2^D2)^1^P	15 (^4^P)^3^S	7(^2^D1)^1^P
7	90511.29	.17	2	281.	100 (^2^H)^3^I		
5	91161.95	.05	12	−354.	32 (^2^H)^1^H	25 (^2^G)^3^G	11 (^2^H)^3^H
2	91362.35	.05	21	23.	15 (^2^P)^3^P	12 (^2^D2)^3^D	10 (^2^D2)^1^D
3	91763.26	.05	16	−151.	26 (^2^H)^3^G	17 (^2^P)^3^D	9 (^2^D2)^1^F
4	91776.22	.06	16	−46.	60 ^3^F(^3^P)^3^F	11 (^2^D2)^3^F	10(^3^F)^3^G
2	91973.61	.05	17	293.	12 ^3^F0P)^3^F	9 (^4^P)^3^D	7 ^3^F(^1^P)^3^F
0	92089.69	.09	6	−62.	37 ^3^F(^3^P)^5^D	22 (^2^D2)^3^P	13 (^2^D1)^3^P
4	92117.89	.04	20	−138.	26 (^2^H)^1^G	17 (^2^H)^3^G	8 F(^3^P)^5^F
2	92358.71	.06	18	−949.	22 ^3^F(^3^P)^5^D	11 (^2^P)^3^P	9 (^4^P)^3^D
1	92882.06	.08	9	154.	22 ^3^P(^3^P)^5^D	19 (^4^P)^3^S	10 ^3^P(^1^P)^3^S
2	93226.03	.05	20	−1092.	16 (^2^D2)^1^D	16 (^4^P)^3^D	15 (^2^P)^1^D
3	93358.07	.04	27	−59.	23 (^2^D2)^3^D	19 (^4^P)^3^D	6 (^2^D2)^3^D
5	93778.68	.04	10	−95.	26 (^2^H)^3^G	23 (^2^H)^1^H	12 (^2^F)^3^G
1	93778.68	.04	16	−713.	45 ^3^P(^3^P)^5^D	9 (^4^P)^3^S	5 ^3^P(^3^P)^5^P
4	93867.25	.04	27	−188.	30 (^2^H)^3^G	13 (^2^G)^3^F	12 (^2^G)^1^G
6	93870.12	.09	7	310.	56 (^2^H)^1^I	23 (^2^G)^3^H	12 (^2^H)^3^H
4	94347.63	.05	20	21.	27 (^2^F)^3^G	17 (^2^F)^3^F	16 ^3^F(^3^P)^5^D
3	94508.46	.05	24	−239.	20 (^2^F)^3^D	11 ^3^P(^3^P)^3^D	11 (^2^F)^1^F
2	95134.61	.05	21	−404.	23 ^3^P(^3^P)^5^D	9 ^3^F(^3^P)^3^D	6 ^1^D(^3^P)^3^D
5	95137.13	.07	13	−81.	45 ^3^F(^3^P)^5^F	12 (^2^H)^1^H	11^3^F(^3^P)^3^G
1	95363.16	.06	15	−164.	18 ^3^F(^3^P)^5^D	15 (^2^P)^1^P	9 ^3^F(^1^P)^3^D
3	95422.00	.05	25	314.	36 ^3^F(^3^P)^5^D	10 ^3^P(^3^P)^5^D	8 ^1^D(^3^P)^3^F
1	96530.77	.07	15	551.	13 (^2^F)^3^D	9 ^3^F(^3^P)^3^D	9 ^3^P(^3^P)^5^P
2	96561.09	.05	21	141.	18 (^2^F)^3^F	17 (^2^F)^1^G	11 (^2^F)^3^D
3	96630.14	.05	21	−241.	20 (^2^G)^1^F	16 (^2^D2)^1^F	10 (^2^H)^3^G
4	96704.93	.04	24	848.	47 ^3^F(^3^P)^5^D	14 (^2^F)^3^F	8 ^3^F(^1^P)^3^F
6	97039.60	.09	8	327.	95 ^3^F(^3^F)^5^G		
4	97144.15	.05	21	−716.	25 (^2^F)^1^G	22 (^2^H)^1^G	20 ^3^F(^3^P)^1^G
5	97227.61	.07	15	360.	60 (^2^F)^3^G	16 ^3^P(^3^P)^5^F	5 ^3^F(^1^P)^3^G
3	97531.10	.06	18	−150.	24 (^2^F)^3^D	23 (^2^F)^1^F	20 ^3^F(^3^P)'F
2	98061.07	.05	20	−99.	17 ^3^F(^3^P)^5^D	13 (^2^F)^1^D	9 ^3^P(^3^P)^5^D
1	98188.14	.06	14	−94.	37 ^3^p(^3^P)^5^P	19 (^4^P)^3^S	16(^2^F)^3^D
3	99424.24	.06	21	355.	38 ^3^F(^1^P)^3^G	10 ^3^P(^3^P)^5^D	9 ^1^G(^3^P)^3^G
2	99814.69	.08	17	600.	17 ^3^P(^3^P)^5^D	14 ^3^P(^3^P)^5^P	14 (^2^F)^3^D
4	100202.96	.06	19	267.	15 ^3^F(^3^P)^3^G	14 ^1^G(^3^P)^3^H	9 ^1^D(^3^P)^3^F
1	101307.86	.06	15	278.	22 ^1^D(^3^P)^3^D	18 ^1^D(^3^P)^3^P	8 ^1^D(^1^P)^1^P
3	101383.26	.06	20	873.	27 ^3^F(^3^P)^3^F	20 ^3^F(^1^P)^3^F	8.^3^P(^3^P)^5^D
2	101947.18	.07	14	82.	16 (^2^D1)^3^F	12 ^3^P(^3^P)^5^S	10 (^2^D1)^3^D
1	102058.13	.09	9	−543.	25 (^2^D1)^3^D	14 (^2^D2)^3^D	13 ^3^P(^3^P)^3^D
3	102348.13	.06	18	−750.	33 ^3^P(^3^P)^5^D	25 F(^3^P)^3^D	10 ^3^F(^1^P)^3^G
2	102576.20	.06	16	135.	28 ^3^P(^3^P)^5^S	15 (^2^D1)^3^F	12 ^3^P(^3^P)^5^P
4	102826.51	.08	16	−431.	17 ^3^P(^3^P)^5^D	15 ^3^F(^1^P)^3^G	13 (^2^F)^1^G
3	103127.98	.06	20	−601.	20 (^2^D1)^3^D	15 (^2^D1)^3^F	8 ^3^P(^3^P)^5^D
1	103272.45	.05	10	−1800.	21 ^3^P(^3^P)^5^P	16 ^3^P(^3^P)^3^S	12 ^1^D(^3^P)^3^D
5	104191.52	.02	10	290.	28 ^1^G(^3^P)^3^H	21 ^3^F(^3^P)^1^F	20 ^1^G(^3^P)^3^G
2	104287.86	.06	18	437.	30 ^3^P(^3^P)^5^P	19 ^3^F('P)^3^D	13 ^3^F(^1^P)^3^F
2	104761.77	.07	14	519.	14 (^2^D1)^3^D	13 (^2^D1)^3^P	12 ^3^P(^3^P)^5^S
3	105033.31	.09	13	684.	30 ^1^D(^3^P)^3^F	21 ^1^G(^3^P)^3^G	10 ^3^F(^3^P)^3^D
4	105064.05	.08	14	−331.	31 ^3^F(^1^P)^3^G	15 ^1^G(^3^P)^3^G	10 ^3^F(^3^P)^3^F
4	105628.72	.07	16	−477.	32 1G(^3^P)^3^H	23 ^3^P(^3^P)^5^D	14 ^3^F(^3^P)^3^G
2	105670.02	.06	17	101.	22 ^1^D(^3^P)^3^P	16 (^2^D1)^3^P	10^3^P(^3^P)^3^S
3	106044.97	.06	17	−79.	30 ^1^D(^3^P)^3^P	21 ^1^G(^3^P)^3^G	10 ^3^P(^3^P)^3^D
1	106699.96	.10	10	−860.	22 (^2^D1)^3^P	20 ^3^F(^1^P)^3^D	13 ^3^F(^3^P)^3^D
3	107269.17	.06	20	−297.	14 ^1^G(^3^P)^3^G	13 ^1^D(^1^P)^1^F	12 ^3^F(^3^P)^3^G
2	107611.49	.07	12	245.	16 ^3^F(^1^P)^3^D	12 (^2^D1)^3^F	11(^2^D2)^3^F
4	107840.81	.07	15	−235.	27 ^1^G(^3^P)^3^F	23 ^3^G(^3^P)^3^G	14 ^3^F(^1^P)^3^G
3	108249.41	.06	16.	275.	16 ^3^F(^1^P)^3^D	13 (^2^D1)^3^F	12 ^3^F(^3^P)^1^F
1	109744.12	.09	8	235.	21 ^1^D(^3^P)^3^D	14 (^2^D1)^3^D	12 ^1^D(^3^P)^3^P
4	109818.37	.07	18	54.	21 ^1^G(^3^P)^3^H	20 ^1^F(^3^P)^1^G	12 ^3^F(^1^P)^3^D
2	109841.13	.09	13	516.	23 ^1^D(^3^P)^3^P	18 ^1^D(^3^P)^3^D	11^3^P(^3^P)^3^P
3	109950.38	.08	16	672.	20 ^3^P(^3^P)^5^P	15 ^1^D(^3^P)^3^D	9 ^3^F(^1^P)^3^D
2	110863.47	.08	15	394.	15 ^1^D(^3^P)^3^D	11 (^2^D1)^3^D	10 ^3^F(^1^P)^3^D
3	111063.06	.07	18	601.	24 ^3^F(^1^P)^3^F	21 (^2^D1)^1^F	8 ^3^F(^3^P)^3^F
4	111196.15	.08	14	−241.	49 (^2^D1)^3^F	15 ^3^F(^3^P)^1^G	14 ^3^P(^3^P)^5^D
3	111610.84	.07	19	−750.	24 ^1^D(^3^P)^3^D	7 ^3^F(^1^P)^3^D	7 ^3^P(^3^P)^5^P
6	112353.65	.16	3	−496.	94 ^1^G(^3^P)^3^H		
2	112844.05	.07	18	13.	28 (^2^D1)^1^D	21 (^2^D2)^1^D	15 (^2^F)^1^D
3	113709.40	.07	15	214.	15 ^3^F(^1^P)^3^D	14 ^3^P(^1^P)^3^D	l0 (^2^D1)^1^F
1	113730.91	.09	12	169.	25 (^2^D1)^1^P	19 ^3^P(^3^P)^3^P	11 (^2^F)^3^D
1	115619.98	.13	6	−67.	21 ^3^P(^1^P)^3^D	14 ^3^P(^3^P)^3^D	14 ^1^D(^1^P)^1^P
3	115795.95	.08	17	826.	19 (^2^D1)^3^D	14 ^1^D(^3^P)^3^D	10 ^3^F(^1^P)^3^F
2	116620.34	.09	13	132.	33 ^1^G(^3^P)^3^F	9 ^1^D(^1^P)^1^D	8 ^3^F(^3^P)^1^D
4	116735.40	.09	15	−937.	30 ^3^F(^1^P)^3^F	17 ^3^P(^3^P)^1^G	15 ^3^F(^3^P)^3^F
3	116774.06	.08	13	−114.	54 ^1^G(^3^P)^3^F	12 ^3^P(^3^P)^3^D	9 ^1^D(^1^P)^1^F
3	118243.40	.07	15	−687.	31 ^3^F(^1^P)^3^D	17 ^3^P(^1^P)^3^D	12 ^3^P(^3^P)^3^D
2	119448.46	.09	13	401.	36 ^1^G(^3^P)^3^F	11 ^3^P(^1^P)^3^D	11^3^F(^3^P)^1^D
5	120680.26	.12	7	51.	81 ^1^G(^1^P)^1^H	9 ^3^F(^1^P)^3^G	5 ^1^G(^3^P)^3^H
4	121296.58	.15	11	−742.	66 ^1^G(^1^P)^1^G	9 ^3^F(^1^P)^3^F	9 ^1^G(^3^P)^3^F
3	125865.71	.10	11	747.	40 ^3^P(^1^P)^3^D	18 ^1^G(^1^P)^1^F	12 ^3^P(^3^P)^3^D

**Table 6:** Observed and predicted number of levels for the reported configurations of W III

	Even configurations			Odd configurations	
	5*d*^4^*+*5*d*^3^6*s +* 5*d*^2^6*s*^2^			5*d*^1^6*p +* 5*d*^1^6*s*6*p*	
	Number of levels			Number of levels	
*J*	Observed	Predicted	*J*	Observed	Predicted
0	4	9	0	6	14
1	11	12	1	27	37
2	18	21	2	38	47
3	15	15	3	41	44
4	14	15	4	29	32
5	6	6	5	15	18
6	3	3	6	7	7
			7	1	1
			
Total	71	81	Total	164	200

**Table 7:** LSF and HF parameter values for the 5*d*^4^, 5*d*^3^ 6*s*, and 5*d*^2^ 6*s*^2^ configurations of doubly ionized tungsten (W III), in cm^−1^

Configuration	Parameter	LSF	HF	LSF/HF
5*d*^4^	*E*_av_	22339 (140)		
	*F*^2^(*dd*)	43442 (371)	56483	.77
	*F*^4^(*dd*)	28186 (315)	37195	.76
	*ζ_d_*	2364 (19)	2575	.92
	*α*	13 (3)		
	*β*	100 (69)		
5*d*^3^6*s*	*E*_av_	39566 (124)		
	*F*^2^(*dd*)	45990 (485)	59115	.78
	*F*^4^(*dd*)	29686 (391)	39110	.76
	*G*^2^(*ds*)	16130 (125)	15055	1.07
	*ζ_d_*	2542 (17)	2799	.91
	*α*	17 (4)		
	*β*	16 (65)		
5*d*^2^6*s*^2^	*E*_av_	55546 (1179)		
	*F*^2^(*dd*)	59344 (7308)	61460	.96
	*F*^4^(*dd*)	35759 (4070)	40862	.87
	*ζ_d_*	2616 (61)	3024	.87
	*α*	275 (151)		
	*β*	0 Fix		
C.I.	*R*^2^(*dd,ss*)	−18165 (307)	−17985.	1.01
	*R*^2^(*ds,ss*)	−20965 (133)	−18305.	1.14
	*R*^2^(*dd,ds*)	−21280 (343)	−18410.	1.15

Standard deviation is ±87 cm^−1^.

**Table 8:** LSF and HFR parameter values for the 5*d*^3^ 6*p* and 5*d*^2^ 6*s*6*p* configurations of doubly ionized tungsten (W III), in cm^−1^

Configuration	Parameter	LSF	HF	LSF/HF
5*d*^3^6*p*	*E*_av_	89431 (420)		
	*F*^2^(*dd*)	48290 (1500)	59619	.81
	*F*^4^(*dd*)	27390 (1250)	39488	.69
	*F*^2^(*dp*)	12169 (800)	21245	.57
	*G*^1^(*dp*)	8170 (270)	8926	.92
	*G*^3^(*dp*)	7400 Fix	7432	1.00
	ζ*_d_*	2739 (63)	2837	.97
	ζ*_p_*	5668 (139)	3660	1.55
	*α*	47 (11)		
	*β*	−300 Fix		
5*d*^2^ 6*s*6*p*	*E*_av_	115093 (436)		
	*F*^2^(*dd*)	48563 (3700)	61866	.78
	*F*^4^(*dd*)	27464 (3700)	41152	.67
	*F*^2^(*dp*)	16869 (1000)	22960	.73
	*G*^1^(*sp*)	41388 (1800)	41007	1.01
	*G*^2^(*ds*)	13643 (976)	15090	.90
	*G*^1^(*dp*)	10478 (478)	9240	1.13
	*G*^3^(*dp*)	7900 Fix	7922	1.00
	ζ*_d_*	3211 (95)	3057	1.05
	ζ*_p_*	5678 (240)	4044	1.40
	*α*	0 Fix		
	*β*	0 Fix		
C.I.	*R*^1^(*dp,ps*)	−3004 (392)	−22980	.13
	*R*^2^(*dp,sp*)	−8760 (520)	−14475	.61
	*R*^2^(*dd,ds*)	−21405 (772)	−18095	1.18

Standard deviation is ±450 cm ^−1^.
